# No effect of schizophrenia risk genes *MIR137*, *TCF4*, and *ZNF804A* on macroscopic brain structure

**DOI:** 10.1016/j.schres.2014.08.007

**Published:** 2014-11

**Authors:** Helena Cousijn, Marc Eissing, Guillén Fernández, Simon E. Fisher, Barbara Franke, Marcel Zwiers, Paul J. Harrison, Alejandro Arias-Vásquez

**Affiliations:** a Department of Psychiatry, University of Oxford, Oxford, UK; b Donders Institute for Brain, Cognition and Behaviour, Departments of Psychiatry, Human Genetics & Cognitive Neuroscience, Radboud University Medical Centre, Nijmegen, The Netherlands; c Donders Institute for Brain, Cognition and Behaviour, Radboud University Nijmegen, Nijmegen, The Netherlands; d Language and Genetics Department, Max Planck Institute for Psycholinguistics, Nijmegen, The Netherlands

**Keywords:** *miR-137*, *TCF4*, *ZNF804A*, Genetic neuroimaging, Brain volume

## Abstract

Single nucleotide polymorphisms (SNPs) within the *MIR137*, *TCF4*, and *ZNF804A* genes show genome-wide association to schizophrenia. However, the biological basis for the associations is unknown. Here, we tested the effects of these genes on brain structure in 1300 healthy adults. Using volumetry and voxel-based morphometry, neither gene-wide effects—including the combined effect of the genes—nor single SNP effects—including specific psychosis risk SNPs—were found on total brain volume, grey matter, white matter, or hippocampal volume. These results suggest that the associations between these risk genes and schizophrenia are unlikely to be mediated via effects on macroscopic brain structure.

## 1 Introduction

A recent meta-analysis of GWAS studies of schizophrenia found the strongest evidence for an association with the single nucleotide polymorphism (SNP) rs1625579 in an intron of the *MIR137* (http://www.genecards.org/cgi-bin/carddisp.pl?gene=mir137) gene (Ripke et al., 2011). This association was later replicated in a GWAS study, providing increasing evidence for a role for *MIR137* in the aetiology of schizophrenia (Ripke et al., 2013). Interestingly, *MIR137*, which encodes a microRNA, regulates several other schizophrenia risk genes, notably *ZNF804A* (http://www.genecards.org/cgi-bin/carddisp.pl?gene=ZNF804A) and *TCF4* (http://www.genecards.org/cgi-bin/carddisp.pl?gene=TCF4) (Kim et al., 2012, Guella et al., 2013, Kwon et al., 2013, Wright et al., 2013). In silico, cellular, and luciferase based approaches have provided evidence that MIR137 downregulates both ZNF804A (Kim et al., 2012) and TCF4 (Guella et al., 2013, Kwon et al., 2013) expression.

DNA sequence variation in *ZNF804A* and *TCF4* has been robustly associated with schizophrenia risk. SNP rs1344706, in intron two of *ZNF804A*, was the first variant to reach unequivocal genome-wide significance for schizophrenia (O'Donovan et al., 2008), with a later meta-analysis confirming the association and extending it to a broader psychosis phenotype (Williams et al., 2011). For *TCF4*, Stefansson et al. (2009) first reported an association between SNP rs9960767, in intron 3 of the gene, and schizophrenia, with several other SNPs in the gene subsequently being associated with the disorder (Ripke et al., 2011, Steinberg et al., 2011). *ZNF804A* and *TCF4* are believed to encode transcription factors, but little is known about the mechanistic pathways via which they might increase risk for schizophrenia. Similarly, the risk SNPs are located in non-coding regions of the genes and it is not yet clear how they modulate disease risk. At the molecular level, they may impact on transcript expression or splicing (Hill and Bray, 2012, Guella et al., 2013, Tao et al., 2014); at the systems level, the risk alleles may influence brain function or structure (Esslinger et al., 2009, Quednow et al., 2011, Rasetti et al., 2011, van Erp et al., 2014).

Structural MRI studies show that schizophrenia is associated with a reduction in total brain volume of around 2.6%, with larger effects for grey matter than white matter, and with the reductions prominent in frontal and temporal cortices and hippocampus (Fornito et al., 2009, Ellison-Wright and Bullmore, 2010, Haijma et al., 2013). For the SNPs under investigation in this study, only SNP rs1625579 in the *MIR137* gene has been associated with smaller hippocampal volumes in patients with schizophrenia (Lett et al., 2013). Therefore, we focused our investigations on total brain volume, grey matter, white matter, and hippocampal volume only.

Given the unambiguous genetic epidemiological evidence for the involvement of these three genes in the risk of schizophrenia, we investigated whether allelic variation in these genes impacts on macroscopic brain structure in a cohort of 1300 healthy adults. We assessed single SNP as well as gene-wide effects on our volumes of interest. We also investigated the *joint* effect of these three genes on these brain volumes (given the regulatory effects of *MIR137* on *ZNF804A* and *TCF4 (*Wright et al., 2013)). Additionally, using voxel-based morphometry (VBM), we studied whether risk SNPs rs1344706 in *ZNF804A*, rs9960767 in *TCF4*, and rs1625579 in *MIR137* were associated with variation in grey and white matter volume.

## 2 Methods

This study is part of the Brain Imaging Genetics (BIG) project which comprises healthy volunteer subjects, mostly university students, who participate in diverse imaging studies at the Donders Centre for Cognitive Neuroimaging (DCCN), Nijmegen, The Netherlands (Franke et al., 2010, Stein et al., 2012). At the time of this study, anatomical (T1-weighted) MRI scans and genetic data were available from 1300 self-reported healthy subjects, usually as part of their involvement in diverse smaller-scale studies at the DCCN. All had given their consent to participate in BIG. Demographic information about the cohort can be found in Table 1. The study was approved by the regional medical ethics committee (CMO Arnhem-Nijmegen).

**Table 1:** Demographic information, volumes in ml.

Total *N*	1300
Mean age (years)	22.9 (3.8)
Sex F/M	57.4/42.6%
Handedness (R)	93.6%
Mean TBV (SD)	1257.8 (125.5)
Mean GM volume (SD)	775.1 (81.4)
Mean WM volume (SD)	482.7 (62.1)
Mean Hippocampus volume (SD)	4.0 (0.4)

Genetic analyses, including genotyping, genetic imputation and quality control were performed as described previously (Guadalupe et al., 2014). Three different genes, *MIR137*, *TCF4*, and *ZNF804A* and approximately 25 kilobase flanking regions were analysed. A total of 1211 SNPs were included in the analysis (168 for *MIR137*, 146 for *TCF4*, and 897 for *ZNF804A*).

Magnetic resonance imaging (MRI) data were acquired at the Donders Centre for Cognitive Neuroimaging at 1.5T and 3T Siemens MRI scanners. Data acquisition and analysis have been described previously (Franke et al., 2010, Cousijn et al., 2012).

Statistical analysis on the volumetric data was performed using the –linear command implemented in PLINK software V1.07 (http://pngu.mgh.harvard.edu/~purcell/plink/) (Purcell et al., 2007). The method of analysis we performed was analogous to the method reported by Bralten et al., 2011, Bralten et al., 2013 and Cousijn et al. (2012). The analysis consisted of a SNP-by-SNP linear regression and the estimation of the effect of the complete gene. Additionally, we assessed the effect of the three genes combined. The SNP-by-SNP linear regression for association with brain volumes (GM, WM, TBV and hippocampal volume) was performed using sex, age, and TBV as covariates. The analysis of GM was adjusted by WM volume and vice-versa. Multiple testing correction was performed by running 10,000 max(T) permutation tests using the “mperm” command and obtaining an empirical *p*-value for each SNP. The association statistics of the observed and permuted data were saved using the “mperm-save-all” command and then added to create a Σstatistic per run for all SNPs at the same time (10,001 in total, one for the observed data and 10,000 for the permuted data). The empirical *p*-value was then estimated by the number of times the observed Σstatistic was bigger than the permuted Σstatistic divided by the total number of permutations (10,000). Results were considered significant at *p* < 0.05.

Additionally, VBM analyses were carried out for the schizophrenia risk SNPs rs1344706 in *ZNF804A*, rs9960767 in *TCF4*, and rs1625579 in *MIR137*. We also included in this analysis any other SNP showing significant association (empirical *p*-value corrected for multiple testing) with any of the volumes. A full-factorial ANCOVA with these SNPs as factor and participants' age, gender, TBV (where appropriate), and scan protocol as covariates was carried out. Statistics were corrected for non-stationarity and were applied at *p*(whole brain uncorrected) < .001 and subsequent cluster statistics at *p*(FWE) < .05. A Bonferroni correction was applied to correct for multiple comparisons.

## 3 Results

The gene-wide analysis of the individual genes as well as the three genes combined did not yield any significant results for association with TBV, GM volume, WM volume, or hippocampal volume (Table 2).

**Table 2:** Gene-wide *p*-values for the three genes separately and combined.

	TBV	GM	WM	Hippocampus
*ZNF804A*	0.4666	0.7560	0.9986	0.3868
*MIR137*	0.9271	0.8644	0.9971	0.1552
*TCF4*	0.9419	0.3158	0.4460	0.7306
All three genes combined	0.5543	0.8287	0.9089	0.3502

In the SNP-by-SNP analysis, one SNP in *MIR137*, rs9440302, was found to have a significant association with hippocampal volume (Supplementary Fig. 1), with an empirical *p*-value of 0.0166 (*p*_emp_ was estimated adjusting for 168 SNPs). Therefore, this SNP was also included in the VBM analysis). We looked specifically at the effects of SNPs rs1344706 in *ZNF804A*, rs9960767 in *TCF4*, and rs1625579 in *MIR137* on GM, WM, TBV and hippocampal volume but no significant effects were detected for these risk SNPs. The corrected *p*-values from the SNP-by-SNP analysis for these risk SNPs can be found in Supplementary Table 1. Corrected and uncorrected *p*-values for all studied SNPs can be found in Supplementary Table 2. When excluding left-handed subjects from our analyses, we found the same results.

Whole-brain VBM analyses revealed no effect of SNPs rs1344706 in *ZNF804A*, rs9960767 in *TCF4*, and rs1625579 in *MIR137* on GM or WM volumes. For SNP rs9440302 in *MIR137*, whole-brain analyses as well as a region of interest analysis within a hippocampal ROI (WFU pickatlas, Tzourio-Mazoyer et al., 2002) showed no effects.

## 4 Discussion

Here we studied the effects of schizophrenia risk genes *MIR137*, *TCF4*, and *ZNF804A* on macroscopic brain variation in healthy volunteers. No gene-wide effects or effects of specific risk SNPs were found on grey matter, white matter, total brain volume, and hippocampal volume. Additionally, when looking at the combined effect of the three risk genes, no effects could be detected. Given the large sample size used in this study (power to detect an effect size ≥ 5% with *n* = 1300; a SNP MAF = 19%; *D*′ = 0.5 with the causal variant; *α* = 0.05 is 80%), these findings provide convincing evidence that SNPs in these genes do not impact on macroscopic brain structure in young healthy volunteers.

This is the first study, to our knowledge, assessing the effects of SNPs in *TCF4* on TBV, GM, WM, and hippocampal volume. We also extended and replicated our earlier finding that variation in *ZNF804A* does not affect these parameters (increasing our sample size 46%: *n* = 892 vs *n* = 1300 see Cousijn et al., 2012; also Bergmann et al., 2013, Sprooten et al., 2012). Regarding *MIR137*, SNP rs1625579 has been previously associated with smaller hippocampal volumes in patients with schizophrenia (Lett et al., 2013), but we did not observe this effect in our sample of healthy controls. We did observe an association between SNP rs9440302 within *MIR137* and hippocampal volume, with the A allele being associated with larger hippocampal volumes. However, we could not replicate this finding using VBM, so future studies are needed to determine whether this is a robust association. Moreover, this SNP has not been associated with risk for schizophrenia and we found the probability of an association between this SNP and risk SNP rs1625579 to be very low (*r*^2^ = 0.203), indicating that they probably work independently.

Genetic neuroimaging studies in healthy volunteers provide important information about potential pathways between genetic variation and schizophrenia, by investigating the effects of risk genes on brain structure or brain function unconfounded by effects of the illness or its treatment. In this study, we have shown that genetic variation in schizophrenia risk genes *MIR137*, *TCF4*, and *ZNF804A* does not lead to alterations in TBV, GM, WM, or hippocampal brain volumes as measured with structural MRI in healthy young adults. Whilst we cannot rule out that effects might be found using different imaging methods such as diffusion tensor imaging, or that there might be effects in clinical populations or neurodevelopmental effects in childhood, it seems more likely that the pathophysiological correlates of allelic variation in these genes occur in other ways, such as via modulation of brain function or functional connectivity.

## Funding

This work makes use of the BIG (Brain Imaging Genetics) database, first established in Nijmegen, The Netherlands, in 2007. This resource is now part of Cognomics (www.cognomics.nl), a joint initiative by researchers of the Donders Institute for Brain, Cognition and Behaviour, the Human Genetics and Cognitive Neuroscience departments of the Radboud university medical centre and the Max Planck Institute for Psycholinguistics in Nijmegen. The Cognomics Initiative is supported by the participating departments and institutes and by external grants, i.e. the Biobanking and Biomolecular Resources Research Infrastructure (Netherlands) (BBMRI-NL), the Hersenstichting Nederland, and the Netherlands Organisation for Scientific Research (NWO). HC was funded by the 10.13039/100004440Wellcome Trust grant RQXU0. ME was funded by the Radboud Honours Programme Medical Sciences.

## Contributors

HC, PJH, and AAV designed the study. GF, SEF, BF, MZ, and AAV made the data available. ME and AAV carried out the analyses. HC wrote the first draft of the manuscript. All authors contributed to and have approved the final manuscript.

## Conflict of interest

All authors declare that they have no conflict of interest.
