# Supplementary material for: No effect of schizophrenia risk genes MIR137, TCF4, and ZNF804A on macroscopic brain structure
Source: Schizophr Res. 2014 Nov;159(2-3):329–32. doi: 10.1016/j.schres.2014.08.007 (PMC4245712; doi:10.1016/j.schres.2014.08.007)
Supplement: Supplementary file 1 — Supplementary material [file mmc1.docx]

**Supplementary material**

Supplementary Figure 1: Quantitative trait association between rs9440302 and hippocampal volume

Supplementary Table 1: Corrected p-values for effects of risk SNPs on the studied brain volumes

|  | MAF | TBV | GM | WM | Hippocampus |
| --- | --- | --- | --- | --- | --- |
| rs1344706 | C=0.345 | 1 | 1 | 1 | 1 |
| rs1625579 | G=0.188 | 1 | 1 | 0.9977 | 1 |
| rs9960767 | C=0.086 | 1 | 1 | 1 | 1 |

Supplementary Table 2: Association results between all SNPs in *MIR137*, *ZNF804A*, *TCF4* and brain volumes

| Gene | SNP | Position | WM | WM(Corr) | GM | GM(Corr) | TBV | TBV(Corr) | Hippo | Hippo(Corr) |
| --- | --- | --- | --- | --- | --- | --- | --- | --- | --- | --- |
| MIR137 | rs9659624 | 98487333 | 0.24 | 1.00 | 0.55 | 1.00 | 0.62 | 1.00 | 0.26 | 1.00 |
|  | rs5005705 | 98487510 | 0.24 | 1.00 | 0.55 | 1.00 | 0.62 | 1.00 | 0.26 | 1.00 |
|  | rs9324384 | 98487566 | 0.24 | 1.00 | 0.55 | 1.00 | 0.62 | 1.00 | 0.26 | 1.00 |
|  | rs79699616 | 98487629 | 0.21 | 0.99 | 0.45 | 1.00 | 0.71 | 1.00 | 0.87 | 1.00 |
|  | rs4634961 | 98487763 | 0.24 | 1.00 | 0.55 | 1.00 | 0.62 | 1.00 | 0.26 | 1.00 |
|  | rs9324385 | 98487899 | 0.24 | 1.00 | 0.55 | 1.00 | 0.62 | 1.00 | 0.26 | 1.00 |
|  | rs9324386 | 98487959 | 0.24 | 1.00 | 0.55 | 1.00 | 0.62 | 1.00 | 0.26 | 1.00 |
|  | rs9324387 | 98488030 | 0.24 | 1.00 | 0.55 | 1.00 | 0.62 | 1.00 | 0.26 | 1.00 |
|  | rs9324388 | 98488155 | 0.24 | 1.00 | 0.55 | 1.00 | 0.62 | 1.00 | 0.26 | 1.00 |
|  | rs79733093 | 98488509 | 0.78 | 1.00 | 0.73 | 1.00 | 0.35 | 1.00 | 0.31 | 1.00 |
|  | rs9659996 | 98488863 | 0.24 | 1.00 | 0.55 | 1.00 | 0.62 | 1.00 | 0.26 | 1.00 |
|  | rs9658841 | 98488895 | 0.24 | 1.00 | 0.55 | 1.00 | 0.62 | 1.00 | 0.26 | 1.00 |
|  | rs9661157 | 98489080 | 0.24 | 1.00 | 0.55 | 1.00 | 0.62 | 1.00 | 0.26 | 1.00 |
|  | rs10747496 | 98489330 | 0.24 | 1.00 | 0.55 | 1.00 | 0.62 | 1.00 | 0.26 | 1.00 |
|  | rs4584446 | 98489478 | 0.24 | 1.00 | 0.55 | 1.00 | 0.62 | 1.00 | 0.26 | 1.00 |
|  | rs2391905 | 98489735 | 0.24 | 1.00 | 0.55 | 1.00 | 0.62 | 1.00 | 0.26 | 1.00 |
|  | rs4950117 | 98490165 | 0.24 | 1.00 | 0.55 | 1.00 | 0.62 | 1.00 | 0.26 | 1.00 |
|  | rs4950118 | 98490358 | 0.24 | 1.00 | 0.55 | 1.00 | 0.62 | 1.00 | 0.26 | 1.00 |
|  | rs10783082 | 98490636 | 0.24 | 1.00 | 0.55 | 1.00 | 0.62 | 1.00 | 0.26 | 1.00 |
|  | rs75133093 | 98490668 | 0.25 | 1.00 | 0.16 | 1.00 | 0.44 | 1.00 | 0.04 | 0.63 |
|  | rs4950119 | 98491248 | 0.25 | 1.00 | 0.54 | 1.00 | 0.62 | 1.00 | 0.10 | 0.90 |
|  | rs4950120 | 98491382 | 0.24 | 1.00 | 0.55 | 1.00 | 0.62 | 1.00 | 0.26 | 1.00 |
|  | rs4245673 | 98491473 | 0.24 | 1.00 | 0.55 | 1.00 | 0.62 | 1.00 | 0.26 | 1.00 |
|  | rs1938568 | 98491732 | 0.24 | 1.00 | 0.55 | 1.00 | 0.62 | 1.00 | 0.26 | 1.00 |
|  | rs72735971 | 98491910 | 0.85 | 1.00 | 0.92 | 1.00 | 0.94 | 1.00 | 0.15 | 0.97 |
|  | rs9440393 | 98492462 | 0.24 | 1.00 | 0.55 | 1.00 | 0.62 | 1.00 | 0.26 | 1.00 |
|  | rs9440302 | 98492580 | 0.43 | 1.00 | 0.10 | 1.00 | 0.10 | 1.00 | 0.00 | 0.00 |
|  | rs78882372 | 98492609 | 0.91 | 1.00 | 0.64 | 1.00 | 0.38 | 1.00 | 0.10 | 0.88 |
|  | rs17117424 | 98492786 | 0.85 | 1.00 | 0.92 | 1.00 | 0.94 | 1.00 | 0.15 | 0.97 |
|  | rs9324389 | 98492854 | 0.24 | 1.00 | 0.55 | 1.00 | 0.62 | 1.00 | 0.26 | 1.00 |
|  | rs1938569 | 98493263 | 0.24 | 1.00 | 0.55 | 1.00 | 0.62 | 1.00 | 0.26 | 1.00 |
|  | rs2022864 | 98493785 | 0.24 | 1.00 | 0.50 | 1.00 | 0.69 | 1.00 | 0.28 | 1.00 |
|  | rs2022865 | 98493789 | 0.24 | 1.00 | 0.50 | 1.00 | 0.69 | 1.00 | 0.28 | 1.00 |
|  | rs35676984 | 98494171 | 0.78 | 1.00 | 1.00 | 1.00 | 0.72 | 1.00 | 0.08 | 0.85 |
|  | rs10875124 | 98494185 | 0.24 | 1.00 | 0.55 | 1.00 | 0.62 | 1.00 | 0.26 | 1.00 |
|  | rs10875125 | 98494200 | 0.24 | 1.00 | 0.55 | 1.00 | 0.62 | 1.00 | 0.26 | 1.00 |
|  | 1:98494388:I | 98494388 | 0.39 | 1.00 | 0.67 | 1.00 | 0.69 | 1.00 | 0.28 | 1.00 |
|  | rs1198574 | 98494606 | 0.24 | 1.00 | 0.55 | 1.00 | 0.62 | 1.00 | 0.26 | 1.00 |
|  | rs112185417 | 98494707 | 0.15 | 0.97 | 0.48 | 1.00 | 0.50 | 1.00 | 0.78 | 1.00 |
|  | rs1198573 | 98495513 | 0.24 | 1.00 | 0.55 | 1.00 | 0.62 | 1.00 | 0.26 | 1.00 |
|  | 1:98495934:D | 98495934 | 0.18 | 0.98 | 0.46 | 1.00 | 0.60 | 1.00 | 0.76 | 1.00 |
|  | 1:98495937:D | 98495937 | 0.18 | 0.98 | 0.46 | 1.00 | 0.60 | 1.00 | 0.76 | 1.00 |
|  | rs1198572 | 98497176 | 0.26 | 1.00 | 0.57 | 1.00 | 0.59 | 1.00 | 0.09 | 0.88 |
|  | rs1198571 | 98497672 | 0.25 | 1.00 | 0.57 | 1.00 | 0.59 | 1.00 | 0.24 | 1.00 |
|  | rs17371457 | 98498339 | 0.83 | 1.00 | 0.90 | 1.00 | 0.93 | 1.00 | 0.11 | 0.91 |
|  | rs11804556 | 98498441 | 0.18 | 0.98 | 0.46 | 1.00 | 0.60 | 1.00 | 0.76 | 1.00 |
|  | rs1782815 | 98498898 | 0.25 | 1.00 | 0.57 | 1.00 | 0.59 | 1.00 | 0.24 | 1.00 |
|  | rs148573777 | 98499631 | 0.88 | 1.00 | 0.64 | 1.00 | 0.56 | 1.00 | 0.41 | 1.00 |
|  | rs1782813 | 98499843 | 0.25 | 1.00 | 0.56 | 1.00 | 0.60 | 1.00 | 0.26 | 1.00 |
|  | rs7554283 | 98500045 | 0.06 | 0.76 | 0.02 | 0.99 | 0.20 | 1.00 | 0.01 | 0.25 |
|  | rs145098079 | 98500290 | 0.85 | 1.00 | 0.75 | 1.00 | 0.44 | 1.00 | 0.83 | 1.00 |
|  | rs17117459 | 98500533 | 0.85 | 1.00 | 0.92 | 1.00 | 0.94 | 1.00 | 0.15 | 0.97 |
|  | rs76649014 | 98500628 | 0.88 | 1.00 | 0.97 | 1.00 | 0.81 | 1.00 | 0.21 | 0.99 |
|  | rs190925241 | 98500649 | 0.18 | 0.99 | 0.33 | 1.00 | 0.95 | 1.00 | 0.96 | 1.00 |
|  | rs17117461 | 98500698 | 0.88 | 1.00 | 0.97 | 1.00 | 0.81 | 1.00 | 0.21 | 0.99 |
|  | rs17117466 | 98500928 | 0.88 | 1.00 | 0.97 | 1.00 | 0.81 | 1.00 | 0.21 | 0.99 |
|  | rs4950101 | 98501011 | 0.05 | 0.68 | 0.01 | 0.95 | 0.16 | 1.00 | 0.02 | 0.46 |
|  | rs17117472 | 98501264 | 0.88 | 1.00 | 0.97 | 1.00 | 0.81 | 1.00 | 0.21 | 0.99 |
|  | rs138848630 | 98501597 | 0.95 | 1.00 | 0.58 | 1.00 | 0.35 | 1.00 | 0.88 | 1.00 |
|  | rs1702294 | 98501984 | 0.26 | 1.00 | 0.60 | 1.00 | 0.56 | 1.00 | 0.11 | 0.93 |
|  | rs17117478 | 98502103 | 0.88 | 1.00 | 0.97 | 1.00 | 0.81 | 1.00 | 0.21 | 0.99 |
|  | rs1702293 | 98502159 | 0.58 | 1.00 | 0.85 | 1.00 | 0.70 | 1.00 | 0.22 | 1.00 |
|  | rs1782810 | 98502340 | 0.26 | 1.00 | 0.60 | 1.00 | 0.56 | 1.00 | 0.11 | 0.93 |
|  | rs1782811 | 98502410 | 0.25 | 1.00 | 0.56 | 1.00 | 0.60 | 1.00 | 0.26 | 1.00 |
|  | rs1628294 | 98502594 | 0.58 | 1.00 | 0.85 | 1.00 | 0.70 | 1.00 | 0.22 | 1.00 |
|  | rs72735981 | 98502695 | 0.88 | 1.00 | 0.97 | 1.00 | 0.81 | 1.00 | 0.21 | 0.99 |
|  | rs1625579 | 98502934 | 0.26 | 1.00 | 0.60 | 1.00 | 0.56 | 1.00 | 0.11 | 0.93 |
|  | rs78949299 | 98503955 | 0.88 | 1.00 | 0.97 | 1.00 | 0.81 | 1.00 | 0.21 | 0.99 |
|  | rs76871704 | 98504347 | 0.88 | 1.00 | 0.97 | 1.00 | 0.81 | 1.00 | 0.21 | 0.99 |
|  | rs145078673 | 98505035 | 0.88 | 1.00 | 0.97 | 1.00 | 0.81 | 1.00 | 0.21 | 0.99 |
|  | 1:98505589:I | 98505589 | 0.92 | 1.00 | 0.87 | 1.00 | 0.88 | 1.00 | 0.22 | 1.00 |
|  | rs75229359 | 98505736 | 0.88 | 1.00 | 0.97 | 1.00 | 0.81 | 1.00 | 0.21 | 0.99 |
|  | rs143674409 | 98506096 | 0.88 | 1.00 | 0.97 | 1.00 | 0.81 | 1.00 | 0.21 | 0.99 |
|  | rs72969621 | 98506270 | 0.88 | 1.00 | 0.97 | 1.00 | 0.81 | 1.00 | 0.21 | 0.99 |
|  | rs140920004 | 98506393 | 0.88 | 1.00 | 0.97 | 1.00 | 0.81 | 1.00 | 0.21 | 0.99 |
|  | rs139304127 | 98507431 | 0.91 | 1.00 | 0.67 | 1.00 | 0.60 | 1.00 | 0.21 | 0.99 |
|  | rs4292998 | 98507696 | 0.26 | 1.00 | 0.60 | 1.00 | 0.56 | 1.00 | 0.11 | 0.93 |
|  | rs4411173 | 98507718 | 0.25 | 1.00 | 0.56 | 1.00 | 0.60 | 1.00 | 0.26 | 1.00 |
|  | rs2802535 | 98508258 | 0.26 | 1.00 | 0.60 | 1.00 | 0.56 | 1.00 | 0.11 | 0.93 |
|  | rs138057001 | 98508595 | 0.88 | 1.00 | 0.97 | 1.00 | 0.81 | 1.00 | 0.21 | 0.99 |
|  | rs115354697 | 98508887 | 0.20 | 0.99 | 0.43 | 1.00 | 0.73 | 1.00 | 0.91 | 1.00 |
|  | rs116048198 | 98509003 | 0.88 | 1.00 | 0.97 | 1.00 | 0.81 | 1.00 | 0.21 | 0.99 |
|  | rs114845577 | 98509298 | 0.88 | 1.00 | 0.97 | 1.00 | 0.81 | 1.00 | 0.21 | 0.99 |
|  | rs148617624 | 98509438 | 0.88 | 1.00 | 0.97 | 1.00 | 0.81 | 1.00 | 0.21 | 0.99 |
|  | rs116790941 | 98509727 | 0.20 | 0.99 | 0.43 | 1.00 | 0.73 | 1.00 | 0.91 | 1.00 |
|  | rs114894221 | 98509934 | 0.88 | 1.00 | 0.97 | 1.00 | 0.81 | 1.00 | 0.21 | 0.99 |
|  | rs12744323 | 98510109 | 0.71 | 1.00 | 0.98 | 1.00 | 0.66 | 1.00 | 0.07 | 0.81 |
|  | rs76183049 | 98510315 | 0.20 | 0.99 | 0.43 | 1.00 | 0.73 | 1.00 | 0.91 | 1.00 |
|  | rs114402789 | 98510326 | 0.88 | 1.00 | 0.97 | 1.00 | 0.81 | 1.00 | 0.21 | 0.99 |
|  | rs74904371 | 98510847 | 0.88 | 1.00 | 0.97 | 1.00 | 0.81 | 1.00 | 0.21 | 0.99 |
|  | rs2660304 | 98512127 | 0.27 | 1.00 | 0.65 | 1.00 | 0.50 | 1.00 | 0.11 | 0.92 |
|  | rs78422095 | 98512350 | 0.88 | 1.00 | 0.97 | 1.00 | 0.81 | 1.00 | 0.21 | 0.99 |
|  | rs115108429 | 98513072 | 0.88 | 1.00 | 0.97 | 1.00 | 0.81 | 1.00 | 0.21 | 0.99 |
|  | rs2660303 | 98513144 | 0.57 | 1.00 | 0.72 | 1.00 | 0.88 | 1.00 | 0.18 | 0.99 |
|  | rs141931471 | 98513457 | 0.20 | 0.99 | 0.43 | 1.00 | 0.73 | 1.00 | 0.91 | 1.00 |
|  | 1:98513675:I | 98513675 | 0.43 | 1.00 | 0.73 | 1.00 | 0.64 | 1.00 | 0.15 | 0.97 |
|  | rs181510164 | 98513712 | 0.49 | 1.00 | 0.56 | 1.00 | 0.93 | 1.00 | 0.76 | 1.00 |
|  | rs61786696 | 98513845 | 0.58 | 1.00 | 0.85 | 1.00 | 0.70 | 1.00 | 0.22 | 1.00 |
|  | rs61786697 | 98513959 | 0.58 | 1.00 | 0.85 | 1.00 | 0.70 | 1.00 | 0.22 | 1.00 |
|  | 1:98514611:D | 98514611 | 0.71 | 1.00 | 0.79 | 1.00 | 0.98 | 1.00 | 0.38 | 1.00 |
|  | rs78977259 | 98516072 | 0.63 | 1.00 | 0.66 | 1.00 | 0.92 | 1.00 | 0.96 | 1.00 |
|  | 1:98516612:I | 98516612 | 0.71 | 1.00 | 0.82 | 1.00 | 0.91 | 1.00 | 0.13 | 0.95 |
|  | 1:98517292:D | 98517292 | 0.88 | 1.00 | 0.97 | 1.00 | 0.81 | 1.00 | 0.21 | 0.99 |
|  | rs76963373 | 98517797 | 0.88 | 1.00 | 0.97 | 1.00 | 0.81 | 1.00 | 0.21 | 0.99 |
|  | rs76372718 | 98518211 | 0.88 | 1.00 | 0.97 | 1.00 | 0.81 | 1.00 | 0.21 | 0.99 |
|  | rs115510655 | 98519377 | 0.88 | 1.00 | 0.97 | 1.00 | 0.81 | 1.00 | 0.21 | 0.99 |
|  | rs76866146 | 98520076 | 0.88 | 1.00 | 0.97 | 1.00 | 0.81 | 1.00 | 0.21 | 0.99 |
|  | rs2660302 | 98520219 | 0.38 | 1.00 | 0.71 | 1.00 | 0.59 | 1.00 | 0.06 | 0.75 |
|  | rs77674075 | 98521499 | 0.88 | 1.00 | 0.97 | 1.00 | 0.81 | 1.00 | 0.21 | 0.99 |
|  | rs2660301 | 98521585 | 0.41 | 1.00 | 0.83 | 1.00 | 0.47 | 1.00 | 0.10 | 0.90 |
|  | rs74646430 | 98521650 | 0.29 | 1.00 | 0.58 | 1.00 | 0.65 | 1.00 | 0.99 | 1.00 |
|  | 1:98521876:D | 98521876 | 0.88 | 1.00 | 0.97 | 1.00 | 0.81 | 1.00 | 0.21 | 0.99 |
|  | rs150253940 | 98523325 | 0.88 | 1.00 | 0.97 | 1.00 | 0.81 | 1.00 | 0.21 | 0.99 |
|  | rs145801776 | 98523590 | 0.88 | 1.00 | 0.97 | 1.00 | 0.81 | 1.00 | 0.21 | 0.99 |
|  | rs148973373 | 98523969 | 0.97 | 1.00 | 0.62 | 1.00 | 0.41 | 1.00 | 0.10 | 0.91 |
|  | rs78000828 | 98524347 | 0.88 | 1.00 | 0.97 | 1.00 | 0.81 | 1.00 | 0.21 | 0.99 |
|  | 1:98524628:D | 98524628 | 0.88 | 1.00 | 0.97 | 1.00 | 0.81 | 1.00 | 0.21 | 0.99 |
|  | rs1702292 | 98524960 | 0.37 | 1.00 | 0.82 | 1.00 | 0.40 | 1.00 | 0.03 | 0.53 |
|  | rs75269296 | 98525009 | 0.88 | 1.00 | 0.97 | 1.00 | 0.81 | 1.00 | 0.21 | 0.99 |
|  | rs1782818 | 98525221 | 0.41 | 1.00 | 0.86 | 1.00 | 0.43 | 1.00 | 0.04 | 0.60 |
|  | rs144601766 | 98525521 | 0.88 | 1.00 | 0.97 | 1.00 | 0.81 | 1.00 | 0.21 | 0.99 |
|  | rs78576334 | 98526015 | 0.88 | 1.00 | 0.97 | 1.00 | 0.81 | 1.00 | 0.21 | 0.99 |
|  | rs1702291 | 98526167 | 0.41 | 1.00 | 0.86 | 1.00 | 0.43 | 1.00 | 0.04 | 0.60 |
|  | rs76611862 | 98526773 | 0.88 | 1.00 | 0.97 | 1.00 | 0.81 | 1.00 | 0.21 | 0.99 |
|  | rs2802533 | 98527259 | 0.41 | 1.00 | 0.86 | 1.00 | 0.43 | 1.00 | 0.04 | 0.60 |
|  | rs10875127 | 98527414 | 0.11 | 0.90 | 0.27 | 1.00 | 0.78 | 1.00 | 0.08 | 0.84 |
|  | rs78040625 | 98527700 | 0.13 | 0.94 | 0.07 | 1.00 | 0.31 | 1.00 | 0.04 | 0.58 |
|  | rs12567114 | 98527951 | 0.39 | 1.00 | 0.25 | 1.00 | 0.44 | 1.00 | 0.10 | 0.91 |
|  | rs142469003 | 98528105 | 0.88 | 1.00 | 0.97 | 1.00 | 0.81 | 1.00 | 0.21 | 0.99 |
|  | rs145403806 | 98528142 | 0.13 | 0.94 | 0.07 | 1.00 | 0.31 | 1.00 | 0.04 | 0.58 |
|  | rs2660300 | 98528211 | 0.41 | 1.00 | 0.86 | 1.00 | 0.43 | 1.00 | 0.04 | 0.60 |
|  | rs2660299 | 98528452 | 0.41 | 1.00 | 0.86 | 1.00 | 0.43 | 1.00 | 0.04 | 0.60 |
|  | rs114326478 | 98528798 | 0.88 | 1.00 | 0.97 | 1.00 | 0.81 | 1.00 | 0.21 | 0.99 |
|  | rs2802532 | 98528830 | 0.62 | 1.00 | 0.89 | 1.00 | 0.69 | 1.00 | 0.22 | 1.00 |
|  | rs1198583 | 98529378 | 0.41 | 1.00 | 0.83 | 1.00 | 0.47 | 1.00 | 0.10 | 0.90 |
|  | rs115390393 | 98529585 | 0.83 | 1.00 | 0.54 | 1.00 | 0.48 | 1.00 | 0.68 | 1.00 |
|  | rs143612452 | 98529708 | 0.88 | 1.00 | 0.97 | 1.00 | 0.81 | 1.00 | 0.21 | 0.99 |
|  | rs148050011 | 98529865 | 0.88 | 1.00 | 0.97 | 1.00 | 0.81 | 1.00 | 0.21 | 0.99 |
|  | rs116797348 | 98530225 | 0.69 | 1.00 | 0.95 | 1.00 | 0.55 | 1.00 | 0.47 | 1.00 |
|  | rs80158081 | 98530384 | 0.88 | 1.00 | 0.97 | 1.00 | 0.81 | 1.00 | 0.21 | 0.99 |
|  | rs1198582 | 98530398 | 0.41 | 1.00 | 0.86 | 1.00 | 0.43 | 1.00 | 0.04 | 0.60 |
|  | rs1198581 | 98530559 | 0.41 | 1.00 | 0.86 | 1.00 | 0.43 | 1.00 | 0.04 | 0.60 |
|  | rs80348255 | 98530569 | 0.88 | 1.00 | 0.97 | 1.00 | 0.81 | 1.00 | 0.21 | 0.99 |
|  | rs1198580 | 98531022 | 0.41 | 1.00 | 0.86 | 1.00 | 0.43 | 1.00 | 0.04 | 0.60 |
|  | rs149760873 | 98531465 | 0.88 | 1.00 | 0.97 | 1.00 | 0.81 | 1.00 | 0.21 | 0.99 |
|  | rs114307916 | 98531764 | 0.88 | 1.00 | 0.97 | 1.00 | 0.81 | 1.00 | 0.21 | 0.99 |
|  | 1:98531920:I | 98531920 | 0.88 | 1.00 | 0.97 | 1.00 | 0.81 | 1.00 | 0.21 | 0.99 |
|  | rs115861610 | 98532318 | 0.88 | 1.00 | 0.97 | 1.00 | 0.81 | 1.00 | 0.21 | 0.99 |
|  | rs1198579 | 98532403 | 0.41 | 1.00 | 0.83 | 1.00 | 0.47 | 1.00 | 0.10 | 0.90 |
|  | rs1211661 | 98532848 | 0.62 | 1.00 | 0.89 | 1.00 | 0.69 | 1.00 | 0.22 | 1.00 |
|  | rs1848177 | 98532942 | 0.95 | 1.00 | 0.85 | 1.00 | 0.71 | 1.00 | 0.58 | 1.00 |
|  | rs150285005 | 98533044 | 0.67 | 1.00 | 0.61 | 1.00 | 0.77 | 1.00 | 0.79 | 1.00 |
|  | 1:98533323:D | 98533323 | 0.88 | 1.00 | 0.97 | 1.00 | 0.81 | 1.00 | 0.21 | 0.99 |
|  | rs1198600 | 98533392 | 0.41 | 1.00 | 0.83 | 1.00 | 0.47 | 1.00 | 0.10 | 0.90 |
|  | rs79441368 | 98533896 | 0.88 | 1.00 | 0.97 | 1.00 | 0.81 | 1.00 | 0.21 | 0.99 |
|  | rs114212116 | 98533960 | 0.42 | 1.00 | 0.13 | 1.00 | 0.14 | 1.00 | 0.05 | 0.68 |
|  | rs77974252 | 98534192 | 0.88 | 1.00 | 0.97 | 1.00 | 0.81 | 1.00 | 0.21 | 0.99 |
|  | rs116711437 | 98534292 | 0.88 | 1.00 | 0.97 | 1.00 | 0.81 | 1.00 | 0.21 | 0.99 |
|  | rs77530015 | 98534840 | 0.54 | 1.00 | 0.57 | 1.00 | 0.87 | 1.00 | 0.10 | 0.92 |
|  | rs1198598 | 98535375 | 0.41 | 1.00 | 0.86 | 1.00 | 0.43 | 1.00 | 0.04 | 0.60 |
|  | rs11808051 | 98535665 | 0.35 | 1.00 | 0.63 | 1.00 | 0.69 | 1.00 | 0.91 | 1.00 |
|  | 1:98536320:D | 98536320 | 0.32 | 1.00 | 0.29 | 1.00 | 0.64 | 1.00 | 0.30 | 1.00 |
|  | 1:98536321:D | 98536321 | 0.36 | 1.00 | 0.24 | 1.00 | 0.47 | 1.00 | 0.10 | 0.91 |
|  | rs78575518 | 98536351 | 0.85 | 1.00 | 0.92 | 1.00 | 0.94 | 1.00 | 0.15 | 0.97 |
|  | rs116104946 | 98536477 | 0.85 | 1.00 | 0.92 | 1.00 | 0.94 | 1.00 | 0.15 | 0.97 |
|  | 1:98536723:D | 98536723 | 0.37 | 1.00 | 0.65 | 1.00 | 0.67 | 1.00 | 0.08 | 0.86 |
|  | rs1782816 | 98536724 | 0.37 | 1.00 | 0.65 | 1.00 | 0.67 | 1.00 | 0.08 | 0.86 |
|  | rs1702285 | 98536725 | 0.39 | 1.00 | 0.70 | 1.00 | 0.63 | 1.00 | 0.07 | 0.80 |
| TCF4 | rs9960767 | 53155002 | 1.00 | 1.00 | 1.00 | 1.00 | 1.00 | 1.00 | 1.00 | 1.00 |
|  | rs584564 | 53227648 | 0.95 | 1.00 | 0.58 | 1.00 | 0.96 | 1.00 | 0.64 | 1.00 |
|  | rs188932552 | 53229164 | 0.77 | 1.00 | 0.88 | 1.00 | 0.10 | 1.00 | 0.74 | 1.00 |
|  | rs72932743 | 53229628 | 0.74 | 1.00 | 0.37 | 1.00 | 0.91 | 1.00 | 0.69 | 1.00 |
|  | rs17598416 | 53229761 | 0.05 | 0.89 | 0.99 | 1.00 | 0.00 | 0.20 | 0.65 | 1.00 |
|  | rs112945363 | 53229786 | 0.32 | 1.00 | 0.62 | 1.00 | 0.89 | 1.00 | 0.37 | 1.00 |
|  | rs66791238 | 53230650 | 0.83 | 1.00 | 0.99 | 1.00 | 0.57 | 1.00 | 0.52 | 1.00 |
|  | rs28607662 | 53230859 | 0.64 | 1.00 | 0.85 | 1.00 | 0.69 | 1.00 | 0.87 | 1.00 |
|  | rs34533676 | 53234728 | 0.70 | 1.00 | 0.62 | 1.00 | 0.61 | 1.00 | 0.79 | 1.00 |
|  | 18:53235579:D | 53235579 | 0.03 | 0.74 | 0.94 | 1.00 | 0.58 | 1.00 | 0.72 | 1.00 |
|  | rs113055896 | 53238550 | 0.27 | 1.00 | 0.99 | 1.00 | 0.97 | 1.00 | 0.11 | 1.00 |
|  | rs56839141 | 53239287 | 0.79 | 1.00 | 0.76 | 1.00 | 0.09 | 1.00 | 0.63 | 1.00 |
|  | rs8098032 | 53239302 | 0.94 | 1.00 | 0.37 | 1.00 | 0.89 | 1.00 | 0.95 | 1.00 |
|  | rs72627232 | 53239916 | 0.89 | 1.00 | 0.43 | 1.00 | 0.32 | 1.00 | 0.33 | 1.00 |
|  | rs41396445 | 53242137 | 0.21 | 1.00 | 0.62 | 1.00 | 0.15 | 1.00 | 0.34 | 1.00 |
|  | rs74509138 | 53242546 | 0.65 | 1.00 | 0.62 | 1.00 | 0.82 | 1.00 | 0.69 | 1.00 |
|  | 18:53242804:D | 53242804 | 0.72 | 1.00 | 0.88 | 1.00 | 0.81 | 1.00 | 0.57 | 1.00 |
|  | rs11151167 | 53242807 | 0.86 | 1.00 | 0.39 | 1.00 | 0.79 | 1.00 | 0.89 | 1.00 |
|  | 18:53243426:I | 53243426 | 0.64 | 1.00 | 0.85 | 1.00 | 0.30 | 1.00 | 0.30 | 1.00 |
|  | rs2924322 | 53244414 | 0.85 | 1.00 | 0.99 | 1.00 | 0.56 | 1.00 | 0.94 | 1.00 |
|  | 18:53244442:D | 53244442 | 0.92 | 1.00 | 0.37 | 1.00 | 0.34 | 1.00 | 0.71 | 1.00 |
|  | rs117439929 | 53246366 | 0.77 | 1.00 | 0.37 | 1.00 | 0.10 | 1.00 | 0.74 | 1.00 |
|  | 18:53246696:D | 53246696 | 0.76 | 1.00 | 0.58 | 1.00 | 0.19 | 1.00 | 0.40 | 1.00 |
|  | rs618869 | 53248151 | 0.93 | 1.00 | 0.87 | 1.00 | 0.44 | 1.00 | 0.78 | 1.00 |
|  | rs12456652 | 53250050 | 0.99 | 1.00 | 0.74 | 1.00 | 0.76 | 1.00 | 0.87 | 1.00 |
|  | rs79703067 | 53250324 | 0.30 | 1.00 | 0.65 | 1.00 | 0.25 | 1.00 | 0.34 | 1.00 |
|  | 18:53250993:D | 53250993 | 0.99 | 1.00 | 0.65 | 1.00 | 0.76 | 1.00 | 0.87 | 1.00 |
|  | rs17598729 | 53251562 | 0.99 | 1.00 | 0.70 | 1.00 | 0.76 | 1.00 | 0.87 | 1.00 |
|  | rs117818068 | 53251689 | 0.98 | 1.00 | 0.93 | 1.00 | 0.90 | 1.00 | 0.90 | 1.00 |
|  | rs4144686 | 53251725 | 0.99 | 1.00 | 0.70 | 1.00 | 0.76 | 1.00 | 0.87 | 1.00 |
|  | 18:53251751:D | 53251751 | 0.99 | 1.00 | 0.93 | 1.00 | 0.76 | 1.00 | 0.87 | 1.00 |
|  | rs12455881 | 53252285 | 0.99 | 1.00 | 0.93 | 1.00 | 0.76 | 1.00 | 0.87 | 1.00 |
|  | rs599550 | 53252388 | 0.92 | 1.00 | 0.65 | 1.00 | 0.43 | 1.00 | 0.90 | 1.00 |
|  | rs34603418 | 53252889 | 0.99 | 1.00 | 0.74 | 1.00 | 0.76 | 1.00 | 0.87 | 1.00 |
|  | rs191615744 | 53253163 | 0.77 | 1.00 | 0.57 | 1.00 | 0.10 | 1.00 | 0.74 | 1.00 |
|  | rs12954356 | 53253410 | 0.86 | 1.00 | 0.65 | 1.00 | 0.65 | 1.00 | 0.92 | 1.00 |
|  | rs140040330 | 53253414 | 0.47 | 1.00 | 0.37 | 1.00 | 0.51 | 1.00 | 0.22 | 1.00 |
|  | rs143877298 | 53253417 | 0.70 | 1.00 | 0.92 | 1.00 | 0.19 | 1.00 | 0.54 | 1.00 |
|  | rs145850351 | 53253420 | 0.56 | 1.00 | 0.82 | 1.00 | 0.08 | 1.00 | 0.24 | 1.00 |
|  | rs149011565 | 53253423 | 0.74 | 1.00 | 0.90 | 1.00 | 0.12 | 1.00 | 0.96 | 1.00 |
|  | rs143743309 | 53253431 | 0.99 | 1.00 | 0.83 | 1.00 | 0.76 | 1.00 | 0.87 | 1.00 |
|  | rs75754713 | 53254028 | 0.02 | 0.71 | 0.71 | 0.94 | 0.26 | 1.00 | 0.39 | 1.00 |
|  | rs72932757 | 53254741 | 0.35 | 1.00 | 0.64 | 1.00 | 0.61 | 1.00 | 1.00 | 1.00 |
|  | rs184462159 | 53255244 | 0.06 | 0.93 | 0.39 | 1.00 | 0.40 | 1.00 | 0.02 | 0.93 |
|  | rs189089248 | 53257142 | 0.77 | 1.00 | 0.99 | 1.00 | 0.10 | 1.00 | 0.74 | 1.00 |
|  | rs12457949 | 53258609 | 0.91 | 1.00 | 0.53 | 1.00 | 0.94 | 1.00 | 0.88 | 1.00 |
|  | rs13381608 | 53259146 | 0.75 | 1.00 | 0.62 | 1.00 | 0.90 | 1.00 | 0.65 | 1.00 |
|  | rs183158209 | 53260357 | 0.74 | 1.00 | 0.79 | 1.00 | 0.53 | 1.00 | 0.70 | 1.00 |
|  | rs590076 | 53260732 | 0.18 | 1.00 | 0.62 | 1.00 | 0.12 | 1.00 | 0.29 | 1.00 |
|  | rs34556259 | 53261217 | 0.91 | 1.00 | 0.50 | 1.00 | 0.94 | 1.00 | 0.88 | 1.00 |
|  | rs57884215 | 53262184 | 0.91 | 1.00 | 0.50 | 1.00 | 0.94 | 1.00 | 0.88 | 1.00 |
|  | rs596668 | 53264343 | 0.95 | 1.00 | 0.34 | 1.00 | 0.47 | 1.00 | 0.70 | 1.00 |
|  | rs66485076 | 53264346 | 0.91 | 1.00 | 0.99 | 1.00 | 0.94 | 1.00 | 0.88 | 1.00 |
|  | 18:53264629:D | 53264629 | 0.88 | 1.00 | 0.97 | 1.00 | 0.47 | 1.00 | 0.44 | 1.00 |
|  | rs145451952 | 53265063 | 0.80 | 1.00 | 0.62 | 1.00 | 0.54 | 1.00 | 0.14 | 1.00 |
|  | rs17061809 | 53267742 | 0.67 | 1.00 | 0.50 | 1.00 | 0.33 | 1.00 | 0.14 | 1.00 |
|  | rs76234471 | 53267952 | 0.74 | 1.00 | 0.62 | 1.00 | 0.53 | 1.00 | 0.70 | 1.00 |
|  | rs113477183 | 53268175 | 0.61 | 1.00 | 0.35 | 1.00 | 0.58 | 1.00 | 0.60 | 1.00 |
|  | rs35178579 | 53268923 | 0.91 | 1.00 | 0.62 | 1.00 | 0.94 | 1.00 | 0.88 | 1.00 |
|  | rs17514172 | 53269093 | 0.91 | 1.00 | 0.81 | 1.00 | 0.94 | 1.00 | 0.88 | 1.00 |
|  | rs34341606 | 53269352 | 0.91 | 1.00 | 0.39 | 1.00 | 0.94 | 1.00 | 0.88 | 1.00 |
|  | rs682245 | 53270531 | 0.22 | 1.00 | 0.56 | 1.00 | 0.23 | 1.00 | 0.32 | 1.00 |
|  | 18:53270620:D | 53270620 | 0.91 | 1.00 | 0.84 | 1.00 | 0.94 | 1.00 | 0.88 | 1.00 |
|  | rs17514242 | 53270902 | 0.18 | 1.00 | 0.66 | 1.00 | 0.12 | 1.00 | 0.29 | 1.00 |
|  | 18:53271457:D | 53271457 | 0.96 | 1.00 | 0.53 | 1.00 | 0.83 | 1.00 | 0.97 | 1.00 |
|  | rs12455205 | 53272160 | 0.91 | 1.00 | 0.24 | 1.00 | 0.94 | 1.00 | 0.88 | 1.00 |
|  | rs79818298 | 53272228 | 0.77 | 1.00 | 0.55 | 1.00 | 0.10 | 1.00 | 0.74 | 1.00 |
|  | rs57756389 | 53273496 | 0.68 | 1.00 | 0.85 | 1.00 | 0.35 | 1.00 | 0.87 | 1.00 |
|  | rs635538 | 53273614 | 0.88 | 1.00 | 0.46 | 1.00 | 0.88 | 1.00 | 0.72 | 1.00 |
|  | rs17089898 | 53274828 | 0.79 | 1.00 | 0.85 | 1.00 | 0.11 | 1.00 | 0.60 | 1.00 |
|  | rs12458118 | 53276116 | 0.22 | 1.00 | 0.37 | 1.00 | 0.19 | 1.00 | 0.36 | 1.00 |
|  | rs9951280 | 53276523 | 0.22 | 1.00 | 0.24 | 1.00 | 0.19 | 1.00 | 0.36 | 1.00 |
|  | rs10871582 | 53276589 | 0.15 | 1.00 | 0.90 | 1.00 | 0.10 | 1.00 | 0.29 | 1.00 |
|  | 18:53277143:D | 53277143 | 0.15 | 1.00 | 0.97 | 1.00 | 0.10 | 1.00 | 0.29 | 1.00 |
|  | rs2156008 | 53281244 | 0.88 | 1.00 | 0.98 | 1.00 | 0.17 | 1.00 | 0.56 | 1.00 |
|  | 18:53281681:D | 53281681 | 0.03 | 0.74 | 0.41 | 1.00 | 0.58 | 1.00 | 0.72 | 1.00 |
|  | 18:53282717:D | 53282717 | 0.77 | 1.00 | 0.98 | 1.00 | 0.10 | 1.00 | 0.74 | 1.00 |
|  | rs616580 | 53284109 | 0.22 | 1.00 | 0.37 | 1.00 | 0.19 | 1.00 | 0.36 | 1.00 |
|  | rs116971134 | 53284330 | 0.74 | 1.00 | 0.77 | 1.00 | 0.53 | 1.00 | 0.70 | 1.00 |
|  | rs117789202 | 53285397 | 0.79 | 1.00 | 0.54 | 1.00 | 0.32 | 1.00 | 0.18 | 1.00 |
|  | rs1642313 | 53287570 | 0.21 | 1.00 | 0.77 | 1.00 | 0.17 | 1.00 | 0.36 | 1.00 |
|  | rs72932792 | 53288026 | 0.69 | 1.00 | 0.35 | 1.00 | 0.98 | 1.00 | 0.68 | 1.00 |
|  | rs78526562 | 53288175 | 0.91 | 1.00 | 0.69 | 1.00 | 0.16 | 1.00 | 0.72 | 1.00 |
|  | 18:53288640:I | 53288640 | 0.69 | 1.00 | 0.59 | 1.00 | 0.75 | 1.00 | 0.61 | 1.00 |
|  | rs186911106 | 53288876 | 0.65 | 1.00 | 0.77 | 1.00 | 0.35 | 1.00 | 0.05 | 1.00 |
|  | rs191256070 | 53288879 | 0.38 | 1.00 | 0.82 | 1.00 | 0.16 | 1.00 | 0.71 | 1.00 |
|  | 18:53289076:D | 53289076 | 0.57 | 1.00 | 0.43 | 1.00 | 0.04 | 1.00 | 0.14 | 1.00 |
|  | rs142117874 | 53289092 | 0.99 | 1.00 | 0.22 | 1.00 | 0.31 | 1.00 | 0.52 | 1.00 |
|  | 18:53289237:D | 53289237 | 0.94 | 1.00 | 0.79 | 1.00 | 0.13 | 1.00 | 0.61 | 1.00 |
|  | 18:53289421:D | 53289421 | 0.75 | 1.00 | 0.38 | 1.00 | 0.13 | 1.00 | 0.74 | 1.00 |
|  | rs149951154 | 53290537 | 0.77 | 1.00 | 0.26 | 1.00 | 0.10 | 1.00 | 0.74 | 1.00 |
|  | rs192745372 | 53290548 | 0.77 | 1.00 | 0.97 | 1.00 | 0.10 | 1.00 | 0.74 | 1.00 |
|  | rs118134308 | 53290877 | 0.02 | 0.62 | 0.97 | 1.00 | 0.25 | 1.00 | 0.29 | 1.00 |
|  | rs17514601 | 53292430 | 0.21 | 1.00 | 0.75 | 1.00 | 0.05 | 1.00 | 0.94 | 1.00 |
|  | rs8090341 | 53292908 | 0.36 | 1.00 | 0.24 | 1.00 | 0.08 | 1.00 | 0.22 | 1.00 |
|  | rs181313293 | 53293677 | 0.77 | 1.00 | 0.38 | 1.00 | 0.10 | 1.00 | 0.74 | 1.00 |
|  | rs644279 | 53294569 | 0.26 | 1.00 | 0.64 | 1.00 | 0.18 | 1.00 | 0.24 | 1.00 |
|  | rs183934016 | 53295223 | 0.77 | 1.00 | 0.38 | 1.00 | 0.10 | 1.00 | 0.74 | 1.00 |
|  | rs148012797 | 53295245 | 0.73 | 1.00 | 0.24 | 1.00 | 0.87 | 1.00 | 0.36 | 1.00 |
|  | 18:53296360:D | 53296360 | 0.89 | 1.00 | 0.42 | 1.00 | 0.49 | 1.00 | 0.38 | 1.00 |
|  | rs117343880 | 53296532 | 0.63 | 1.00 | 0.38 | 1.00 | 0.10 | 1.00 | 0.66 | 1.00 |
|  | rs12457157 | 53296927 | 0.29 | 1.00 | 0.39 | 1.00 | 0.10 | 1.00 | 0.21 | 1.00 |
|  | rs17527346 | 53297991 | 0.02 | 0.62 | 0.77 | 1.00 | 0.25 | 1.00 | 0.29 | 1.00 |
|  | 18:53298806:D | 53298806 | 0.06 | 0.92 | 0.85 | 0.99 | 0.24 | 1.00 | 0.73 | 1.00 |
|  | rs117286999 | 53299300 | 0.77 | 1.00 | 0.45 | 1.00 | 0.10 | 1.00 | 0.74 | 1.00 |
|  | rs112311990 | 53299332 | 0.02 | 0.64 | 0.34 | 1.00 | 0.19 | 1.00 | 0.49 | 1.00 |
|  | rs12103984 | 53299460 | 0.26 | 1.00 | 0.84 | 1.00 | 0.18 | 1.00 | 0.24 | 1.00 |
|  | rs78294462 | 53301359 | 0.02 | 0.62 | 0.06 | 1.00 | 0.25 | 1.00 | 0.29 | 1.00 |
|  | rs190710584 | 53301432 | 0.84 | 1.00 | 0.78 | 1.00 | 0.92 | 1.00 | 0.08 | 1.00 |
|  | rs6566169 | 53301527 | 0.29 | 1.00 | 0.79 | 1.00 | 0.10 | 1.00 | 0.21 | 1.00 |
|  | rs111935424 | 53301794 | 0.67 | 1.00 | 0.78 | 1.00 | 0.45 | 1.00 | 0.48 | 1.00 |
|  | rs7236100 | 53302321 | 0.79 | 1.00 | 0.78 | 1.00 | 0.49 | 1.00 | 0.31 | 1.00 |
|  | rs77891683 | 53303150 | 0.34 | 1.00 | 0.78 | 1.00 | 0.15 | 1.00 | 0.46 | 1.00 |
|  | rs35691742 | 53303546 | 0.29 | 1.00 | 0.99 | 1.00 | 0.10 | 1.00 | 0.21 | 1.00 |
|  | rs8096777 | 53303616 | 0.75 | 1.00 | 0.78 | 1.00 | 0.47 | 1.00 | 0.32 | 1.00 |
|  | rs34733940 | 53303684 | 0.80 | 1.00 | 0.45 | 1.00 | 0.91 | 1.00 | 0.99 | 1.00 |
|  | rs72934723 | 53305311 | 0.94 | 1.00 | 0.01 | 1.00 | 0.34 | 1.00 | 0.21 | 1.00 |
|  | rs12458015 | 53305735 | 0.18 | 1.00 | 0.78 | 1.00 | 0.09 | 1.00 | 0.19 | 1.00 |
|  | 18:53308061:D | 53308061 | 0.27 | 1.00 | 0.78 | 1.00 | 0.15 | 1.00 | 0.30 | 1.00 |
|  | rs1421024 | 53309114 | 0.69 | 1.00 | 0.96 | 1.00 | 0.11 | 1.00 | 0.31 | 1.00 |
|  | rs1421025 | 53309363 | 0.69 | 1.00 | 0.78 | 1.00 | 0.11 | 1.00 | 0.31 | 1.00 |
|  | rs660010 | 53310969 | 0.94 | 1.00 | 0.78 | 1.00 | 0.37 | 1.00 | 0.85 | 1.00 |
|  | rs76888488 | 53311001 | 0.03 | 0.78 | 0.91 | 1.00 | 0.61 | 1.00 | 0.93 | 1.00 |
|  | rs9946669 | 53311559 | 0.69 | 1.00 | 0.41 | 1.00 | 0.11 | 1.00 | 0.28 | 1.00 |
|  | rs111652772 | 53313003 | 0.03 | 0.74 | 0.78 | 1.00 | 0.96 | 1.00 | 0.61 | 1.00 |
|  | rs652749 | 53314500 | 0.96 | 1.00 | 0.77 | 1.00 | 0.98 | 1.00 | 0.57 | 1.00 |
|  | rs79526326 | 53314519 | 0.21 | 1.00 | 0.92 | 1.00 | 0.42 | 1.00 | 0.97 | 1.00 |
|  | rs17089911 | 53315397 | 0.30 | 1.00 | 0.78 | 1.00 | 0.08 | 1.00 | 0.25 | 1.00 |
|  | rs663118 | 53316832 | 0.69 | 1.00 | 0.78 | 1.00 | 0.11 | 1.00 | 0.28 | 1.00 |
|  | rs11151206 | 53316911 | 0.69 | 1.00 | 0.95 | 1.00 | 0.11 | 1.00 | 0.28 | 1.00 |
|  | 18:53319419:D | 53319419 | 0.72 | 1.00 | 0.32 | 1.00 | 0.14 | 1.00 | 0.20 | 1.00 |
|  | rs619442 | 53319703 | 0.65 | 1.00 | 0.25 | 1.00 | 0.13 | 1.00 | 0.26 | 1.00 |
|  | rs605951 | 53320447 | 0.65 | 1.00 | 0.69 | 1.00 | 0.13 | 1.00 | 0.26 | 1.00 |
|  | rs651350 | 53320738 | 0.94 | 1.00 | 0.92 | 1.00 | 0.37 | 1.00 | 0.88 | 1.00 |
|  | rs144886295 | 53320864 | 0.70 | 1.00 | 0.99 | 1.00 | 0.90 | 1.00 | 0.36 | 1.00 |
|  | rs652714 | 53321026 | 0.94 | 1.00 | 0.24 | 1.00 | 0.37 | 1.00 | 0.88 | 1.00 |
|  | rs77057028 | 53321069 | 0.14 | 1.00 | 0.99 | 1.00 | 0.98 | 1.00 | 0.94 | 1.00 |
|  | rs56142542 | 53321323 | 0.65 | 1.00 | 0.94 | 1.00 | 0.13 | 1.00 | 0.26 | 1.00 |
|  | rs9956444 | 53323367 | 0.68 | 1.00 | 0.24 | 1.00 | 0.35 | 1.00 | 0.87 | 1.00 |
|  | rs652447 | 53323519 | 0.65 | 1.00 | 0.24 | 1.00 | 0.13 | 1.00 | 0.26 | 1.00 |
|  | 18:53325002:D | 53325002 | 0.65 | 1.00 | 0.78 | 1.00 | 0.13 | 1.00 | 0.26 | 1.00 |
|  | 18:53325378:D | 53325378 | 0.72 | 1.00 | 0.24 | 1.00 | 0.09 | 1.00 | 0.40 | 1.00 |
|  | rs12964264 | 53325504 | 0.73 | 1.00 | 0.78 | 1.00 | 0.92 | 1.00 | 0.71 | 1.00 |
|  | rs12965620 | 53325987 | 0.70 | 1.00 | 0.78 | 1.00 | 0.48 | 1.00 | 0.07 | 1.00 |
|  | rs12458731 | 53326575 | 0.65 | 1.00 | 0.24 | 1.00 | 0.13 | 1.00 | 0.26 | 1.00 |
|  | rs607664 | 53326656 | 0.65 | 1.00 | 0.27 | 1.00 | 0.13 | 1.00 | 0.26 | 1.00 |
|  | rs899103 | 53327370 | 0.65 | 1.00 | 0.79 | 1.00 | 0.13 | 1.00 | 0.26 | 1.00 |
| ZNF804A | rs359878 | 185438949 | 0.70 | 1.00 | 0.71 | 1.00 | 0.66 | 1.00 | 0.44 | 1.00 |
|  | rs55781342 | 185439065 | 0.95 | 1.00 | 0.78 | 1.00 | 0.87 | 1.00 | 0.89 | 1.00 |
|  | rs359879 | 185439121 | 0.35 | 1.00 | 0.78 | 1.00 | 0.85 | 1.00 | 0.05 | 1.00 |
|  | rs62174940 | 185439307 | 0.72 | 1.00 | 0.79 | 1.00 | 0.63 | 1.00 | 0.88 | 1.00 |
|  | rs359880 | 185439359 | 0.37 | 1.00 | 0.32 | 1.00 | 0.74 | 1.00 | 0.47 | 1.00 |
|  | rs11691444 | 185439634 | 0.72 | 1.00 | 0.45 | 1.00 | 0.63 | 1.00 | 0.88 | 1.00 |
|  | rs9678515 | 185440309 | 0.58 | 1.00 | 0.78 | 1.00 | 0.69 | 1.00 | 0.93 | 1.00 |
|  | rs139772309 | 185440512 | 0.72 | 1.00 | 0.96 | 1.00 | 0.74 | 1.00 | 0.93 | 1.00 |
|  | rs112478973 | 185440728 | 0.72 | 1.00 | 0.24 | 1.00 | 0.74 | 1.00 | 0.49 | 1.00 |
|  | rs72625292 | 185441009 | 0.72 | 1.00 | 0.99 | 1.00 | 0.63 | 1.00 | 0.88 | 1.00 |
|  | rs35574370 | 185442031 | 0.86 | 1.00 | 0.99 | 1.00 | 0.80 | 1.00 | 0.19 | 1.00 |
|  | rs373528 | 185442769 | 0.35 | 1.00 | 0.79 | 1.00 | 0.85 | 1.00 | 0.05 | 1.00 |
|  | rs79754244 | 185442770 | 0.20 | 1.00 | 0.30 | 1.00 | 0.70 | 1.00 | 0.07 | 1.00 |
|  | rs367464 | 185442920 | 0.37 | 1.00 | 0.78 | 1.00 | 0.72 | 1.00 | 0.53 | 1.00 |
|  | rs414068 | 185443205 | 0.37 | 1.00 | 0.79 | 1.00 | 0.72 | 1.00 | 0.53 | 1.00 |
|  | rs7586860 | 185443672 | 0.95 | 1.00 | 0.45 | 1.00 | 0.87 | 1.00 | 0.89 | 1.00 |
|  | rs75786365 | 185444073 | 0.18 | 1.00 | 0.79 | 1.00 | 0.75 | 1.00 | 0.13 | 1.00 |
|  | rs10198713 | 185444506 | 0.58 | 1.00 | 0.05 | 1.00 | 0.69 | 1.00 | 0.93 | 1.00 |
|  | rs12618211 | 185445333 | 0.72 | 1.00 | 0.25 | 1.00 | 0.63 | 1.00 | 0.88 | 1.00 |
|  | rs387374 | 185445876 | 0.35 | 1.00 | 0.77 | 1.00 | 0.85 | 1.00 | 0.05 | 1.00 |
|  | rs410607 | 185445880 | 0.35 | 1.00 | 0.79 | 1.00 | 0.85 | 1.00 | 0.05 | 1.00 |
|  | rs192444460 | 185445949 | 0.62 | 1.00 | 0.95 | 1.00 | 0.82 | 1.00 | 0.63 | 1.00 |
|  | rs974729 | 185446205 | 0.60 | 1.00 | 0.78 | 1.00 | 0.69 | 1.00 | 0.95 | 1.00 |
|  | rs34651316 | 185446748 | 0.86 | 1.00 | 0.45 | 1.00 | 0.76 | 1.00 | 0.19 | 1.00 |
|  | rs10931148 | 185446749 | 0.84 | 1.00 | 0.79 | 1.00 | 0.62 | 1.00 | 0.18 | 1.00 |
|  | rs1365718 | 185447148 | 0.84 | 1.00 | 0.79 | 1.00 | 0.62 | 1.00 | 0.18 | 1.00 |
|  | rs359899 | 185448231 | 0.41 | 1.00 | 0.95 | 1.00 | 0.68 | 1.00 | 0.52 | 1.00 |
|  | rs1593664 | 185449963 | 0.99 | 1.00 | 0.95 | 1.00 | 0.92 | 1.00 | 0.91 | 1.00 |
|  | 2:185450046:D | 185450046 | 0.41 | 1.00 | 0.96 | 1.00 | 0.68 | 1.00 | 0.52 | 1.00 |
|  | rs1834732 | 185450208 | 0.65 | 1.00 | 0.79 | 1.00 | 0.65 | 1.00 | 0.94 | 1.00 |
|  | rs13023840 | 185450305 | 0.99 | 1.00 | 0.34 | 1.00 | 0.92 | 1.00 | 0.91 | 1.00 |
|  | rs76926829 | 185450657 | 0.84 | 1.00 | 0.78 | 1.00 | 0.62 | 1.00 | 0.18 | 1.00 |
|  | rs35759594 | 185450792 | 0.86 | 1.00 | 0.33 | 1.00 | 0.76 | 1.00 | 0.19 | 1.00 |
|  | rs79264687 | 185451093 | 0.52 | 1.00 | 0.79 | 1.00 | 0.94 | 1.00 | 0.29 | 1.00 |
|  | rs74928904 | 185451515 | 0.84 | 1.00 | 0.88 | 1.00 | 0.62 | 1.00 | 0.18 | 1.00 |
|  | rs359900 | 185451906 | 0.35 | 1.00 | 0.79 | 1.00 | 0.85 | 1.00 | 0.05 | 1.00 |
|  | rs10469726 | 185452799 | 0.72 | 1.00 | 0.99 | 1.00 | 0.76 | 1.00 | 1.00 | 1.00 |
|  | rs1021042 | 185453158 | 0.60 | 1.00 | 0.61 | 1.00 | 0.76 | 1.00 | 0.95 | 1.00 |
|  | rs13007925 | 185453159 | 1.00 | 1.00 | 0.40 | 1.00 | 0.84 | 1.00 | 0.28 | 1.00 |
|  | rs1021041 | 185454461 | 0.61 | 1.00 | 0.13 | 1.00 | 0.76 | 1.00 | 1.00 | 1.00 |
|  | rs359902 | 185454836 | 0.43 | 1.00 | 0.79 | 1.00 | 0.68 | 1.00 | 0.93 | 1.00 |
|  | rs74661316 | 185455349 | 0.72 | 1.00 | 0.79 | 1.00 | 0.79 | 1.00 | 0.50 | 1.00 |
|  | rs10432495 | 185455455 | 0.53 | 1.00 | 0.53 | 1.00 | 0.55 | 1.00 | 0.07 | 1.00 |
|  | rs1019706 | 185455629 | 0.52 | 1.00 | 0.32 | 1.00 | 0.39 | 1.00 | 0.78 | 1.00 |
|  | rs2052794 | 185455751 | 0.85 | 1.00 | 0.79 | 1.00 | 0.42 | 1.00 | 0.07 | 1.00 |
|  | rs12470178 | 185456133 | 0.29 | 1.00 | 0.84 | 1.00 | 0.56 | 1.00 | 0.63 | 1.00 |
|  | rs75747525 | 185456897 | 0.74 | 1.00 | 0.93 | 1.00 | 0.99 | 1.00 | 0.37 | 1.00 |
|  | rs12992164 | 185457788 | 0.29 | 1.00 | 0.81 | 1.00 | 0.56 | 1.00 | 0.63 | 1.00 |
|  | rs16826057 | 185458209 | 0.81 | 1.00 | 0.69 | 1.00 | 0.42 | 1.00 | 0.06 | 1.00 |
|  | rs4366862 | 185458441 | 0.81 | 1.00 | 0.69 | 1.00 | 0.42 | 1.00 | 0.06 | 1.00 |
|  | rs17508485 | 185458975 | 0.99 | 1.00 | 0.69 | 1.00 | 0.12 | 1.00 | 0.33 | 1.00 |
|  | rs1978573 | 185459544 | 0.52 | 1.00 | 0.81 | 1.00 | 0.41 | 1.00 | 0.80 | 1.00 |
|  | rs11888068 | 185460295 | 0.50 | 1.00 | 0.37 | 1.00 | 0.41 | 1.00 | 0.83 | 1.00 |
|  | rs17617267 | 185460397 | 0.29 | 1.00 | 0.69 | 1.00 | 0.56 | 1.00 | 0.63 | 1.00 |
|  | rs73041376 | 185460423 | 0.81 | 1.00 | 0.69 | 1.00 | 0.42 | 1.00 | 0.06 | 1.00 |
|  | rs13026173 | 185460842 | 0.29 | 1.00 | 0.95 | 1.00 | 0.56 | 1.00 | 0.63 | 1.00 |
|  | rs62176168 | 185461133 | 0.14 | 1.00 | 0.69 | 1.00 | 0.74 | 1.00 | 0.30 | 1.00 |
|  | rs17617285 | 185461136 | 0.29 | 1.00 | 0.40 | 1.00 | 0.56 | 1.00 | 0.63 | 1.00 |
|  | rs10497655 | 185462041 | 0.57 | 1.00 | 0.79 | 1.00 | 0.70 | 1.00 | 0.84 | 1.00 |
|  | rs34714481 | 185462469 | 0.30 | 1.00 | 0.79 | 1.00 | 0.98 | 1.00 | 0.73 | 1.00 |
|  | rs2279266 | 185463120 | 0.33 | 1.00 | 0.45 | 1.00 | 0.76 | 1.00 | 0.51 | 1.00 |
|  | rs359895 | 185463185 | 0.93 | 1.00 | 0.35 | 1.00 | 0.83 | 1.00 | 0.77 | 1.00 |
|  | rs139485533 | 185463376 | 0.52 | 1.00 | 0.97 | 1.00 | 0.12 | 1.00 | 0.57 | 1.00 |
|  | rs73041379 | 185463535 | 0.74 | 1.00 | 0.79 | 1.00 | 0.54 | 1.00 | 0.03 | 1.00 |
|  | rs12693380 | 185466008 | 0.81 | 1.00 | 0.43 | 1.00 | 0.10 | 1.00 | 0.12 | 1.00 |
|  | rs62176169 | 185466432 | 0.42 | 1.00 | 0.11 | 1.00 | 0.92 | 1.00 | 0.26 | 1.00 |
|  | rs10203158 | 185466837 | 0.77 | 1.00 | 0.46 | 1.00 | 0.96 | 1.00 | 0.72 | 1.00 |
|  | rs138786316 | 185466841 | 0.45 | 1.00 | 0.35 | 1.00 | 0.85 | 1.00 | 0.06 | 1.00 |
|  | rs12693381 | 185466909 | 0.77 | 1.00 | 0.36 | 1.00 | 0.96 | 1.00 | 0.72 | 1.00 |
|  | rs60269985 | 185467609 | 0.77 | 1.00 | 0.48 | 1.00 | 0.25 | 1.00 | 0.05 | 1.00 |
|  | rs12693382 | 185467661 | 0.81 | 1.00 | 0.69 | 1.00 | 0.10 | 1.00 | 0.12 | 1.00 |
|  | rs12693383 | 185468863 | 0.91 | 1.00 | 0.11 | 1.00 | 0.95 | 1.00 | 0.29 | 1.00 |
|  | rs76878347 | 185469031 | 0.80 | 1.00 | 0.32 | 1.00 | 0.77 | 1.00 | 0.97 | 1.00 |
|  | rs435822 | 185469302 | 0.89 | 1.00 | 0.43 | 1.00 | 0.82 | 1.00 | 0.62 | 1.00 |
|  | rs75482695 | 185469866 | 0.15 | 1.00 | 0.75 | 1.00 | 0.60 | 1.00 | 0.32 | 1.00 |
|  | rs430040 | 185470013 | 0.89 | 1.00 | 0.06 | 1.00 | 0.82 | 1.00 | 0.62 | 1.00 |
|  | rs10931149 | 185471113 | 0.32 | 1.00 | 0.73 | 1.00 | 0.87 | 1.00 | 0.52 | 1.00 |
|  | rs72897732 | 185471168 | 0.73 | 1.00 | 0.47 | 1.00 | 0.35 | 1.00 | 0.59 | 1.00 |
|  | rs80078811 | 185471169 | 0.92 | 1.00 | 0.15 | 1.00 | 0.40 | 1.00 | 0.42 | 1.00 |
|  | rs72897733 | 185471738 | 0.73 | 1.00 | 0.15 | 1.00 | 0.35 | 1.00 | 0.59 | 1.00 |
|  | rs1014959 | 185472113 | 0.77 | 1.00 | 0.47 | 1.00 | 0.23 | 1.00 | 0.05 | 1.00 |
|  | rs72897735 | 185473761 | 0.62 | 1.00 | 0.45 | 1.00 | 0.98 | 1.00 | 0.10 | 1.00 |
|  | rs114105057 | 185474546 | 0.44 | 1.00 | 0.01 | 1.00 | 0.87 | 1.00 | 0.09 | 1.00 |
|  | rs72897737 | 185474789 | 0.73 | 1.00 | 0.47 | 1.00 | 0.35 | 1.00 | 0.59 | 1.00 |
|  | rs403115 | 185475579 | 0.93 | 1.00 | 0.49 | 1.00 | 0.62 | 1.00 | 0.95 | 1.00 |
|  | 2:185476002:D | 185476002 | 0.90 | 1.00 | 0.71 | 1.00 | 0.17 | 1.00 | 0.16 | 1.00 |
|  | rs722384 | 185476009 | 0.30 | 1.00 | 0.83 | 1.00 | 0.49 | 1.00 | 0.41 | 1.00 |
|  | rs722385 | 185476050 | 0.65 | 1.00 | 0.28 | 1.00 | 0.31 | 1.00 | 0.54 | 1.00 |
|  | 2:185476420:I | 185476420 | 0.73 | 1.00 | 0.63 | 1.00 | 0.30 | 1.00 | 0.05 | 1.00 |
|  | rs191554906 | 185477180 | 0.69 | 1.00 | 0.71 | 1.00 | 0.20 | 1.00 | 0.15 | 1.00 |
|  | rs13393273 | 185477677 | 0.84 | 1.00 | 0.37 | 1.00 | 0.73 | 1.00 | 0.53 | 1.00 |
|  | rs12693384 | 185478036 | 0.84 | 1.00 | 0.71 | 1.00 | 0.73 | 1.00 | 0.53 | 1.00 |
|  | rs148516959 | 185478566 | 0.67 | 1.00 | 0.69 | 1.00 | 0.29 | 1.00 | 0.56 | 1.00 |
|  | rs10202700 | 185479343 | 0.88 | 1.00 | 0.01 | 1.00 | 0.08 | 1.00 | 0.12 | 1.00 |
|  | rs73041394 | 185479385 | 0.73 | 1.00 | 0.23 | 1.00 | 0.30 | 1.00 | 0.05 | 1.00 |
|  | rs35653021 | 185480210 | 0.24 | 1.00 | 0.71 | 1.00 | 0.42 | 1.00 | 0.58 | 1.00 |
|  | rs1427150 | 185480633 | 0.73 | 1.00 | 0.37 | 1.00 | 0.30 | 1.00 | 0.05 | 1.00 |
|  | rs6715977 | 185481215 | 0.88 | 1.00 | 0.35 | 1.00 | 0.08 | 1.00 | 0.12 | 1.00 |
|  | rs17617356 | 185482155 | 0.33 | 1.00 | 0.50 | 1.00 | 0.97 | 1.00 | 0.57 | 1.00 |
|  | rs17508595 | 185483108 | 0.77 | 1.00 | 0.26 | 1.00 | 0.27 | 1.00 | 0.05 | 1.00 |
|  | rs11900132 | 185483812 | 0.79 | 1.00 | 0.50 | 1.00 | 0.27 | 1.00 | 0.08 | 1.00 |
|  | rs74942702 | 185483816 | 0.54 | 1.00 | 0.50 | 1.00 | 0.75 | 1.00 | 0.21 | 1.00 |
|  | rs359882 | 185483858 | 0.88 | 1.00 | 0.62 | 1.00 | 0.62 | 1.00 | 0.90 | 1.00 |
|  | rs17617408 | 185484811 | 0.38 | 1.00 | 0.71 | 1.00 | 0.92 | 1.00 | 0.64 | 1.00 |
|  | rs117107763 | 185484956 | 0.71 | 1.00 | 0.37 | 1.00 | 0.30 | 1.00 | 0.82 | 1.00 |
|  | 2:185485025:D | 185485025 | 0.61 | 1.00 | 0.48 | 1.00 | 0.33 | 1.00 | 0.64 | 1.00 |
|  | rs74954231 | 185485836 | 0.07 | 1.00 | 0.09 | 1.00 | 0.45 | 1.00 | 0.14 | 1.00 |
|  | rs359884 | 185486334 | 0.95 | 1.00 | 0.50 | 1.00 | 0.59 | 1.00 | 0.76 | 1.00 |
|  | 2:185487118:D | 185487118 | 0.95 | 1.00 | 0.69 | 1.00 | 0.61 | 1.00 | 0.76 | 1.00 |
|  | rs359885 | 185487473 | 0.95 | 1.00 | 0.83 | 1.00 | 0.59 | 1.00 | 0.76 | 1.00 |
|  | rs17508623 | 185487715 | 0.66 | 1.00 | 0.56 | 1.00 | 0.31 | 1.00 | 0.63 | 1.00 |
|  | rs72897742 | 185488676 | 0.66 | 1.00 | 0.06 | 1.00 | 0.31 | 1.00 | 0.63 | 1.00 |
|  | rs12613195 | 185489221 | 0.61 | 1.00 | 0.10 | 1.00 | 0.49 | 1.00 | 0.61 | 1.00 |
|  | rs359886 | 185489407 | 0.95 | 1.00 | 0.50 | 1.00 | 0.59 | 1.00 | 0.76 | 1.00 |
|  | rs17617468 | 185489430 | 0.38 | 1.00 | 0.50 | 1.00 | 0.92 | 1.00 | 0.64 | 1.00 |
|  | rs17508630 | 185490209 | 0.02 | 0.90 | 0.61 | 0.88 | 0.23 | 1.00 | 0.09 | 1.00 |
|  | rs17508644 | 185491014 | 0.66 | 1.00 | 0.50 | 1.00 | 0.31 | 1.00 | 0.63 | 1.00 |
|  | rs17430586 | 185491232 | 0.66 | 1.00 | 0.50 | 1.00 | 0.31 | 1.00 | 0.63 | 1.00 |
|  | rs17430600 | 185491437 | 0.81 | 1.00 | 0.36 | 1.00 | 0.81 | 1.00 | 0.43 | 1.00 |
|  | rs17508671 | 185491847 | 0.66 | 1.00 | 0.50 | 1.00 | 0.31 | 1.00 | 0.63 | 1.00 |
|  | rs17430621 | 185492469 | 0.66 | 1.00 | 0.31 | 1.00 | 0.31 | 1.00 | 0.63 | 1.00 |
|  | 2:185493993:D | 185493993 | 0.93 | 1.00 | 0.69 | 1.00 | 0.96 | 1.00 | 0.32 | 1.00 |
|  | rs80243100 | 185496411 | 0.49 | 1.00 | 0.61 | 1.00 | 0.68 | 1.00 | 0.76 | 1.00 |
|  | rs17430628 | 185496490 | 0.66 | 1.00 | 0.39 | 1.00 | 0.31 | 1.00 | 0.63 | 1.00 |
|  | rs17430635 | 185496638 | 0.68 | 1.00 | 0.36 | 1.00 | 0.32 | 1.00 | 0.62 | 1.00 |
|  | 2:185496873:D | 185496873 | 0.86 | 1.00 | 0.95 | 1.00 | 0.95 | 1.00 | 0.59 | 1.00 |
|  | rs72897758 | 185497170 | 0.66 | 1.00 | 0.69 | 1.00 | 0.31 | 1.00 | 0.63 | 1.00 |
|  | rs359891 | 185497226 | 0.95 | 1.00 | 0.67 | 1.00 | 0.59 | 1.00 | 0.76 | 1.00 |
|  | rs359892 | 185497327 | 0.77 | 1.00 | 0.69 | 1.00 | 0.78 | 1.00 | 0.48 | 1.00 |
|  | rs75685699 | 185497804 | 0.72 | 1.00 | 0.87 | 1.00 | 0.24 | 1.00 | 0.03 | 1.00 |
|  | rs12990519 | 185497969 | 0.81 | 1.00 | 0.67 | 1.00 | 0.12 | 1.00 | 0.40 | 1.00 |
|  | rs6434092 | 185498003 | 0.53 | 1.00 | 0.50 | 1.00 | 0.86 | 1.00 | 0.80 | 1.00 |
|  | rs190306973 | 185498111 | 0.93 | 1.00 | 0.21 | 1.00 | 0.79 | 1.00 | 0.46 | 1.00 |
|  | rs7557843 | 185498238 | 0.61 | 1.00 | 0.69 | 1.00 | 0.49 | 1.00 | 0.61 | 1.00 |
|  | rs72625293 | 185499138 | 0.34 | 1.00 | 0.50 | 1.00 | 0.49 | 1.00 | 0.44 | 1.00 |
|  | rs62176172 | 185499482 | 0.61 | 1.00 | 0.69 | 1.00 | 0.49 | 1.00 | 0.61 | 1.00 |
|  | rs62176173 | 185499540 | 0.68 | 1.00 | 0.67 | 1.00 | 0.51 | 1.00 | 0.68 | 1.00 |
|  | rs35068933 | 185499580 | 0.90 | 1.00 | 0.61 | 1.00 | 0.08 | 1.00 | 0.12 | 1.00 |
|  | rs7591757 | 185500146 | 0.90 | 1.00 | 0.21 | 1.00 | 0.08 | 1.00 | 0.12 | 1.00 |
|  | rs6434094 | 185500162 | 0.95 | 1.00 | 0.86 | 1.00 | 0.59 | 1.00 | 0.76 | 1.00 |
|  | rs35431480 | 185500279 | 0.90 | 1.00 | 0.33 | 1.00 | 0.08 | 1.00 | 0.12 | 1.00 |
|  | rs2054549 | 185501950 | 0.95 | 1.00 | 0.33 | 1.00 | 0.59 | 1.00 | 0.76 | 1.00 |
|  | rs2054548 | 185502024 | 0.95 | 1.00 | 0.67 | 1.00 | 0.59 | 1.00 | 0.76 | 1.00 |
|  | rs7600673 | 185503912 | 0.90 | 1.00 | 0.33 | 1.00 | 0.08 | 1.00 | 0.12 | 1.00 |
|  | rs146041840 | 185504506 | 0.99 | 1.00 | 0.53 | 1.00 | 0.07 | 1.00 | 0.17 | 1.00 |
|  | rs72897775 | 185504805 | 0.63 | 1.00 | 0.15 | 1.00 | 0.30 | 1.00 | 0.62 | 1.00 |
|  | 2:185504949:D | 185504949 | 0.65 | 1.00 | 0.33 | 1.00 | 0.99 | 1.00 | 0.81 | 1.00 |
|  | rs7564347 | 185505380 | 0.95 | 1.00 | 0.56 | 1.00 | 0.59 | 1.00 | 0.76 | 1.00 |
|  | rs1480478 | 185505556 | 0.95 | 1.00 | 0.69 | 1.00 | 0.59 | 1.00 | 0.76 | 1.00 |
|  | rs72897782 | 185506728 | 0.63 | 1.00 | 0.69 | 1.00 | 0.30 | 1.00 | 0.62 | 1.00 |
|  | rs11890128 | 185506877 | 0.70 | 1.00 | 0.67 | 1.00 | 0.25 | 1.00 | 0.03 | 0.99 |
|  | rs35230759 | 185506949 | 0.38 | 1.00 | 0.87 | 1.00 | 0.92 | 1.00 | 0.64 | 1.00 |
|  | rs12693385 | 185507229 | 0.95 | 1.00 | 0.96 | 1.00 | 0.59 | 1.00 | 0.76 | 1.00 |
|  | rs1021043 | 185507421 | 0.80 | 1.00 | 0.33 | 1.00 | 0.80 | 1.00 | 0.42 | 1.00 |
|  | rs62176207 | 185508271 | 0.90 | 1.00 | 0.93 | 1.00 | 0.08 | 1.00 | 0.12 | 1.00 |
|  | rs78131089 | 185508470 | 0.70 | 1.00 | 0.50 | 1.00 | 0.62 | 1.00 | 0.45 | 1.00 |
|  | rs57159364 | 185508496 | 0.61 | 1.00 | 0.05 | 1.00 | 0.49 | 1.00 | 0.61 | 1.00 |
|  | rs72897790 | 185508536 | 0.63 | 1.00 | 0.33 | 1.00 | 0.30 | 1.00 | 0.62 | 1.00 |
|  | rs76627958 | 185508751 | 0.72 | 1.00 | 0.15 | 1.00 | 0.21 | 1.00 | 0.03 | 0.99 |
|  | rs10198049 | 185508842 | 0.95 | 1.00 | 0.95 | 1.00 | 0.59 | 1.00 | 0.76 | 1.00 |
|  | rs72897793 | 185509934 | 0.63 | 1.00 | 0.33 | 1.00 | 0.30 | 1.00 | 0.62 | 1.00 |
|  | rs994653 | 185509985 | 0.38 | 1.00 | 0.50 | 1.00 | 0.92 | 1.00 | 0.64 | 1.00 |
|  | rs72897795 | 185510280 | 0.63 | 1.00 | 0.33 | 1.00 | 0.30 | 1.00 | 0.62 | 1.00 |
|  | rs111876364 | 185511230 | 0.11 | 1.00 | 0.67 | 1.00 | 0.29 | 1.00 | 0.88 | 1.00 |
|  | rs34934597 | 185511577 | 0.96 | 1.00 | 0.33 | 1.00 | 0.07 | 1.00 | 0.10 | 1.00 |
|  | rs13021843 | 185512838 | 0.93 | 1.00 | 0.67 | 1.00 | 0.57 | 1.00 | 0.80 | 1.00 |
|  | rs72897799 | 185513142 | 0.63 | 1.00 | 0.33 | 1.00 | 0.30 | 1.00 | 0.62 | 1.00 |
|  | 2:185513231:D | 185513231 | 0.99 | 1.00 | 0.13 | 1.00 | 0.90 | 1.00 | 0.50 | 1.00 |
|  | rs6434095 | 185513327 | 0.95 | 1.00 | 0.50 | 1.00 | 0.59 | 1.00 | 0.76 | 1.00 |
|  | rs12469299 | 185513496 | 0.38 | 1.00 | 0.49 | 1.00 | 0.92 | 1.00 | 0.64 | 1.00 |
|  | rs72897802 | 185514314 | 0.63 | 1.00 | 0.50 | 1.00 | 0.30 | 1.00 | 0.62 | 1.00 |
|  | rs72899904 | 185514927 | 0.63 | 1.00 | 0.33 | 1.00 | 0.30 | 1.00 | 0.62 | 1.00 |
|  | rs7608284 | 185514937 | 0.76 | 1.00 | 0.33 | 1.00 | 0.77 | 1.00 | 0.47 | 1.00 |
|  | rs7608194 | 185514989 | 0.76 | 1.00 | 0.33 | 1.00 | 0.77 | 1.00 | 0.47 | 1.00 |
|  | 2:185515421:I | 185515421 | 0.52 | 1.00 | 1.00 | 1.00 | 0.36 | 1.00 | 0.60 | 1.00 |
|  | rs72899906 | 185515793 | 0.63 | 1.00 | 0.50 | 1.00 | 0.30 | 1.00 | 0.62 | 1.00 |
|  | rs150670123 | 185516123 | 0.71 | 1.00 | 0.63 | 1.00 | 0.30 | 1.00 | 0.82 | 1.00 |
|  | rs6434096 | 185516724 | 0.95 | 1.00 | 0.69 | 1.00 | 0.59 | 1.00 | 0.76 | 1.00 |
|  | rs55937523 | 185516968 | 0.67 | 1.00 | 0.11 | 1.00 | 0.31 | 1.00 | 0.19 | 1.00 |
|  | rs139561322 | 185517482 | 0.64 | 1.00 | 0.23 | 1.00 | 0.30 | 1.00 | 0.65 | 1.00 |
|  | rs10197925 | 185517836 | 0.70 | 1.00 | 0.19 | 1.00 | 0.79 | 1.00 | 0.56 | 1.00 |
|  | rs990844 | 185519085 | 0.64 | 1.00 | 0.50 | 1.00 | 0.30 | 1.00 | 0.65 | 1.00 |
|  | rs62176209 | 185519428 | 0.61 | 1.00 | 0.50 | 1.00 | 0.49 | 1.00 | 0.61 | 1.00 |
|  | rs143273342 | 185519525 | 0.59 | 1.00 | 0.67 | 1.00 | 0.92 | 1.00 | 0.57 | 1.00 |
|  | rs73043234 | 185519645 | 0.78 | 1.00 | 0.33 | 1.00 | 0.30 | 1.00 | 0.04 | 1.00 |
|  | rs76629295 | 185520300 | 0.53 | 1.00 | 0.10 | 1.00 | 0.11 | 1.00 | 0.25 | 1.00 |
|  | rs55764167 | 185520787 | 0.95 | 1.00 | 0.65 | 1.00 | 0.61 | 1.00 | 0.73 | 1.00 |
|  | rs72899915 | 185521066 | 0.64 | 1.00 | 0.65 | 1.00 | 0.30 | 1.00 | 0.65 | 1.00 |
|  | rs73043238 | 185522052 | 0.84 | 1.00 | 0.33 | 1.00 | 0.44 | 1.00 | 0.04 | 1.00 |
|  | rs6738793 | 185522156 | 0.99 | 1.00 | 0.65 | 1.00 | 0.11 | 1.00 | 0.14 | 1.00 |
|  | rs72899918 | 185523458 | 0.64 | 1.00 | 0.65 | 1.00 | 0.30 | 1.00 | 0.65 | 1.00 |
|  | rs148993723 | 185523820 | 0.80 | 1.00 | 0.65 | 1.00 | 0.50 | 1.00 | 0.31 | 1.00 |
|  | rs35979599 | 185523868 | 0.41 | 1.00 | 0.65 | 1.00 | 0.35 | 1.00 | 0.82 | 1.00 |
|  | rs17508706 | 185524591 | 0.66 | 1.00 | 0.67 | 1.00 | 0.34 | 1.00 | 0.63 | 1.00 |
|  | rs2369593 | 185524642 | 0.53 | 1.00 | 0.62 | 1.00 | 0.86 | 1.00 | 0.80 | 1.00 |
|  | rs899845 | 185525988 | 0.53 | 1.00 | 0.70 | 1.00 | 0.86 | 1.00 | 0.80 | 1.00 |
|  | rs899846 | 185526003 | 0.53 | 1.00 | 0.50 | 1.00 | 0.86 | 1.00 | 0.80 | 1.00 |
|  | rs72899932 | 185526272 | 0.66 | 1.00 | 0.33 | 1.00 | 0.34 | 1.00 | 0.63 | 1.00 |
|  | rs60170726 | 185527505 | 0.99 | 1.00 | 0.51 | 1.00 | 0.14 | 1.00 | 0.09 | 1.00 |
|  | rs7581009 | 185527631 | 0.53 | 1.00 | 0.59 | 1.00 | 0.86 | 1.00 | 0.80 | 1.00 |
|  | rs1480479 | 185528929 | 0.53 | 1.00 | 0.33 | 1.00 | 0.86 | 1.00 | 0.80 | 1.00 |
|  | rs147725178 | 185529344 | 0.99 | 1.00 | 0.62 | 1.00 | 0.90 | 1.00 | 0.50 | 1.00 |
|  | rs2035320 | 185529538 | 0.53 | 1.00 | 0.33 | 1.00 | 0.86 | 1.00 | 0.80 | 1.00 |
|  | rs9288106 | 185529665 | 0.75 | 1.00 | 0.41 | 1.00 | 0.33 | 1.00 | 0.04 | 1.00 |
|  | rs9678203 | 185529712 | 0.95 | 1.00 | 0.69 | 1.00 | 0.61 | 1.00 | 0.73 | 1.00 |
|  | rs2035321 | 185529731 | 0.95 | 1.00 | 0.69 | 1.00 | 0.61 | 1.00 | 0.73 | 1.00 |
|  | rs72899936 | 185529923 | 0.87 | 1.00 | 0.33 | 1.00 | 0.16 | 1.00 | 0.76 | 1.00 |
|  | rs17508713 | 185530164 | 0.93 | 1.00 | 0.25 | 1.00 | 0.10 | 1.00 | 0.09 | 1.00 |
|  | rs13405270 | 185530269 | 0.77 | 1.00 | 0.69 | 1.00 | 0.75 | 1.00 | 0.43 | 1.00 |
|  | rs899847 | 185530491 | 0.98 | 1.00 | 0.32 | 1.00 | 0.64 | 1.00 | 0.74 | 1.00 |
|  | rs11891662 | 185531003 | 0.77 | 1.00 | 0.06 | 1.00 | 0.35 | 1.00 | 0.03 | 0.99 |
|  | rs115294081 | 185531148 | 0.07 | 1.00 | 0.69 | 1.00 | 0.78 | 1.00 | 0.12 | 1.00 |
|  | rs16826121 | 185532609 | 0.84 | 1.00 | 0.69 | 1.00 | 0.37 | 1.00 | 0.88 | 1.00 |
|  | rs72899938 | 185532849 | 0.85 | 1.00 | 0.45 | 1.00 | 0.20 | 1.00 | 0.49 | 1.00 |
|  | rs7609208 | 185532918 | 0.32 | 1.00 | 0.52 | 1.00 | 0.84 | 1.00 | 0.58 | 1.00 |
|  | 2:185533175:D | 185533175 | 0.82 | 1.00 | 0.33 | 1.00 | 0.15 | 1.00 | 0.34 | 1.00 |
|  | rs72899941 | 185533546 | 0.62 | 1.00 | 0.60 | 1.00 | 0.98 | 1.00 | 0.10 | 1.00 |
|  | rs7597593 | 185533580 | 0.45 | 1.00 | 0.75 | 1.00 | 0.11 | 1.00 | 0.10 | 1.00 |
|  | 2:185533714:D | 185533714 | 0.29 | 1.00 | 0.48 | 1.00 | 0.81 | 1.00 | 0.63 | 1.00 |
|  | rs72899945 | 185533973 | 0.89 | 1.00 | 0.06 | 1.00 | 0.14 | 1.00 | 0.42 | 1.00 |
|  | 2:185534495:I | 185534495 | 0.54 | 1.00 | 0.32 | 1.00 | 0.79 | 1.00 | 0.78 | 1.00 |
|  | rs6756881 | 185535322 | 0.09 | 1.00 | 0.51 | 1.00 | 0.40 | 1.00 | 0.20 | 1.00 |
|  | rs6742182 | 185535621 | 0.56 | 1.00 | 0.52 | 1.00 | 0.84 | 1.00 | 0.78 | 1.00 |
|  | rs17508767 | 185535807 | 0.95 | 1.00 | 0.52 | 1.00 | 0.20 | 1.00 | 0.32 | 1.00 |
|  | rs6760360 | 185535865 | 0.14 | 1.00 | 0.12 | 1.00 | 0.69 | 1.00 | 0.22 | 1.00 |
|  | rs74904241 | 185535866 | 0.14 | 1.00 | 0.91 | 1.00 | 0.69 | 1.00 | 0.22 | 1.00 |
|  | rs72899950 | 185536384 | 0.95 | 1.00 | 0.31 | 1.00 | 0.20 | 1.00 | 0.32 | 1.00 |
|  | 2:185537474:D | 185537474 | 0.38 | 1.00 | 0.52 | 1.00 | 0.92 | 1.00 | 0.64 | 1.00 |
|  | rs186502020 | 185537618 | 0.02 | 0.90 | 0.74 | 0.86 | 0.21 | 1.00 | 0.16 | 1.00 |
|  | rs72899952 | 185538154 | 0.95 | 1.00 | 0.52 | 1.00 | 0.20 | 1.00 | 0.32 | 1.00 |
|  | rs6728764 | 185538930 | 0.18 | 1.00 | 0.62 | 1.00 | 0.54 | 1.00 | 0.63 | 1.00 |
|  | 2:185539522:D | 185539522 | 0.52 | 1.00 | 0.74 | 1.00 | 0.81 | 1.00 | 0.74 | 1.00 |
|  | rs76869506 | 185540217 | 0.41 | 1.00 | 0.52 | 1.00 | 0.47 | 1.00 | 0.18 | 1.00 |
|  | rs62176213 | 185541423 | 0.09 | 1.00 | 0.33 | 1.00 | 0.67 | 1.00 | 0.11 | 1.00 |
|  | 2:185541783:D | 185541783 | 0.70 | 1.00 | 0.38 | 1.00 | 0.19 | 1.00 | 0.33 | 1.00 |
|  | rs11901640 | 185542928 | 0.96 | 1.00 | 0.30 | 1.00 | 0.58 | 1.00 | 0.38 | 1.00 |
|  | rs34713272 | 185542953 | 0.95 | 1.00 | 0.04 | 1.00 | 0.12 | 1.00 | 0.08 | 1.00 |
|  | rs10184629 | 185543913 | 0.99 | 1.00 | 0.35 | 1.00 | 0.55 | 1.00 | 0.55 | 1.00 |
|  | rs11892742 | 185544254 | 0.95 | 1.00 | 0.31 | 1.00 | 0.56 | 1.00 | 0.58 | 1.00 |
|  | rs146429929 | 185544980 | 0.02 | 0.90 | 0.59 | 0.86 | 0.21 | 1.00 | 0.16 | 1.00 |
|  | rs10188162 | 185545033 | 0.21 | 1.00 | 0.50 | 1.00 | 0.75 | 1.00 | 0.77 | 1.00 |
|  | rs143097614 | 185545188 | 0.92 | 1.00 | 0.50 | 1.00 | 0.64 | 1.00 | 0.84 | 1.00 |
|  | rs6755921 | 185545310 | 0.95 | 1.00 | 0.22 | 1.00 | 0.12 | 1.00 | 0.08 | 1.00 |
|  | rs2199882 | 185545844 | 0.96 | 1.00 | 0.35 | 1.00 | 0.10 | 1.00 | 0.16 | 1.00 |
|  | rs72899961 | 185547456 | 0.93 | 1.00 | 0.69 | 1.00 | 0.22 | 1.00 | 0.37 | 1.00 |
|  | rs1480481 | 185547462 | 0.23 | 1.00 | 0.88 | 1.00 | 0.78 | 1.00 | 0.77 | 1.00 |
|  | rs72899964 | 185547491 | 0.93 | 1.00 | 0.47 | 1.00 | 0.22 | 1.00 | 0.37 | 1.00 |
|  | rs72899966 | 185547750 | 0.93 | 1.00 | 0.83 | 1.00 | 0.22 | 1.00 | 0.37 | 1.00 |
|  | 2:185547764:D | 185547764 | 0.96 | 1.00 | 0.68 | 1.00 | 0.46 | 1.00 | 0.46 | 1.00 |
|  | rs6723680 | 185547962 | 0.96 | 1.00 | 0.08 | 1.00 | 0.58 | 1.00 | 0.38 | 1.00 |
|  | rs12693387 | 185548026 | 0.95 | 1.00 | 0.46 | 1.00 | 0.12 | 1.00 | 0.08 | 1.00 |
|  | rs1480482 | 185548223 | 0.10 | 1.00 | 0.35 | 1.00 | 0.34 | 1.00 | 0.07 | 1.00 |
|  | rs114030182 | 185548628 | 0.05 | 0.99 | 0.01 | 1.00 | 0.81 | 1.00 | 0.15 | 1.00 |
|  | rs72899969 | 185549119 | 0.93 | 1.00 | 0.74 | 1.00 | 0.22 | 1.00 | 0.37 | 1.00 |
|  | rs6718759 | 185550104 | 0.95 | 1.00 | 0.59 | 1.00 | 0.56 | 1.00 | 0.58 | 1.00 |
|  | rs76706177 | 185550243 | 0.49 | 1.00 | 0.47 | 1.00 | 0.57 | 1.00 | 0.88 | 1.00 |
|  | rs112990204 | 185550412 | 0.52 | 1.00 | 0.17 | 1.00 | 0.93 | 1.00 | 0.72 | 1.00 |
|  | rs55782979 | 185550441 | 0.11 | 1.00 | 0.46 | 1.00 | 0.31 | 1.00 | 0.16 | 1.00 |
|  | rs78880814 | 185550465 | 0.04 | 0.98 | 0.59 | 1.00 | 0.98 | 1.00 | 0.75 | 1.00 |
|  | rs72899975 | 185550610 | 0.93 | 1.00 | 0.58 | 1.00 | 0.22 | 1.00 | 0.37 | 1.00 |
|  | rs72899976 | 185550807 | 0.93 | 1.00 | 0.36 | 1.00 | 0.22 | 1.00 | 0.37 | 1.00 |
|  | rs138124326 | 185550873 | 0.59 | 1.00 | 0.41 | 1.00 | 0.92 | 1.00 | 0.57 | 1.00 |
|  | rs72899981 | 185551554 | 0.93 | 1.00 | 0.47 | 1.00 | 0.22 | 1.00 | 0.37 | 1.00 |
|  | rs72899983 | 185552032 | 0.93 | 1.00 | 0.41 | 1.00 | 0.22 | 1.00 | 0.37 | 1.00 |
|  | rs2127698 | 185552659 | 0.97 | 1.00 | 0.07 | 1.00 | 0.11 | 1.00 | 0.07 | 1.00 |
|  | rs72899986 | 185552977 | 0.93 | 1.00 | 0.74 | 1.00 | 0.22 | 1.00 | 0.37 | 1.00 |
|  | rs10197098 | 185553100 | 0.99 | 1.00 | 0.43 | 1.00 | 0.09 | 1.00 | 0.07 | 1.00 |
|  | rs10173509 | 185553260 | 0.94 | 1.00 | 0.36 | 1.00 | 0.57 | 1.00 | 0.36 | 1.00 |
|  | rs150724374 | 185553348 | 0.59 | 1.00 | 0.74 | 1.00 | 0.92 | 1.00 | 0.57 | 1.00 |
|  | rs76396036 | 185555448 | 0.53 | 1.00 | 0.74 | 1.00 | 0.56 | 1.00 | 0.51 | 1.00 |
|  | rs17508877 | 185556071 | 0.97 | 1.00 | 0.36 | 1.00 | 0.11 | 1.00 | 0.07 | 1.00 |
|  | 2:185556142:D | 185556142 | 0.97 | 1.00 | 0.59 | 1.00 | 0.97 | 1.00 | 0.17 | 1.00 |
|  | rs1038197 | 185557271 | 0.97 | 1.00 | 0.09 | 1.00 | 0.54 | 1.00 | 0.53 | 1.00 |
|  | rs1038198 | 185557343 | 0.91 | 1.00 | 0.42 | 1.00 | 0.58 | 1.00 | 0.56 | 1.00 |
|  | rs7588753 | 185557590 | 0.97 | 1.00 | 0.36 | 1.00 | 0.54 | 1.00 | 0.53 | 1.00 |
|  | rs191526210 | 185557618 | 0.38 | 1.00 | 0.14 | 1.00 | 0.38 | 1.00 | 0.19 | 1.00 |
|  | rs16826158 | 185558080 | 0.93 | 1.00 | 0.59 | 1.00 | 0.56 | 1.00 | 0.55 | 1.00 |
|  | rs17508905 | 185558405 | 0.93 | 1.00 | 0.74 | 1.00 | 0.22 | 1.00 | 0.37 | 1.00 |
|  | rs72899997 | 185558421 | 0.92 | 1.00 | 0.36 | 1.00 | 0.04 | 0.99 | 0.62 | 1.00 |
|  | rs12104591 | 185558816 | 0.97 | 1.00 | 0.74 | 1.00 | 0.54 | 1.00 | 0.53 | 1.00 |
|  | 2:185558936:D | 185558936 | 0.93 | 1.00 | 0.59 | 1.00 | 0.22 | 1.00 | 0.37 | 1.00 |
|  | rs4308093 | 185559043 | 0.97 | 1.00 | 0.30 | 1.00 | 0.54 | 1.00 | 0.53 | 1.00 |
|  | rs62176215 | 185560052 | 0.26 | 1.00 | 0.74 | 1.00 | 0.45 | 1.00 | 0.20 | 1.00 |
|  | 2:185561949:D | 185561949 | 0.99 | 1.00 | 0.59 | 1.00 | 0.41 | 1.00 | 0.32 | 1.00 |
|  | rs10186035 | 185562421 | 0.65 | 1.00 | 0.59 | 1.00 | 0.15 | 1.00 | 0.17 | 1.00 |
|  | rs147145079 | 185564447 | 0.65 | 1.00 | 0.36 | 1.00 | 0.75 | 1.00 | 0.50 | 1.00 |
|  | rs7568756 | 185564593 | 0.98 | 1.00 | 0.74 | 1.00 | 0.10 | 1.00 | 0.15 | 1.00 |
|  | rs7585738 | 185564834 | 0.98 | 1.00 | 0.77 | 1.00 | 0.10 | 1.00 | 0.15 | 1.00 |
|  | rs2199883 | 185565463 | 0.93 | 1.00 | 0.74 | 1.00 | 0.56 | 1.00 | 0.55 | 1.00 |
|  | rs16826169 | 185566077 | 0.98 | 1.00 | 0.06 | 1.00 | 0.10 | 1.00 | 0.15 | 1.00 |
|  | rs17430890 | 185566543 | 0.39 | 1.00 | 0.36 | 1.00 | 0.92 | 1.00 | 0.54 | 1.00 |
|  | rs76167860 | 185567029 | 0.69 | 1.00 | 0.36 | 1.00 | 0.06 | 1.00 | 0.23 | 1.00 |
|  | rs6730122 | 185567451 | 0.98 | 1.00 | 0.36 | 1.00 | 0.10 | 1.00 | 0.15 | 1.00 |
|  | rs35743160 | 185567763 | 0.24 | 1.00 | 0.59 | 1.00 | 0.55 | 1.00 | 0.76 | 1.00 |
|  | rs1383309 | 185567912 | 0.97 | 1.00 | 0.74 | 1.00 | 0.54 | 1.00 | 0.53 | 1.00 |
|  | rs7583953 | 185568639 | 0.97 | 1.00 | 0.42 | 1.00 | 0.54 | 1.00 | 0.53 | 1.00 |
|  | rs114702410 | 185569263 | 0.39 | 1.00 | 0.59 | 1.00 | 0.68 | 1.00 | 0.57 | 1.00 |
|  | rs75524004 | 185569518 | 0.38 | 1.00 | 0.74 | 1.00 | 0.38 | 1.00 | 0.19 | 1.00 |
|  | rs77194274 | 185569542 | 0.87 | 1.00 | 0.10 | 1.00 | 0.76 | 1.00 | 0.67 | 1.00 |
|  | rs34494944 | 185569891 | 0.98 | 1.00 | 0.68 | 1.00 | 0.10 | 1.00 | 0.15 | 1.00 |
|  | rs79300728 | 185570857 | 0.96 | 1.00 | 0.42 | 1.00 | 0.95 | 1.00 | 0.62 | 1.00 |
|  | rs17509003 | 185571361 | 0.93 | 1.00 | 0.59 | 1.00 | 0.22 | 1.00 | 0.37 | 1.00 |
|  | rs75558946 | 185572844 | 0.02 | 0.93 | 0.34 | 1.00 | 0.84 | 1.00 | 0.54 | 1.00 |
|  | rs10180958 | 185572847 | 0.98 | 1.00 | 0.71 | 1.00 | 0.10 | 1.00 | 0.15 | 1.00 |
|  | rs79896103 | 185573098 | 0.66 | 1.00 | 0.95 | 1.00 | 0.07 | 1.00 | 0.24 | 1.00 |
|  | rs193248456 | 185573162 | 0.97 | 1.00 | 0.68 | 1.00 | 0.97 | 1.00 | 0.17 | 1.00 |
|  | rs7586454 | 185574627 | 0.98 | 1.00 | 0.36 | 1.00 | 0.10 | 1.00 | 0.15 | 1.00 |
|  | 2:185574918:D | 185574918 | 0.93 | 1.00 | 0.74 | 1.00 | 0.22 | 1.00 | 0.37 | 1.00 |
|  | rs11894318 | 185575353 | 0.98 | 1.00 | 0.36 | 1.00 | 0.10 | 1.00 | 0.15 | 1.00 |
|  | rs11895541 | 185575587 | 0.93 | 1.00 | 0.36 | 1.00 | 0.56 | 1.00 | 0.55 | 1.00 |
|  | rs13389014 | 185577032 | 0.98 | 1.00 | 0.59 | 1.00 | 0.10 | 1.00 | 0.15 | 1.00 |
|  | rs13389241 | 185577236 | 0.93 | 1.00 | 0.37 | 1.00 | 0.56 | 1.00 | 0.55 | 1.00 |
|  | rs13414832 | 185577340 | 0.98 | 1.00 | 0.74 | 1.00 | 0.10 | 1.00 | 0.15 | 1.00 |
|  | rs75368853 | 185577558 | 0.02 | 0.94 | 0.68 | 1.00 | 0.65 | 1.00 | 0.47 | 1.00 |
|  | rs17430952 | 185578086 | 0.93 | 1.00 | 0.36 | 1.00 | 0.22 | 1.00 | 0.37 | 1.00 |
|  | rs17430973 | 185578344 | 0.90 | 1.00 | 0.74 | 1.00 | 0.20 | 1.00 | 0.28 | 1.00 |
|  | rs72901814 | 185578987 | 0.93 | 1.00 | 0.35 | 1.00 | 0.22 | 1.00 | 0.37 | 1.00 |
|  | rs1480477 | 185579871 | 0.98 | 1.00 | 0.21 | 1.00 | 0.10 | 1.00 | 0.15 | 1.00 |
|  | rs1480476 | 185579987 | 0.98 | 1.00 | 0.74 | 1.00 | 0.10 | 1.00 | 0.15 | 1.00 |
|  | rs13383910 | 185580597 | 0.98 | 1.00 | 0.37 | 1.00 | 0.10 | 1.00 | 0.15 | 1.00 |
|  | rs146228221 | 185581370 | 0.98 | 1.00 | 0.36 | 1.00 | 0.97 | 1.00 | 0.54 | 1.00 |
|  | rs72901817 | 185582336 | 0.93 | 1.00 | 0.74 | 1.00 | 0.22 | 1.00 | 0.37 | 1.00 |
|  | rs72901819 | 185582428 | 0.60 | 1.00 | 0.74 | 1.00 | 0.94 | 1.00 | 0.13 | 1.00 |
|  | rs6747844 | 185582551 | 0.97 | 1.00 | 0.99 | 1.00 | 0.54 | 1.00 | 0.53 | 1.00 |
|  | rs113747911 | 185582833 | 0.08 | 1.00 | 0.59 | 1.00 | 0.74 | 1.00 | 0.13 | 1.00 |
|  | 2:185582972:D | 185582972 | 0.12 | 1.00 | 0.38 | 1.00 | 0.95 | 1.00 | 0.82 | 1.00 |
|  | 2:185582976:D | 185582976 | 0.15 | 1.00 | 0.39 | 1.00 | 0.83 | 1.00 | 0.60 | 1.00 |
|  | rs72901820 | 185583504 | 0.93 | 1.00 | 0.74 | 1.00 | 0.22 | 1.00 | 0.37 | 1.00 |
|  | rs116676375 | 185584606 | 0.93 | 1.00 | 0.68 | 1.00 | 0.22 | 1.00 | 0.37 | 1.00 |
|  | rs1600578 | 185586382 | 0.93 | 1.00 | 0.74 | 1.00 | 0.55 | 1.00 | 0.56 | 1.00 |
|  | rs1600577 | 185586483 | 0.98 | 1.00 | 0.36 | 1.00 | 0.10 | 1.00 | 0.15 | 1.00 |
|  | rs17430987 | 185587494 | 0.05 | 0.99 | 0.36 | 1.00 | 0.89 | 1.00 | 0.16 | 1.00 |
|  | rs6742299 | 185587759 | 0.90 | 1.00 | 0.74 | 1.00 | 0.56 | 1.00 | 0.59 | 1.00 |
|  | rs7582887 | 185588807 | 0.90 | 1.00 | 0.74 | 1.00 | 0.56 | 1.00 | 0.59 | 1.00 |
|  | rs7569229 | 185589013 | 0.98 | 1.00 | 0.36 | 1.00 | 0.10 | 1.00 | 0.15 | 1.00 |
|  | 2:185589193:D | 185589193 | 0.90 | 1.00 | 0.33 | 1.00 | 0.56 | 1.00 | 0.59 | 1.00 |
|  | 2:185589295:I | 185589295 | 0.90 | 1.00 | 0.59 | 1.00 | 0.56 | 1.00 | 0.59 | 1.00 |
|  | rs7569699 | 185589397 | 0.90 | 1.00 | 0.90 | 1.00 | 0.56 | 1.00 | 0.59 | 1.00 |
|  | rs1383308 | 185590264 | 0.90 | 1.00 | 0.36 | 1.00 | 0.56 | 1.00 | 0.59 | 1.00 |
|  | rs16826178 | 185591496 | 0.93 | 1.00 | 0.74 | 1.00 | 0.55 | 1.00 | 0.56 | 1.00 |
|  | rs34202603 | 185592151 | 0.80 | 1.00 | 0.59 | 1.00 | 0.24 | 1.00 | 0.31 | 1.00 |
|  | 2:185592842:I | 185592842 | 1.00 | 1.00 | 0.36 | 1.00 | 0.54 | 1.00 | 0.37 | 1.00 |
|  | 2:185592845:I | 185592845 | 0.83 | 1.00 | 0.74 | 1.00 | 0.42 | 1.00 | 0.39 | 1.00 |
|  | rs1383307 | 185593612 | 0.98 | 1.00 | 0.74 | 1.00 | 0.10 | 1.00 | 0.15 | 1.00 |
|  | rs79895553 | 185593635 | 0.30 | 1.00 | 0.36 | 1.00 | 0.77 | 1.00 | 0.82 | 1.00 |
|  | rs7568655 | 185594637 | 0.85 | 1.00 | 0.58 | 1.00 | 0.52 | 1.00 | 0.60 | 1.00 |
|  | rs7568843 | 185594655 | 0.98 | 1.00 | 0.36 | 1.00 | 0.10 | 1.00 | 0.15 | 1.00 |
|  | 2:185595059:D | 185595059 | 0.80 | 1.00 | 0.74 | 1.00 | 0.24 | 1.00 | 0.31 | 1.00 |
|  | rs6725125 | 185595844 | 0.98 | 1.00 | 0.36 | 1.00 | 0.10 | 1.00 | 0.15 | 1.00 |
|  | rs138568940 | 185595976 | 0.89 | 1.00 | 0.59 | 1.00 | 0.26 | 1.00 | 0.91 | 1.00 |
|  | rs72901831 | 185597040 | 0.78 | 1.00 | 0.74 | 1.00 | 0.30 | 1.00 | 0.38 | 1.00 |
|  | rs72901834 | 185598054 | 0.78 | 1.00 | 0.36 | 1.00 | 0.30 | 1.00 | 0.38 | 1.00 |
|  | rs10165596 | 185598108 | 0.98 | 1.00 | 0.36 | 1.00 | 0.10 | 1.00 | 0.15 | 1.00 |
|  | rs35328100 | 185598425 | 0.93 | 1.00 | 0.79 | 1.00 | 0.08 | 1.00 | 0.22 | 1.00 |
|  | rs72901836 | 185599046 | 0.78 | 1.00 | 0.39 | 1.00 | 0.30 | 1.00 | 0.38 | 1.00 |
|  | rs72901837 | 185599340 | 0.32 | 1.00 | 0.59 | 1.00 | 0.78 | 1.00 | 0.36 | 1.00 |
|  | rs80181049 | 185599568 | 0.07 | 1.00 | 0.70 | 1.00 | 0.44 | 1.00 | 0.18 | 1.00 |
|  | rs149063513 | 185600227 | 0.78 | 1.00 | 0.59 | 1.00 | 0.30 | 1.00 | 0.38 | 1.00 |
|  | rs72901840 | 185600802 | 0.78 | 1.00 | 0.91 | 1.00 | 0.30 | 1.00 | 0.38 | 1.00 |
|  | rs11693094 | 185601420 | 0.34 | 1.00 | 0.75 | 1.00 | 1.00 | 1.00 | 0.21 | 1.00 |
|  | 2:185601431:I | 185601431 | 0.82 | 1.00 | 0.74 | 1.00 | 0.46 | 1.00 | 0.59 | 1.00 |
|  | rs13023106 | 185601503 | 0.98 | 1.00 | 0.66 | 1.00 | 0.10 | 1.00 | 0.15 | 1.00 |
|  | 2:185601583:D | 185601583 | 0.66 | 1.00 | 0.75 | 1.00 | 0.79 | 1.00 | 0.68 | 1.00 |
|  | rs76903200 | 185601641 | 0.34 | 1.00 | 0.61 | 1.00 | 0.48 | 1.00 | 0.20 | 1.00 |
|  | rs7578333 | 185602002 | 0.80 | 1.00 | 0.91 | 1.00 | 0.43 | 1.00 | 0.48 | 1.00 |
|  | rs7581514 | 185602681 | 0.08 | 1.00 | 0.36 | 1.00 | 0.43 | 1.00 | 0.19 | 1.00 |
|  | 2:185602765:D | 185602765 | 1.00 | 1.00 | 0.74 | 1.00 | 0.10 | 1.00 | 0.16 | 1.00 |
|  | rs189804042 | 185602784 | 0.82 | 1.00 | 0.82 | 1.00 | 0.47 | 1.00 | 0.32 | 1.00 |
|  | rs10167859 | 185603817 | 0.83 | 1.00 | 0.38 | 1.00 | 0.45 | 1.00 | 0.53 | 1.00 |
|  | rs1480483 | 185604339 | 0.83 | 1.00 | 0.38 | 1.00 | 0.45 | 1.00 | 0.53 | 1.00 |
|  | rs79761896 | 185604892 | 0.05 | 0.99 | 0.74 | 1.00 | 1.00 | 1.00 | 0.23 | 1.00 |
|  | rs115370404 | 185604936 | 0.64 | 1.00 | 0.39 | 1.00 | 0.43 | 1.00 | 0.55 | 1.00 |
|  | rs13026742 | 185604982 | 0.18 | 1.00 | 0.49 | 1.00 | 0.93 | 1.00 | 0.63 | 1.00 |
|  | rs13001215 | 185605401 | 0.83 | 1.00 | 0.91 | 1.00 | 0.45 | 1.00 | 0.53 | 1.00 |
|  | rs72901849 | 185605471 | 0.76 | 1.00 | 0.36 | 1.00 | 0.33 | 1.00 | 0.39 | 1.00 |
|  | rs6434100 | 185606004 | 0.83 | 1.00 | 0.36 | 1.00 | 0.45 | 1.00 | 0.53 | 1.00 |
|  | rs140442054 | 185606420 | 0.28 | 1.00 | 0.59 | 1.00 | 0.53 | 1.00 | 0.47 | 1.00 |
|  | rs72901852 | 185606694 | 0.76 | 1.00 | 0.75 | 1.00 | 0.33 | 1.00 | 0.39 | 1.00 |
|  | 2:185607393:D | 185607393 | 0.83 | 1.00 | 0.74 | 1.00 | 0.45 | 1.00 | 0.53 | 1.00 |
|  | rs62176236 | 185607418 | 0.98 | 1.00 | 0.59 | 1.00 | 0.10 | 1.00 | 0.15 | 1.00 |
|  | rs34481141 | 185607757 | 0.94 | 1.00 | 0.77 | 1.00 | 0.12 | 1.00 | 0.18 | 1.00 |
|  | rs2170203 | 185609004 | 0.17 | 1.00 | 0.01 | 1.00 | 0.91 | 1.00 | 0.68 | 1.00 |
|  | rs113559691 | 185609217 | 0.09 | 1.00 | 0.74 | 1.00 | 0.29 | 1.00 | 0.96 | 1.00 |
|  | rs13404024 | 185609219 | 0.95 | 1.00 | 0.79 | 1.00 | 0.10 | 1.00 | 0.16 | 1.00 |
|  | rs115521688 | 185609477 | 0.75 | 1.00 | 0.42 | 1.00 | 0.21 | 1.00 | 0.30 | 1.00 |
|  | rs1480484 | 185609510 | 0.98 | 1.00 | 0.90 | 1.00 | 0.41 | 1.00 | 0.42 | 1.00 |
|  | rs1480485 | 185609755 | 0.88 | 1.00 | 0.74 | 1.00 | 0.37 | 1.00 | 0.39 | 1.00 |
|  | rs1480486 | 185609756 | 0.88 | 1.00 | 0.14 | 1.00 | 0.37 | 1.00 | 0.39 | 1.00 |
|  | rs115082372 | 185609871 | 0.05 | 0.99 | 0.15 | 1.00 | 0.62 | 1.00 | 0.15 | 1.00 |
|  | rs62176237 | 185610069 | 0.95 | 1.00 | 0.15 | 1.00 | 0.10 | 1.00 | 0.16 | 1.00 |
|  | rs115293799 | 185610686 | 0.98 | 1.00 | 0.58 | 1.00 | 0.55 | 1.00 | 0.26 | 1.00 |
|  | 2:185611161:D | 185611161 | 0.60 | 1.00 | 0.18 | 1.00 | 0.34 | 1.00 | 0.39 | 1.00 |
|  | rs180788873 | 185612032 | 0.98 | 1.00 | 0.47 | 1.00 | 0.26 | 1.00 | 0.37 | 1.00 |
|  | rs143662614 | 185612235 | 0.93 | 1.00 | 0.74 | 1.00 | 0.83 | 1.00 | 0.68 | 1.00 |
|  | rs72901857 | 185612603 | 0.81 | 1.00 | 0.36 | 1.00 | 0.32 | 1.00 | 0.36 | 1.00 |
|  | rs116405424 | 185613128 | 0.12 | 1.00 | 0.56 | 1.00 | 0.47 | 1.00 | 0.87 | 1.00 |
|  | rs114566971 | 185614749 | 0.30 | 1.00 | 0.74 | 1.00 | 0.87 | 1.00 | 0.81 | 1.00 |
|  | rs13384546 | 185616128 | 0.95 | 1.00 | 0.74 | 1.00 | 0.10 | 1.00 | 0.16 | 1.00 |
|  | rs145078188 | 185616233 | 0.02 | 0.90 | 0.74 | 0.86 | 0.21 | 1.00 | 0.16 | 1.00 |
|  | rs72901862 | 185617806 | 0.76 | 1.00 | 0.59 | 1.00 | 0.33 | 1.00 | 0.39 | 1.00 |
|  | rs13417466 | 185618111 | 0.98 | 1.00 | 0.59 | 1.00 | 0.41 | 1.00 | 0.42 | 1.00 |
|  | rs76430972 | 185619139 | 0.70 | 1.00 | 0.74 | 1.00 | 0.50 | 1.00 | 1.00 | 1.00 |
|  | rs76046974 | 185619499 | 0.70 | 1.00 | 0.63 | 1.00 | 0.07 | 1.00 | 0.30 | 1.00 |
|  | 2:185620821:D | 185620821 | 0.30 | 1.00 | 0.53 | 1.00 | 0.87 | 1.00 | 0.81 | 1.00 |
|  | rs10190062 | 185621495 | 0.98 | 1.00 | 0.36 | 1.00 | 0.41 | 1.00 | 0.42 | 1.00 |
|  | 2:185622534:D | 185622534 | 0.43 | 1.00 | 0.59 | 1.00 | 0.91 | 1.00 | 0.46 | 1.00 |
|  | rs10172363 | 185623051 | 0.93 | 1.00 | 0.59 | 1.00 | 0.10 | 1.00 | 0.17 | 1.00 |
|  | 2:185623103:D | 185623103 | 0.92 | 1.00 | 0.74 | 1.00 | 0.23 | 1.00 | 0.56 | 1.00 |
|  | 2:185623109:D | 185623109 | 0.95 | 1.00 | 0.36 | 1.00 | 0.21 | 1.00 | 0.73 | 1.00 |
|  | rs16826189 | 185623122 | 0.89 | 1.00 | 0.74 | 1.00 | 0.26 | 1.00 | 0.91 | 1.00 |
|  | 2:185623943:D | 185623943 | 0.27 | 1.00 | 0.59 | 1.00 | 0.12 | 1.00 | 0.19 | 1.00 |
|  | rs147921164 | 185624557 | 0.76 | 1.00 | 0.36 | 1.00 | 0.33 | 1.00 | 0.39 | 1.00 |
|  | rs12992805 | 185624638 | 0.23 | 1.00 | 0.97 | 1.00 | 0.78 | 1.00 | 0.11 | 1.00 |
|  | rs73978096 | 185625091 | 0.93 | 1.00 | 0.72 | 1.00 | 0.10 | 1.00 | 0.17 | 1.00 |
|  | rs72901873 | 185625279 | 0.76 | 1.00 | 0.36 | 1.00 | 0.33 | 1.00 | 0.39 | 1.00 |
|  | 2:185625396:D | 185625396 | 0.76 | 1.00 | 0.36 | 1.00 | 0.33 | 1.00 | 0.39 | 1.00 |
|  | rs10206254 | 185626176 | 0.93 | 1.00 | 0.59 | 1.00 | 0.10 | 1.00 | 0.17 | 1.00 |
|  | rs10206426 | 185626298 | 0.98 | 1.00 | 0.10 | 1.00 | 0.41 | 1.00 | 0.42 | 1.00 |
|  | rs2369595 | 185626426 | 0.07 | 1.00 | 0.74 | 1.00 | 0.68 | 1.00 | 0.20 | 1.00 |
|  | rs77224444 | 185627257 | 0.53 | 1.00 | 0.69 | 1.00 | 0.62 | 1.00 | 0.28 | 1.00 |
|  | rs13396662 | 185627999 | 0.93 | 1.00 | 0.36 | 1.00 | 0.10 | 1.00 | 0.17 | 1.00 |
|  | rs55701066 | 185628514 | 0.05 | 0.99 | 0.74 | 1.00 | 0.93 | 1.00 | 0.22 | 1.00 |
|  | rs4278895 | 185629633 | 0.98 | 1.00 | 0.59 | 1.00 | 0.41 | 1.00 | 0.42 | 1.00 |
|  | rs72901884 | 185629709 | 0.71 | 1.00 | 0.59 | 1.00 | 0.95 | 1.00 | 0.22 | 1.00 |
|  | rs6712125 | 185629756 | 0.93 | 1.00 | 0.74 | 1.00 | 0.10 | 1.00 | 0.17 | 1.00 |
|  | rs72901885 | 185630024 | 0.76 | 1.00 | 0.59 | 1.00 | 0.33 | 1.00 | 0.39 | 1.00 |
|  | rs2887514 | 185630128 | 0.98 | 1.00 | 0.91 | 1.00 | 0.41 | 1.00 | 0.42 | 1.00 |
|  | rs10172501 | 185630341 | 0.96 | 1.00 | 0.36 | 1.00 | 0.08 | 1.00 | 0.17 | 1.00 |
|  | rs72901889 | 185632346 | 0.76 | 1.00 | 0.66 | 1.00 | 0.33 | 1.00 | 0.39 | 1.00 |
|  | rs6728615 | 185632513 | 0.98 | 1.00 | 0.59 | 1.00 | 0.41 | 1.00 | 0.42 | 1.00 |
|  | rs10177035 | 185633046 | 0.98 | 1.00 | 0.39 | 1.00 | 0.41 | 1.00 | 0.42 | 1.00 |
|  | rs6732176 | 185633109 | 0.93 | 1.00 | 0.68 | 1.00 | 0.10 | 1.00 | 0.17 | 1.00 |
|  | rs72901891 | 185633794 | 0.76 | 1.00 | 0.96 | 1.00 | 0.33 | 1.00 | 0.39 | 1.00 |
|  | rs115682664 | 185633796 | 0.81 | 1.00 | 0.59 | 1.00 | 0.89 | 1.00 | 0.39 | 1.00 |
|  | rs72901893 | 185634241 | 0.76 | 1.00 | 0.74 | 1.00 | 0.33 | 1.00 | 0.39 | 1.00 |
|  | rs114932088 | 185634294 | 0.18 | 1.00 | 0.36 | 1.00 | 0.22 | 1.00 | 0.37 | 1.00 |
|  | rs10194277 | 185634659 | 0.93 | 1.00 | 0.29 | 1.00 | 0.10 | 1.00 | 0.17 | 1.00 |
|  | rs10194285 | 185634674 | 0.93 | 1.00 | 0.74 | 1.00 | 0.10 | 1.00 | 0.17 | 1.00 |
|  | rs10174301 | 185635853 | 0.93 | 1.00 | 0.36 | 1.00 | 0.10 | 1.00 | 0.17 | 1.00 |
|  | rs6759491 | 185636177 | 0.98 | 1.00 | 0.70 | 1.00 | 0.41 | 1.00 | 0.42 | 1.00 |
|  | 2:185636335:D | 185636335 | 0.76 | 1.00 | 0.38 | 1.00 | 0.33 | 1.00 | 0.39 | 1.00 |
|  | rs62176239 | 185636567 | 0.23 | 1.00 | 0.91 | 1.00 | 0.82 | 1.00 | 0.13 | 1.00 |
|  | rs6716151 | 185636617 | 0.98 | 1.00 | 0.65 | 1.00 | 0.41 | 1.00 | 0.42 | 1.00 |
|  | rs72901901 | 185637164 | 0.76 | 1.00 | 0.97 | 1.00 | 0.33 | 1.00 | 0.39 | 1.00 |
|  | rs12693388 | 185637684 | 0.07 | 1.00 | 0.13 | 1.00 | 0.74 | 1.00 | 0.27 | 1.00 |
|  | 2:185637708:D | 185637708 | 0.85 | 1.00 | 0.63 | 1.00 | 0.69 | 1.00 | 0.53 | 1.00 |
|  | rs17617913 | 185638875 | 0.55 | 1.00 | 0.66 | 1.00 | 0.59 | 1.00 | 0.29 | 1.00 |
|  | rs78011041 | 185639136 | 0.96 | 1.00 | 0.31 | 1.00 | 0.42 | 1.00 | 0.42 | 1.00 |
|  | rs142552000 | 185640673 | 0.24 | 1.00 | 0.74 | 1.00 | 0.99 | 1.00 | 0.85 | 1.00 |
|  | rs10196799 | 185640728 | 0.32 | 1.00 | 0.53 | 1.00 | 0.49 | 1.00 | 0.38 | 1.00 |
|  | rs116775825 | 185641160 | 0.65 | 1.00 | 0.74 | 1.00 | 0.63 | 1.00 | 0.29 | 1.00 |
|  | rs79156711 | 185641337 | 0.53 | 1.00 | 0.47 | 1.00 | 0.88 | 1.00 | 0.79 | 1.00 |
|  | rs12693389 | 185641495 | 0.93 | 1.00 | 0.40 | 1.00 | 0.10 | 1.00 | 0.17 | 1.00 |
|  | rs72903709 | 185641564 | 0.76 | 1.00 | 0.35 | 1.00 | 0.33 | 1.00 | 0.39 | 1.00 |
|  | rs10176668 | 185641635 | 0.93 | 1.00 | 0.33 | 1.00 | 0.10 | 1.00 | 0.17 | 1.00 |
|  | rs7603313 | 185641920 | 0.93 | 1.00 | 0.31 | 1.00 | 0.10 | 1.00 | 0.17 | 1.00 |
|  | rs6434102 | 185642580 | 0.98 | 1.00 | 0.76 | 1.00 | 0.41 | 1.00 | 0.42 | 1.00 |
|  | rs13018902 | 185642880 | 0.85 | 1.00 | 0.71 | 1.00 | 0.22 | 1.00 | 0.19 | 1.00 |
|  | rs17431119 | 185642960 | 0.76 | 1.00 | 0.76 | 1.00 | 0.33 | 1.00 | 0.39 | 1.00 |
|  | rs146350248 | 185643064 | 0.53 | 1.00 | 0.74 | 1.00 | 0.88 | 1.00 | 0.79 | 1.00 |
|  | rs17431132 | 185644113 | 0.93 | 1.00 | 0.31 | 1.00 | 0.10 | 1.00 | 0.17 | 1.00 |
|  | rs17431153 | 185644845 | 0.76 | 1.00 | 0.56 | 1.00 | 0.33 | 1.00 | 0.39 | 1.00 |
|  | rs13010468 | 185645106 | 0.95 | 1.00 | 0.71 | 1.00 | 0.10 | 1.00 | 0.08 | 1.00 |
|  | rs78785141 | 185645271 | 0.24 | 1.00 | 0.74 | 1.00 | 0.64 | 1.00 | 0.20 | 1.00 |
|  | rs10497657 | 185645840 | 0.76 | 1.00 | 0.74 | 1.00 | 0.33 | 1.00 | 0.39 | 1.00 |
|  | rs10497658 | 185646020 | 0.85 | 1.00 | 0.54 | 1.00 | 0.22 | 1.00 | 0.19 | 1.00 |
|  | rs13017390 | 185646076 | 0.93 | 1.00 | 0.93 | 1.00 | 0.10 | 1.00 | 0.17 | 1.00 |
|  | rs72903719 | 185647351 | 0.76 | 1.00 | 0.34 | 1.00 | 0.33 | 1.00 | 0.39 | 1.00 |
|  | rs72903720 | 185647475 | 0.76 | 1.00 | 0.74 | 1.00 | 0.33 | 1.00 | 0.39 | 1.00 |
|  | rs72903723 | 185647588 | 0.72 | 1.00 | 0.31 | 1.00 | 0.63 | 1.00 | 0.45 | 1.00 |
|  | rs1987025 | 185647595 | 0.98 | 1.00 | 0.31 | 1.00 | 0.41 | 1.00 | 0.42 | 1.00 |
|  | rs17584494 | 185647985 | 0.22 | 1.00 | 0.53 | 1.00 | 0.90 | 1.00 | 0.14 | 1.00 |
|  | rs72903727 | 185648068 | 0.86 | 1.00 | 0.74 | 1.00 | 0.11 | 1.00 | 0.02 | 0.98 |
|  | rs10497659 | 185648256 | 0.76 | 1.00 | 0.60 | 1.00 | 0.33 | 1.00 | 0.39 | 1.00 |
|  | rs116774374 | 185649149 | 0.60 | 1.00 | 0.74 | 1.00 | 0.98 | 1.00 | 0.71 | 1.00 |
|  | rs10497660 | 185649196 | 0.76 | 1.00 | 0.74 | 1.00 | 0.33 | 1.00 | 0.39 | 1.00 |
|  | rs1317210 | 185649555 | 0.93 | 1.00 | 0.53 | 1.00 | 0.10 | 1.00 | 0.17 | 1.00 |
|  | rs1320080 | 185650005 | 0.93 | 1.00 | 0.14 | 1.00 | 0.10 | 1.00 | 0.17 | 1.00 |
|  | rs17431258 | 185650079 | 0.76 | 1.00 | 0.70 | 1.00 | 0.33 | 1.00 | 0.39 | 1.00 |
|  | rs72903735 | 185650668 | 0.76 | 1.00 | 0.39 | 1.00 | 0.33 | 1.00 | 0.39 | 1.00 |
|  | 2:185651019:D | 185651019 | 0.93 | 1.00 | 0.80 | 1.00 | 0.10 | 1.00 | 0.17 | 1.00 |
|  | rs72903737 | 185651328 | 0.85 | 1.00 | 0.41 | 1.00 | 0.07 | 1.00 | 0.70 | 1.00 |
|  | 2:185652248:D | 185652248 | 0.96 | 1.00 | 0.74 | 1.00 | 0.42 | 1.00 | 0.43 | 1.00 |
|  | 2:185652255:D | 185652255 | 0.70 | 1.00 | 0.74 | 1.00 | 0.75 | 1.00 | 0.27 | 1.00 |
|  | rs10195510 | 185652653 | 0.93 | 1.00 | 0.66 | 1.00 | 0.10 | 1.00 | 0.17 | 1.00 |
|  | rs72903738 | 185654253 | 0.76 | 1.00 | 0.74 | 1.00 | 0.33 | 1.00 | 0.39 | 1.00 |
|  | rs6716235 | 185655092 | 0.98 | 1.00 | 0.31 | 1.00 | 0.41 | 1.00 | 0.42 | 1.00 |
|  | rs12693391 | 185655189 | 0.93 | 1.00 | 0.53 | 1.00 | 0.10 | 1.00 | 0.17 | 1.00 |
|  | rs4635495 | 185655317 | 0.76 | 1.00 | 0.56 | 1.00 | 0.33 | 1.00 | 0.39 | 1.00 |
|  | rs4275993 | 185655758 | 0.76 | 1.00 | 0.31 | 1.00 | 0.33 | 1.00 | 0.39 | 1.00 |
|  | rs34327168 | 185656085 | 0.93 | 1.00 | 0.74 | 1.00 | 0.10 | 1.00 | 0.17 | 1.00 |
|  | rs34963004 | 185656366 | 0.98 | 1.00 | 0.81 | 1.00 | 0.39 | 1.00 | 0.39 | 1.00 |
|  | rs34452663 | 185656920 | 0.93 | 1.00 | 0.31 | 1.00 | 0.10 | 1.00 | 0.17 | 1.00 |
|  | rs72903748 | 185658601 | 0.76 | 1.00 | 0.53 | 1.00 | 0.33 | 1.00 | 0.39 | 1.00 |
|  | rs12693392 | 185658811 | 0.93 | 1.00 | 0.53 | 1.00 | 0.10 | 1.00 | 0.17 | 1.00 |
|  | rs2369596 | 185658962 | 0.98 | 1.00 | 0.53 | 1.00 | 0.41 | 1.00 | 0.42 | 1.00 |
|  | rs72903752 | 185659789 | 0.76 | 1.00 | 0.31 | 1.00 | 0.33 | 1.00 | 0.39 | 1.00 |
|  | rs62176258 | 185659951 | 0.93 | 1.00 | 0.74 | 1.00 | 0.10 | 1.00 | 0.17 | 1.00 |
|  | rs58159162 | 185660672 | 0.93 | 1.00 | 0.70 | 1.00 | 0.10 | 1.00 | 0.17 | 1.00 |
|  | 2:185660677:I | 185660677 | 0.64 | 1.00 | 0.31 | 1.00 | 0.29 | 1.00 | 0.51 | 1.00 |
|  | rs10170664 | 185661400 | 0.18 | 1.00 | 0.31 | 1.00 | 0.73 | 1.00 | 0.71 | 1.00 |
|  | rs12693394 | 185661580 | 0.98 | 1.00 | 0.74 | 1.00 | 0.41 | 1.00 | 0.42 | 1.00 |
|  | rs72903757 | 185661854 | 0.87 | 1.00 | 0.74 | 1.00 | 0.38 | 1.00 | 0.58 | 1.00 |
|  | rs13028549 | 185661907 | 0.98 | 1.00 | 0.70 | 1.00 | 0.41 | 1.00 | 0.42 | 1.00 |
|  | rs79818207 | 185662685 | 0.94 | 1.00 | 0.49 | 1.00 | 0.93 | 1.00 | 0.59 | 1.00 |
|  | rs4666990 | 185663304 | 0.40 | 1.00 | 0.70 | 1.00 | 0.57 | 1.00 | 0.49 | 1.00 |
|  | rs72903763 | 185663591 | 0.76 | 1.00 | 0.74 | 1.00 | 0.33 | 1.00 | 0.39 | 1.00 |
|  | rs62176259 | 185663735 | 0.25 | 1.00 | 0.62 | 1.00 | 0.43 | 1.00 | 0.60 | 1.00 |
|  | rs112725983 | 185663954 | 0.34 | 1.00 | 0.53 | 1.00 | 0.48 | 1.00 | 0.20 | 1.00 |
|  | 2:185664034:I | 185664034 | 0.53 | 1.00 | 0.02 | 1.00 | 0.99 | 1.00 | 0.64 | 1.00 |
|  | rs6731662 | 185664433 | 0.43 | 1.00 | 0.48 | 1.00 | 0.39 | 1.00 | 0.20 | 1.00 |
|  | rs4294964 | 185664579 | 0.93 | 1.00 | 0.74 | 1.00 | 0.10 | 1.00 | 0.17 | 1.00 |
|  | rs72903765 | 185664802 | 0.76 | 1.00 | 0.53 | 1.00 | 0.33 | 1.00 | 0.39 | 1.00 |
|  | 2:185665079:D | 185665079 | 0.68 | 1.00 | 0.74 | 1.00 | 0.36 | 1.00 | 0.40 | 1.00 |
|  | rs62178060 | 185665080 | 0.97 | 1.00 | 0.53 | 1.00 | 0.13 | 1.00 | 0.16 | 1.00 |
|  | 2:185665083:D | 185665083 | 0.97 | 1.00 | 0.31 | 1.00 | 0.13 | 1.00 | 0.16 | 1.00 |
|  | rs72903769 | 185665389 | 0.76 | 1.00 | 0.54 | 1.00 | 0.33 | 1.00 | 0.39 | 1.00 |
|  | rs72903774 | 185667909 | 0.45 | 1.00 | 0.75 | 1.00 | 0.70 | 1.00 | 0.09 | 1.00 |
|  | rs74515723 | 185668594 | 0.80 | 1.00 | 0.74 | 1.00 | 0.44 | 1.00 | 0.43 | 1.00 |
|  | rs182013686 | 185668595 | 0.69 | 1.00 | 0.53 | 1.00 | 0.72 | 1.00 | 0.93 | 1.00 |
|  | rs6751335 | 185669127 | 0.93 | 1.00 | 0.53 | 1.00 | 0.10 | 1.00 | 0.17 | 1.00 |
|  | rs10211213 | 185670083 | 0.93 | 1.00 | 0.71 | 1.00 | 0.10 | 1.00 | 0.17 | 1.00 |
|  | rs13012893 | 185670332 | 0.98 | 1.00 | 0.75 | 1.00 | 0.41 | 1.00 | 0.42 | 1.00 |
|  | rs74961493 | 185670496 | 0.34 | 1.00 | 0.95 | 1.00 | 0.48 | 1.00 | 0.20 | 1.00 |
|  | 2:185671823:I | 185671823 | 0.84 | 1.00 | 0.95 | 1.00 | 0.41 | 1.00 | 0.28 | 1.00 |
|  | rs13012514 | 185672183 | 0.98 | 1.00 | 0.20 | 1.00 | 0.41 | 1.00 | 0.42 | 1.00 |
|  | rs72903776 | 185672750 | 0.72 | 1.00 | 0.80 | 1.00 | 0.33 | 1.00 | 0.35 | 1.00 |
|  | rs192150910 | 185672862 | 0.00 | 0.63 | 0.88 | 0.72 | 0.33 | 1.00 | 0.14 | 1.00 |
|  | rs72903778 | 185673214 | 0.76 | 1.00 | 0.80 | 1.00 | 0.33 | 1.00 | 0.39 | 1.00 |
|  | rs72903783 | 185673390 | 0.80 | 1.00 | 0.31 | 1.00 | 0.43 | 1.00 | 0.53 | 1.00 |
|  | rs111978801 | 185673479 | 0.99 | 1.00 | 0.96 | 1.00 | 0.21 | 1.00 | 0.25 | 1.00 |
|  | rs143934924 | 185674034 | 0.90 | 1.00 | 0.48 | 1.00 | 0.97 | 1.00 | 0.86 | 1.00 |
|  | rs72903786 | 185674302 | 0.76 | 1.00 | 0.34 | 1.00 | 0.33 | 1.00 | 0.39 | 1.00 |
|  | 2:185675344:D | 185675344 | 0.09 | 1.00 | 0.53 | 1.00 | 0.80 | 1.00 | 0.14 | 1.00 |
|  | rs55963267 | 185675345 | 0.09 | 1.00 | 0.34 | 1.00 | 0.84 | 1.00 | 0.12 | 1.00 |
|  | rs56341618 | 185675346 | 0.09 | 1.00 | 0.70 | 1.00 | 0.84 | 1.00 | 0.12 | 1.00 |
|  | rs34943323 | 185675474 | 0.22 | 1.00 | 0.74 | 1.00 | 0.50 | 1.00 | 0.25 | 1.00 |
|  | 2:185676179:I | 185676179 | 0.11 | 1.00 | 0.74 | 1.00 | 0.91 | 1.00 | 0.17 | 1.00 |
|  | rs143598180 | 185677263 | 0.30 | 1.00 | 0.80 | 1.00 | 0.83 | 1.00 | 0.83 | 1.00 |
|  | rs17509336 | 185677806 | 0.76 | 1.00 | 0.95 | 1.00 | 0.33 | 1.00 | 0.39 | 1.00 |
|  | rs17431278 | 185678006 | 0.93 | 1.00 | 0.48 | 1.00 | 0.10 | 1.00 | 0.17 | 1.00 |
|  | rs11690438 | 185678750 | 0.44 | 1.00 | 0.96 | 1.00 | 0.96 | 1.00 | 0.66 | 1.00 |
|  | rs72903792 | 185679980 | 0.76 | 1.00 | 0.80 | 1.00 | 0.33 | 1.00 | 0.39 | 1.00 |
|  | 2:185679982:D | 185679982 | 0.76 | 1.00 | 0.74 | 1.00 | 0.33 | 1.00 | 0.39 | 1.00 |
|  | rs72903794 | 185679983 | 0.76 | 1.00 | 0.47 | 1.00 | 0.33 | 1.00 | 0.39 | 1.00 |
|  | rs10201360 | 185680799 | 0.98 | 1.00 | 0.92 | 1.00 | 0.41 | 1.00 | 0.42 | 1.00 |
|  | rs13408744 | 185681052 | 0.98 | 1.00 | 0.74 | 1.00 | 0.41 | 1.00 | 0.42 | 1.00 |
|  | rs72903798 | 185681546 | 0.76 | 1.00 | 0.74 | 1.00 | 0.33 | 1.00 | 0.39 | 1.00 |
|  | rs7608386 | 185681713 | 0.98 | 1.00 | 0.31 | 1.00 | 0.42 | 1.00 | 0.39 | 1.00 |
|  | rs17584522 | 185681730 | 0.20 | 1.00 | 0.74 | 1.00 | 0.54 | 1.00 | 0.20 | 1.00 |
|  | rs13386789 | 185681984 | 0.93 | 1.00 | 0.68 | 1.00 | 0.10 | 1.00 | 0.17 | 1.00 |
|  | rs6720863 | 185682700 | 0.98 | 1.00 | 0.31 | 1.00 | 0.41 | 1.00 | 0.42 | 1.00 |
|  | rs6749691 | 185682739 | 0.98 | 1.00 | 0.95 | 1.00 | 0.41 | 1.00 | 0.42 | 1.00 |
|  | rs72905708 | 185683614 | 0.76 | 1.00 | 0.69 | 1.00 | 0.33 | 1.00 | 0.39 | 1.00 |
|  | rs2369589 | 185683942 | 0.93 | 1.00 | 0.89 | 1.00 | 0.10 | 1.00 | 0.17 | 1.00 |
|  | rs72905711 | 185685333 | 0.76 | 1.00 | 0.80 | 1.00 | 0.33 | 1.00 | 0.39 | 1.00 |
|  | rs4614893 | 185685591 | 0.98 | 1.00 | 0.34 | 1.00 | 0.41 | 1.00 | 0.42 | 1.00 |
|  | rs4458174 | 185685607 | 0.93 | 1.00 | 0.84 | 1.00 | 0.10 | 1.00 | 0.17 | 1.00 |
|  | rs149580710 | 185685947 | 0.63 | 1.00 | 0.78 | 1.00 | 0.56 | 1.00 | 0.31 | 1.00 |
|  | rs142287606 | 185686225 | 0.49 | 1.00 | 0.31 | 1.00 | 0.74 | 1.00 | 0.51 | 1.00 |
|  | rs35071909 | 185686895 | 0.93 | 1.00 | 0.31 | 1.00 | 0.10 | 1.00 | 0.17 | 1.00 |
|  | rs62174661 | 185687954 | 0.93 | 1.00 | 0.47 | 1.00 | 0.10 | 1.00 | 0.17 | 1.00 |
|  | rs13396495 | 185688076 | 0.98 | 1.00 | 0.31 | 1.00 | 0.41 | 1.00 | 0.42 | 1.00 |
|  | rs190748553 | 185688204 | 0.28 | 1.00 | 0.57 | 1.00 | 0.20 | 1.00 | 0.41 | 1.00 |
|  | rs35105890 | 185688786 | 0.76 | 1.00 | 0.74 | 1.00 | 0.33 | 1.00 | 0.39 | 1.00 |
|  | rs189530005 | 185689599 | 0.97 | 1.00 | 0.74 | 1.00 | 0.54 | 1.00 | 1.00 | 1.00 |
|  | rs4544394 | 185689671 | 0.93 | 1.00 | 0.74 | 1.00 | 0.10 | 1.00 | 0.17 | 1.00 |
|  | rs72905716 | 185690060 | 0.76 | 1.00 | 0.31 | 1.00 | 0.33 | 1.00 | 0.39 | 1.00 |
|  | rs9973969 | 185690630 | 0.98 | 1.00 | 0.42 | 1.00 | 0.41 | 1.00 | 0.42 | 1.00 |
|  | rs9973631 | 185690911 | 0.98 | 1.00 | 0.53 | 1.00 | 0.41 | 1.00 | 0.42 | 1.00 |
|  | rs72905722 | 185691444 | 0.76 | 1.00 | 0.80 | 1.00 | 0.33 | 1.00 | 0.39 | 1.00 |
|  | rs13424507 | 185691625 | 0.98 | 1.00 | 0.51 | 1.00 | 0.41 | 1.00 | 0.42 | 1.00 |
|  | rs6736418 | 185692224 | 0.49 | 1.00 | 0.74 | 1.00 | 0.28 | 1.00 | 0.29 | 1.00 |
|  | rs9973668 | 185692497 | 0.93 | 1.00 | 0.74 | 1.00 | 0.10 | 1.00 | 0.17 | 1.00 |
|  | rs190975828 | 185693019 | 0.61 | 1.00 | 0.19 | 1.00 | 0.17 | 1.00 | 0.49 | 1.00 |
|  | rs13028349 | 185693549 | 0.98 | 1.00 | 0.31 | 1.00 | 0.41 | 1.00 | 0.42 | 1.00 |
|  | rs187209431 | 185693790 | 0.45 | 1.00 | 0.80 | 1.00 | 0.70 | 1.00 | 0.09 | 1.00 |
|  | rs142738518 | 185694731 | 0.60 | 1.00 | 0.54 | 1.00 | 0.98 | 1.00 | 0.71 | 1.00 |
|  | rs76585359 | 185695954 | 0.94 | 1.00 | 0.34 | 1.00 | 0.99 | 1.00 | 0.85 | 1.00 |
|  | rs16826219 | 185696114 | 0.98 | 1.00 | 0.55 | 1.00 | 0.41 | 1.00 | 0.42 | 1.00 |
|  | rs72905730 | 185696271 | 0.76 | 1.00 | 0.31 | 1.00 | 0.33 | 1.00 | 0.39 | 1.00 |
|  | rs12693395 | 185696607 | 0.93 | 1.00 | 0.31 | 1.00 | 0.10 | 1.00 | 0.17 | 1.00 |
|  | 2:185697403:D | 185697403 | 0.90 | 1.00 | 0.44 | 1.00 | 0.11 | 1.00 | 0.20 | 1.00 |
|  | rs17509517 | 185700607 | 0.76 | 1.00 | 0.81 | 1.00 | 0.33 | 1.00 | 0.39 | 1.00 |
|  | rs62174663 | 185700770 | 0.93 | 1.00 | 0.80 | 1.00 | 0.10 | 1.00 | 0.17 | 1.00 |
|  | 2:185700806:I | 185700806 | 0.87 | 1.00 | 0.31 | 1.00 | 0.38 | 1.00 | 0.58 | 1.00 |
|  | 2:185701410:D | 185701410 | 0.84 | 1.00 | 0.69 | 1.00 | 0.09 | 1.00 | 0.10 | 1.00 |
|  | 2:185701422:D | 185701422 | 0.44 | 1.00 | 0.18 | 1.00 | 0.42 | 1.00 | 0.06 | 1.00 |
|  | 2:185701423:D | 185701423 | 0.28 | 1.00 | 0.81 | 1.00 | 0.52 | 1.00 | 0.08 | 1.00 |
|  | 2:185701425:I | 185701425 | 0.87 | 1.00 | 0.31 | 1.00 | 0.78 | 1.00 | 0.65 | 1.00 |
|  | rs141701360 | 185701426 | 0.10 | 1.00 | 0.21 | 1.00 | 0.75 | 1.00 | 0.63 | 1.00 |
|  | rs72905734 | 185701706 | 0.60 | 1.00 | 0.44 | 1.00 | 0.94 | 1.00 | 0.13 | 1.00 |
|  | rs72905736 | 185701858 | 0.61 | 1.00 | 0.80 | 1.00 | 0.17 | 1.00 | 0.49 | 1.00 |
|  | rs115103722 | 185702685 | 0.79 | 1.00 | 0.74 | 1.00 | 0.21 | 1.00 | 0.36 | 1.00 |
|  | 2:185702844:D | 185702844 | 0.76 | 1.00 | 0.80 | 1.00 | 0.33 | 1.00 | 0.39 | 1.00 |
|  | rs7605689 | 185705856 | 0.97 | 1.00 | 0.57 | 1.00 | 0.34 | 1.00 | 0.36 | 1.00 |
|  | rs72905739 | 185706202 | 0.76 | 1.00 | 0.96 | 1.00 | 0.33 | 1.00 | 0.39 | 1.00 |
|  | rs72905741 | 185707251 | 0.76 | 1.00 | 0.31 | 1.00 | 0.44 | 1.00 | 0.44 | 1.00 |
|  | rs75862412 | 185707252 | 0.77 | 1.00 | 0.31 | 1.00 | 0.08 | 1.00 | 0.11 | 1.00 |
|  | rs56049830 | 185708081 | 0.52 | 1.00 | 0.81 | 1.00 | 0.49 | 1.00 | 0.30 | 1.00 |
|  | rs4494699 | 185708165 | 0.63 | 1.00 | 0.51 | 1.00 | 0.33 | 1.00 | 0.83 | 1.00 |
|  | rs10180597 | 185708432 | 0.94 | 1.00 | 0.31 | 1.00 | 0.08 | 1.00 | 0.13 | 1.00 |
|  | 2:185708986:D | 185708986 | 0.75 | 1.00 | 0.74 | 1.00 | 0.34 | 1.00 | 0.35 | 1.00 |
|  | rs7603015 | 185708987 | 0.86 | 1.00 | 0.75 | 1.00 | 0.39 | 1.00 | 0.53 | 1.00 |
|  | rs72905747 | 185708989 | 0.75 | 1.00 | 0.58 | 1.00 | 0.34 | 1.00 | 0.35 | 1.00 |
|  | rs72905749 | 185710079 | 0.76 | 1.00 | 0.75 | 1.00 | 0.33 | 1.00 | 0.39 | 1.00 |
|  | rs10187190 | 185710178 | 0.94 | 1.00 | 0.32 | 1.00 | 0.08 | 1.00 | 0.13 | 1.00 |
|  | rs138273718 | 185710444 | 0.86 | 1.00 | 0.74 | 1.00 | 0.49 | 1.00 | 0.98 | 1.00 |
|  | rs184075698 | 185710846 | 0.43 | 1.00 | 0.18 | 1.00 | 0.65 | 1.00 | 0.02 | 0.97 |
|  | rs72905751 | 185713166 | 0.76 | 1.00 | 0.80 | 1.00 | 0.33 | 1.00 | 0.39 | 1.00 |
|  | rs72905753 | 185713304 | 0.76 | 1.00 | 0.02 | 1.00 | 0.33 | 1.00 | 0.39 | 1.00 |
|  | rs34597412 | 185715275 | 0.20 | 1.00 | 0.58 | 1.00 | 0.50 | 1.00 | 0.54 | 1.00 |
|  | rs146362735 | 185715304 | 0.48 | 1.00 | 0.58 | 1.00 | 0.43 | 1.00 | 0.35 | 1.00 |
|  | rs114199508 | 185716210 | 0.97 | 1.00 | 0.58 | 1.00 | 0.14 | 1.00 | 0.81 | 1.00 |
|  | 2:185716952:D | 185716952 | 0.76 | 1.00 | 0.74 | 1.00 | 0.33 | 1.00 | 0.39 | 1.00 |
|  | rs6709440 | 185717491 | 0.94 | 1.00 | 0.52 | 1.00 | 0.08 | 1.00 | 0.13 | 1.00 |
|  | 2:185717550:D | 185717550 | 0.94 | 1.00 | 0.63 | 1.00 | 0.08 | 1.00 | 0.13 | 1.00 |
|  | rs7578316 | 185718167 | 0.97 | 1.00 | 0.58 | 1.00 | 0.34 | 1.00 | 0.36 | 1.00 |
|  | 2:185718352:I | 185718352 | 0.76 | 1.00 | 0.75 | 1.00 | 0.33 | 1.00 | 0.39 | 1.00 |
|  | rs80268465 | 185719122 | 0.11 | 1.00 | 0.75 | 1.00 | 0.21 | 1.00 | 0.89 | 1.00 |
|  | rs72905760 | 185719125 | 0.76 | 1.00 | 0.63 | 1.00 | 0.33 | 1.00 | 0.39 | 1.00 |
|  | 2:185719140:I | 185719140 | 0.76 | 1.00 | 0.96 | 1.00 | 0.33 | 1.00 | 0.39 | 1.00 |
|  | rs12693396 | 185719174 | 0.97 | 1.00 | 0.63 | 1.00 | 0.34 | 1.00 | 0.36 | 1.00 |
|  | 2:185719523:D | 185719523 | 0.06 | 1.00 | 0.90 | 1.00 | 0.96 | 1.00 | 0.21 | 1.00 |
|  | rs6748925 | 185719686 | 0.87 | 1.00 | 0.90 | 1.00 | 0.38 | 1.00 | 0.58 | 1.00 |
|  | rs72905766 | 185719872 | 0.45 | 1.00 | 0.53 | 1.00 | 0.70 | 1.00 | 0.09 | 1.00 |
|  | 2:185720338:D | 185720338 | 0.66 | 1.00 | 0.86 | 1.00 | 0.32 | 1.00 | 0.51 | 1.00 |
|  | rs75530945 | 185720402 | 0.97 | 1.00 | 0.63 | 1.00 | 0.20 | 1.00 | 0.15 | 1.00 |
|  | rs55941754 | 185720926 | 0.76 | 1.00 | 0.85 | 1.00 | 0.33 | 1.00 | 0.39 | 1.00 |
|  | rs56217433 | 185721111 | 0.76 | 1.00 | 0.58 | 1.00 | 0.33 | 1.00 | 0.39 | 1.00 |
|  | rs72905773 | 185721156 | 0.67 | 1.00 | 0.58 | 1.00 | 0.20 | 1.00 | 0.66 | 1.00 |
|  | rs72905774 | 185721279 | 0.76 | 1.00 | 0.74 | 1.00 | 0.33 | 1.00 | 0.39 | 1.00 |
|  | rs62174664 | 185721319 | 0.94 | 1.00 | 0.63 | 1.00 | 0.08 | 1.00 | 0.13 | 1.00 |
|  | rs35321501 | 185721692 | 0.97 | 1.00 | 0.65 | 1.00 | 0.34 | 1.00 | 0.36 | 1.00 |
|  | rs79577277 | 185722370 | 0.86 | 1.00 | 0.80 | 1.00 | 0.49 | 1.00 | 0.98 | 1.00 |
|  | rs13386192 | 185722380 | 0.94 | 1.00 | 0.68 | 1.00 | 0.08 | 1.00 | 0.13 | 1.00 |
|  | rs72905777 | 185722863 | 0.76 | 1.00 | 0.74 | 1.00 | 0.33 | 1.00 | 0.39 | 1.00 |
|  | rs7589314 | 185723002 | 0.92 | 1.00 | 0.71 | 1.00 | 0.62 | 1.00 | 0.38 | 1.00 |
|  | rs13415342 | 185723188 | 0.94 | 1.00 | 1.00 | 1.00 | 0.08 | 1.00 | 0.13 | 1.00 |
|  | rs7577581 | 185723479 | 0.97 | 1.00 | 0.28 | 1.00 | 0.34 | 1.00 | 0.36 | 1.00 |
|  | rs7593307 | 185724193 | 0.97 | 1.00 | 0.74 | 1.00 | 0.34 | 1.00 | 0.36 | 1.00 |
|  | rs12693397 | 185724435 | 0.97 | 1.00 | 0.23 | 1.00 | 0.34 | 1.00 | 0.36 | 1.00 |
|  | rs12693398 | 185724450 | 0.94 | 1.00 | 0.00 | 1.00 | 0.08 | 1.00 | 0.13 | 1.00 |
|  | rs72905782 | 185724464 | 0.76 | 1.00 | 0.96 | 1.00 | 0.33 | 1.00 | 0.39 | 1.00 |
|  | rs72905784 | 185724642 | 0.87 | 1.00 | 0.63 | 1.00 | 0.38 | 1.00 | 0.58 | 1.00 |
|  | rs13396624 | 185725062 | 0.94 | 1.00 | 0.28 | 1.00 | 0.08 | 1.00 | 0.13 | 1.00 |
|  | rs13422256 | 185725088 | 0.94 | 1.00 | 0.74 | 1.00 | 0.08 | 1.00 | 0.13 | 1.00 |
|  | rs72905785 | 185725119 | 0.76 | 1.00 | 0.69 | 1.00 | 0.33 | 1.00 | 0.39 | 1.00 |
|  | rs72905786 | 185725574 | 0.76 | 1.00 | 0.14 | 1.00 | 0.33 | 1.00 | 0.39 | 1.00 |
|  | rs7585171 | 185725617 | 0.87 | 1.00 | 0.52 | 1.00 | 0.38 | 1.00 | 0.58 | 1.00 |
|  | rs72905792 | 185726138 | 0.48 | 1.00 | 0.50 | 1.00 | 0.84 | 1.00 | 0.47 | 1.00 |
|  | rs72905794 | 185726221 | 0.87 | 1.00 | 0.64 | 1.00 | 0.38 | 1.00 | 0.58 | 1.00 |
|  | rs72905795 | 185727333 | 0.76 | 1.00 | 0.60 | 1.00 | 0.33 | 1.00 | 0.39 | 1.00 |
|  | rs62200787 | 185727353 | 0.20 | 1.00 | 0.99 | 1.00 | 0.39 | 1.00 | 0.18 | 1.00 |
|  | 2:185727392:I | 185727392 | 0.97 | 1.00 | 0.62 | 1.00 | 0.34 | 1.00 | 0.36 | 1.00 |
|  | rs148954956 | 185727583 | 0.03 | 0.95 | 0.99 | 0.98 | 0.32 | 1.00 | 0.20 | 1.00 |
|  | rs62200788 | 185727845 | 0.19 | 1.00 | 0.62 | 1.00 | 0.59 | 1.00 | 0.20 | 1.00 |
|  | rs72905797 | 185727861 | 0.76 | 1.00 | 0.62 | 1.00 | 0.33 | 1.00 | 0.39 | 1.00 |
|  | rs7558878 | 185729162 | 0.97 | 1.00 | 0.78 | 1.00 | 0.34 | 1.00 | 0.36 | 1.00 |
|  | 2:185729166:D | 185729166 | 0.76 | 1.00 | 0.81 | 1.00 | 0.33 | 1.00 | 0.39 | 1.00 |
|  | rs11890843 | 185729304 | 0.97 | 1.00 | 0.33 | 1.00 | 0.34 | 1.00 | 0.36 | 1.00 |
|  | rs13398732 | 185730587 | 0.94 | 1.00 | 0.77 | 1.00 | 0.08 | 1.00 | 0.13 | 1.00 |
|  | rs78743672 | 185731420 | 0.84 | 1.00 | 0.78 | 1.00 | 0.46 | 1.00 | 0.77 | 1.00 |
|  | rs115475004 | 185731421 | 0.48 | 1.00 | 0.59 | 1.00 | 0.69 | 1.00 | 0.58 | 1.00 |
|  | rs114523990 | 185731591 | 0.76 | 1.00 | 0.57 | 1.00 | 0.33 | 1.00 | 0.39 | 1.00 |
|  | rs62200789 | 185731605 | 0.97 | 1.00 | 0.23 | 1.00 | 0.34 | 1.00 | 0.36 | 1.00 |
|  | rs62200790 | 185731622 | 0.97 | 1.00 | 0.42 | 1.00 | 0.34 | 1.00 | 0.36 | 1.00 |
|  | rs17509601 | 185731901 | 0.79 | 1.00 | 0.49 | 1.00 | 0.32 | 1.00 | 0.37 | 1.00 |
|  | rs17509608 | 185732578 | 0.72 | 1.00 | 0.65 | 1.00 | 0.32 | 1.00 | 0.64 | 1.00 |
|  | rs13388087 | 185734704 | 0.99 | 1.00 | 0.35 | 1.00 | 0.93 | 1.00 | 0.77 | 1.00 |
|  | rs13401381 | 185734749 | 0.99 | 1.00 | 0.45 | 1.00 | 0.93 | 1.00 | 0.77 | 1.00 |
|  | rs71430164 | 185735048 | 0.12 | 1.00 | 0.66 | 1.00 | 0.98 | 1.00 | 0.38 | 1.00 |
|  | rs7588907 | 185735599 | 0.94 | 1.00 | 0.94 | 1.00 | 0.61 | 1.00 | 0.36 | 1.00 |
|  | rs17509622 | 185735643 | 0.63 | 1.00 | 0.32 | 1.00 | 0.69 | 1.00 | 0.72 | 1.00 |
|  | rs7564941 | 185735813 | 0.94 | 1.00 | 0.66 | 1.00 | 0.61 | 1.00 | 0.36 | 1.00 |
|  | rs10199843 | 185736625 | 0.94 | 1.00 | 0.44 | 1.00 | 0.08 | 1.00 | 0.13 | 1.00 |
|  | rs181721986 | 185736981 | 0.94 | 1.00 | 0.46 | 1.00 | 0.99 | 1.00 | 0.85 | 1.00 |
|  | rs142058560 | 185737844 | 0.66 | 1.00 | 0.17 | 1.00 | 0.57 | 1.00 | 0.99 | 1.00 |
|  | rs78167161 | 185738134 | 0.77 | 1.00 | 0.59 | 1.00 | 0.25 | 1.00 | 0.13 | 1.00 |
|  | rs6709436 | 185738633 | 0.97 | 1.00 | 0.17 | 1.00 | 0.34 | 1.00 | 0.36 | 1.00 |
|  | rs116769903 | 185738697 | 0.76 | 1.00 | 0.37 | 1.00 | 0.27 | 1.00 | 0.22 | 1.00 |
|  | rs11902536 | 185738735 | 0.87 | 1.00 | 0.66 | 1.00 | 0.38 | 1.00 | 0.58 | 1.00 |
|  | rs17431582 | 185739015 | 0.76 | 1.00 | 0.50 | 1.00 | 0.33 | 1.00 | 0.39 | 1.00 |
|  | rs1366845 | 185739226 | 0.76 | 1.00 | 0.32 | 1.00 | 0.33 | 1.00 | 0.39 | 1.00 |
|  | rs1366844 | 185739347 | 0.94 | 1.00 | 0.84 | 1.00 | 0.61 | 1.00 | 0.36 | 1.00 |
|  | rs10210216 | 185740015 | 0.99 | 1.00 | 0.17 | 1.00 | 0.93 | 1.00 | 0.77 | 1.00 |
|  | rs142811142 | 185741068 | 0.66 | 1.00 | 0.66 | 1.00 | 0.57 | 1.00 | 0.99 | 1.00 |
|  | rs77689531 | 185741079 | 0.94 | 1.00 | 0.44 | 1.00 | 0.99 | 1.00 | 0.85 | 1.00 |
|  | rs11892843 | 185741134 | 0.94 | 1.00 | 0.32 | 1.00 | 0.61 | 1.00 | 0.36 | 1.00 |
|  | rs17431603 | 185741744 | 0.76 | 1.00 | 0.07 | 1.00 | 0.33 | 1.00 | 0.39 | 1.00 |
|  | rs115166888 | 185741898 | 0.81 | 1.00 | 0.57 | 1.00 | 0.14 | 1.00 | 0.59 | 1.00 |
|  | rs72907715 | 185742038 | 0.50 | 1.00 | 0.63 | 1.00 | 0.47 | 1.00 | 0.33 | 1.00 |
|  | rs1366843 | 185742271 | 0.76 | 1.00 | 0.63 | 1.00 | 0.33 | 1.00 | 0.39 | 1.00 |
|  | rs1820847 | 185742474 | 0.76 | 1.00 | 0.19 | 1.00 | 0.33 | 1.00 | 0.39 | 1.00 |
|  | rs4586602 | 185742794 | 0.94 | 1.00 | 0.36 | 1.00 | 0.08 | 1.00 | 0.13 | 1.00 |
|  | rs72907717 | 185742905 | 0.76 | 1.00 | 0.48 | 1.00 | 0.33 | 1.00 | 0.39 | 1.00 |
|  | rs143448052 | 185743232 | 0.53 | 1.00 | 0.40 | 1.00 | 0.88 | 1.00 | 0.79 | 1.00 |
|  | rs13411652 | 185743241 | 0.94 | 1.00 | 0.11 | 1.00 | 0.08 | 1.00 | 0.13 | 1.00 |
|  | rs1429427 | 185743953 | 0.99 | 1.00 | 0.19 | 1.00 | 0.93 | 1.00 | 0.77 | 1.00 |
|  | rs34233935 | 185744192 | 0.23 | 1.00 | 0.45 | 1.00 | 0.34 | 1.00 | 0.57 | 1.00 |
|  | rs138223121 | 185744346 | 0.74 | 1.00 | 0.86 | 1.00 | 0.52 | 1.00 | 0.41 | 1.00 |
|  | rs10206265 | 185744352 | 0.94 | 1.00 | 0.80 | 1.00 | 0.61 | 1.00 | 0.36 | 1.00 |
|  | rs72907719 | 185745056 | 0.85 | 1.00 | 0.64 | 1.00 | 0.18 | 1.00 | 0.05 | 1.00 |
|  | 2:185745189:D | 185745189 | 0.69 | 1.00 | 0.26 | 1.00 | 0.39 | 1.00 | 0.48 | 1.00 |
|  | rs66983648 | 185745195 | 1.00 | 1.00 | 0.79 | 1.00 | 0.66 | 1.00 | 0.45 | 1.00 |
|  | rs112869258 | 185745353 | 0.79 | 1.00 | 0.39 | 1.00 | 0.21 | 1.00 | 0.36 | 1.00 |
|  | 2:185745400:I | 185745400 | 0.94 | 1.00 | 0.02 | 1.00 | 0.08 | 1.00 | 0.13 | 1.00 |
|  | rs112372472 | 185745500 | 0.28 | 1.00 | 0.97 | 1.00 | 0.81 | 1.00 | 0.43 | 1.00 |
|  | rs34945978 | 185745869 | 0.94 | 1.00 | 0.97 | 1.00 | 0.08 | 1.00 | 0.13 | 1.00 |
|  | rs116621013 | 185747009 | 0.75 | 1.00 | 0.27 | 1.00 | 0.18 | 1.00 | 0.32 | 1.00 |
|  | rs72907724 | 185748032 | 0.76 | 1.00 | 0.27 | 1.00 | 0.33 | 1.00 | 0.39 | 1.00 |
|  | rs72907727 | 185748802 | 0.76 | 1.00 | 0.89 | 1.00 | 0.33 | 1.00 | 0.39 | 1.00 |
|  | rs72907728 | 185749875 | 0.76 | 1.00 | 0.27 | 1.00 | 0.33 | 1.00 | 0.39 | 1.00 |
|  | rs34044253 | 185750030 | 0.94 | 1.00 | 0.60 | 1.00 | 0.08 | 1.00 | 0.13 | 1.00 |
|  | rs62200793 | 185750642 | 0.17 | 1.00 | 0.29 | 1.00 | 0.65 | 1.00 | 0.23 | 1.00 |
|  | rs11894088 | 185750706 | 0.97 | 1.00 | 0.79 | 1.00 | 0.34 | 1.00 | 0.36 | 1.00 |
|  | rs11904676 | 185750720 | 0.94 | 1.00 | 0.62 | 1.00 | 0.61 | 1.00 | 0.36 | 1.00 |
|  | rs111734715 | 185751025 | 0.30 | 1.00 | 0.79 | 1.00 | 0.75 | 1.00 | 0.24 | 1.00 |
|  | rs139794794 | 185751309 | 0.76 | 1.00 | 0.50 | 1.00 | 0.33 | 1.00 | 0.39 | 1.00 |
|  | rs56310614 | 185751960 | 0.76 | 1.00 | 0.27 | 1.00 | 0.33 | 1.00 | 0.39 | 1.00 |
|  | rs113993502 | 185752482 | 0.06 | 1.00 | 0.62 | 1.00 | 0.73 | 1.00 | 0.13 | 1.00 |
|  | rs55914275 | 185752599 | 0.94 | 1.00 | 0.27 | 1.00 | 0.08 | 1.00 | 0.13 | 1.00 |
|  | rs11899082 | 185754482 | 0.94 | 1.00 | 0.27 | 1.00 | 0.61 | 1.00 | 0.36 | 1.00 |
|  | rs114385979 | 185754801 | 0.91 | 1.00 | 0.27 | 1.00 | 0.26 | 1.00 | 0.40 | 1.00 |
|  | rs150700380 | 185755435 | 0.97 | 1.00 | 0.00 | 1.00 | 0.14 | 1.00 | 0.81 | 1.00 |
|  | rs72907735 | 185756295 | 0.85 | 1.00 | 0.13 | 1.00 | 0.22 | 1.00 | 0.26 | 1.00 |
|  | rs13429132 | 185756725 | 0.94 | 1.00 | 0.31 | 1.00 | 0.08 | 1.00 | 0.13 | 1.00 |
|  | rs13428955 | 185756744 | 0.94 | 1.00 | 0.99 | 1.00 | 0.08 | 1.00 | 0.13 | 1.00 |
|  | rs12693399 | 185757011 | 0.57 | 1.00 | 0.22 | 1.00 | 0.60 | 1.00 | 0.31 | 1.00 |
|  | 2:185758261:I | 185758261 | 0.95 | 1.00 | 0.55 | 1.00 | 0.64 | 1.00 | 0.36 | 1.00 |
|  | rs12693400 | 185758412 | 0.94 | 1.00 | 0.22 | 1.00 | 0.61 | 1.00 | 0.36 | 1.00 |
|  | rs6715910 | 185758901 | 0.94 | 1.00 | 0.32 | 1.00 | 0.08 | 1.00 | 0.13 | 1.00 |
|  | rs34700435 | 185758973 | 0.23 | 1.00 | 0.44 | 1.00 | 0.34 | 1.00 | 0.57 | 1.00 |
|  | 2:185759660:I | 185759660 | 0.12 | 1.00 | 0.22 | 1.00 | 0.87 | 1.00 | 0.53 | 1.00 |
|  | rs12999617 | 185759669 | 0.95 | 1.00 | 0.55 | 1.00 | 0.64 | 1.00 | 0.36 | 1.00 |
|  | rs12999259 | 185759675 | 0.95 | 1.00 | 0.33 | 1.00 | 0.08 | 1.00 | 0.13 | 1.00 |
|  | rs13402566 | 185759964 | 0.78 | 1.00 | 0.22 | 1.00 | 0.09 | 1.00 | 0.36 | 1.00 |
|  | rs113113034 | 185760985 | 0.57 | 1.00 | 0.79 | 1.00 | 0.64 | 1.00 | 0.77 | 1.00 |
|  | rs6718067 | 185761264 | 0.94 | 1.00 | 0.43 | 1.00 | 0.61 | 1.00 | 0.36 | 1.00 |
|  | rs72907741 | 185761731 | 0.76 | 1.00 | 0.53 | 1.00 | 0.33 | 1.00 | 0.39 | 1.00 |
|  | rs7584503 | 185761922 | 0.94 | 1.00 | 0.51 | 1.00 | 0.61 | 1.00 | 0.36 | 1.00 |
|  | 2:185762137:I | 185762137 | 0.38 | 1.00 | 0.55 | 1.00 | 0.84 | 1.00 | 0.76 | 1.00 |
|  | rs116715697 | 185762217 | 0.64 | 1.00 | 0.55 | 1.00 | 0.60 | 1.00 | 0.92 | 1.00 |
|  | rs6722425 | 185762587 | 0.94 | 1.00 | 0.22 | 1.00 | 0.08 | 1.00 | 0.13 | 1.00 |
|  | rs6710286 | 185762811 | 0.94 | 1.00 | 0.53 | 1.00 | 0.08 | 1.00 | 0.13 | 1.00 |
|  | 2:185763130:I | 185763130 | 0.93 | 1.00 | 0.40 | 1.00 | 0.62 | 1.00 | 0.50 | 1.00 |
|  | rs3931790 | 185763376 | 0.17 | 1.00 | 0.79 | 1.00 | 0.47 | 1.00 | 0.23 | 1.00 |
|  | rs7562384 | 185763445 | 0.94 | 1.00 | 0.55 | 1.00 | 0.08 | 1.00 | 0.13 | 1.00 |
|  | rs72907747 | 185765063 | 0.76 | 1.00 | 0.00 | 1.00 | 0.33 | 1.00 | 0.39 | 1.00 |
|  | rs1344707 | 185766001 | 0.94 | 1.00 | 0.22 | 1.00 | 0.53 | 1.00 | 0.19 | 1.00 |
|  | rs7603001 | 185766816 | 0.39 | 1.00 | 0.79 | 1.00 | 0.82 | 1.00 | 0.75 | 1.00 |
|  | rs7590852 | 185766912 | 0.94 | 1.00 | 0.94 | 1.00 | 0.53 | 1.00 | 0.19 | 1.00 |
|  | rs12693401 | 185767283 | 0.94 | 1.00 | 0.42 | 1.00 | 0.08 | 1.00 | 0.13 | 1.00 |
|  | rs72907756 | 185767291 | 0.76 | 1.00 | 0.85 | 1.00 | 0.33 | 1.00 | 0.39 | 1.00 |
|  | rs17431722 | 185767753 | 0.11 | 1.00 | 0.59 | 1.00 | 0.93 | 1.00 | 0.37 | 1.00 |
|  | rs1366840 | 185767854 | 0.92 | 1.00 | 0.46 | 1.00 | 0.77 | 1.00 | 0.81 | 1.00 |
|  | 2:185768387:D | 185768387 | 0.03 | 0.95 | 0.60 | 0.96 | 0.28 | 1.00 | 0.19 | 1.00 |
|  | rs10931155 | 185768559 | 0.39 | 1.00 | 0.89 | 1.00 | 0.82 | 1.00 | 0.75 | 1.00 |
|  | rs72907759 | 185769129 | 0.39 | 1.00 | 1.00 | 1.00 | 0.82 | 1.00 | 0.75 | 1.00 |
|  | rs72907760 | 185769130 | 0.39 | 1.00 | 0.16 | 1.00 | 0.82 | 1.00 | 0.75 | 1.00 |
|  | rs10497661 | 185769199 | 0.76 | 1.00 | 0.37 | 1.00 | 0.33 | 1.00 | 0.39 | 1.00 |
|  | rs77076543 | 185769746 | 0.92 | 1.00 | 0.20 | 1.00 | 0.25 | 1.00 | 0.45 | 1.00 |
|  | rs10497662 | 185769921 | 0.80 | 1.00 | 0.98 | 1.00 | 0.26 | 1.00 | 0.44 | 1.00 |
|  | rs1835172 | 185770241 | 0.39 | 1.00 | 0.63 | 1.00 | 0.82 | 1.00 | 0.75 | 1.00 |
|  | rs4666994 | 185771745 | 0.94 | 1.00 | 0.06 | 1.00 | 0.53 | 1.00 | 0.19 | 1.00 |
|  | rs4666995 | 185771790 | 0.94 | 1.00 | 0.82 | 1.00 | 0.53 | 1.00 | 0.19 | 1.00 |
|  | 2:185772000:D | 185772000 | 0.80 | 1.00 | 0.89 | 1.00 | 0.26 | 1.00 | 0.44 | 1.00 |
|  | rs115515236 | 185772643 | 0.64 | 1.00 | 0.98 | 1.00 | 0.60 | 1.00 | 0.92 | 1.00 |
|  | rs72907766 | 185772976 | 0.80 | 1.00 | 0.80 | 1.00 | 0.26 | 1.00 | 0.44 | 1.00 |
|  | rs66970879 | 185773132 | 0.96 | 1.00 | 0.62 | 1.00 | 0.89 | 1.00 | 0.78 | 1.00 |
|  | rs66726164 | 185773141 | 0.96 | 1.00 | 0.52 | 1.00 | 0.89 | 1.00 | 0.78 | 1.00 |
|  | rs66473204 | 185773243 | 0.37 | 1.00 | 0.42 | 1.00 | 0.90 | 1.00 | 0.76 | 1.00 |
|  | rs17431742 | 185773401 | 0.94 | 1.00 | 0.57 | 1.00 | 0.84 | 1.00 | 0.83 | 1.00 |
|  | rs1114248 | 185774139 | 0.80 | 1.00 | 0.59 | 1.00 | 0.26 | 1.00 | 0.44 | 1.00 |
|  | 2:185774477:D | 185774477 | 0.72 | 1.00 | 0.85 | 1.00 | 0.44 | 1.00 | 0.34 | 1.00 |
|  | rs2115622 | 185775819 | 0.39 | 1.00 | 0.90 | 1.00 | 0.83 | 1.00 | 0.73 | 1.00 |
|  | rs2115621 | 185775827 | 0.39 | 1.00 | 0.85 | 1.00 | 0.83 | 1.00 | 0.73 | 1.00 |
|  | rs72893061 | 185776039 | 0.76 | 1.00 | 0.85 | 1.00 | 0.33 | 1.00 | 0.39 | 1.00 |
|  | rs6757288 | 185776345 | 1.00 | 1.00 | 0.95 | 1.00 | 0.42 | 1.00 | 0.99 | 1.00 |
|  | rs78773484 | 185777032 | 0.40 | 1.00 | 0.85 | 1.00 | 0.70 | 1.00 | 0.39 | 1.00 |
|  | rs7594906 | 185777207 | 0.92 | 1.00 | 0.85 | 1.00 | 0.77 | 1.00 | 0.79 | 1.00 |
|  | rs74499716 | 185777319 | 0.57 | 1.00 | 0.85 | 1.00 | 0.94 | 1.00 | 0.54 | 1.00 |
|  | rs72893066 | 185778156 | 0.76 | 1.00 | 0.58 | 1.00 | 0.33 | 1.00 | 0.39 | 1.00 |
|  | rs725617 | 185778262 | 0.96 | 1.00 | 0.85 | 1.00 | 0.49 | 1.00 | 0.22 | 1.00 |
|  | rs1344706 | 185778428 | 0.74 | 1.00 | 0.42 | 1.00 | 0.67 | 1.00 | 0.47 | 1.00 |
|  | rs13009002 | 185778602 | 0.96 | 1.00 | 0.68 | 1.00 | 0.07 | 1.00 | 0.15 | 1.00 |
|  | rs72893068 | 185778772 | 0.76 | 1.00 | 0.32 | 1.00 | 0.33 | 1.00 | 0.39 | 1.00 |
|  | rs78111949 | 185778810 | 0.13 | 1.00 | 0.26 | 1.00 | 0.96 | 1.00 | 0.49 | 1.00 |
|  | rs11899441 | 185779481 | 0.00 | 0.04 | 0.13 | 0.14 | 0.85 | 1.00 | 0.62 | 1.00 |
|  | rs115456739 | 185779904 | 0.64 | 1.00 | 0.23 | 1.00 | 0.60 | 1.00 | 0.92 | 1.00 |
|  | rs4666998 | 185780221 | 1.00 | 1.00 | 0.85 | 1.00 | 0.42 | 1.00 | 0.99 | 1.00 |
|  | rs13423388 | 185780225 | 0.96 | 1.00 | 0.02 | 1.00 | 0.07 | 1.00 | 0.15 | 1.00 |
|  | rs72893071 | 185781197 | 0.76 | 1.00 | 0.68 | 1.00 | 0.33 | 1.00 | 0.39 | 1.00 |
|  | rs62198463 | 185781300 | 0.23 | 1.00 | 0.05 | 1.00 | 0.34 | 1.00 | 0.57 | 1.00 |
|  | rs73043287 | 185782186 | 0.06 | 1.00 | 0.42 | 1.00 | 0.96 | 1.00 | 0.21 | 1.00 |
|  | rs7593816 | 185782312 | 0.16 | 1.00 | 0.96 | 1.00 | 0.45 | 1.00 | 0.24 | 1.00 |
|  | rs1583048 | 185783141 | 0.16 | 1.00 | 0.87 | 1.00 | 0.46 | 1.00 | 0.22 | 1.00 |
|  | rs1366839 | 185783666 | 0.99 | 1.00 | 0.51 | 1.00 | 0.43 | 1.00 | 0.97 | 1.00 |
|  | rs1366838 | 185783896 | 0.40 | 1.00 | 0.05 | 1.00 | 0.82 | 1.00 | 0.70 | 1.00 |
|  | rs115151279 | 185784128 | 0.50 | 1.00 | 0.96 | 1.00 | 0.37 | 1.00 | 0.38 | 1.00 |
|  | rs11901504 | 185785610 | 0.99 | 1.00 | 0.96 | 1.00 | 0.42 | 1.00 | 0.98 | 1.00 |
|  | rs11681373 | 185785791 | 0.75 | 1.00 | 0.62 | 1.00 | 0.66 | 1.00 | 0.45 | 1.00 |
|  | rs2059924 | 185785947 | 0.99 | 1.00 | 0.96 | 1.00 | 0.42 | 1.00 | 0.98 | 1.00 |
|  | rs2059923 | 185785974 | 0.99 | 1.00 | 0.55 | 1.00 | 0.42 | 1.00 | 0.98 | 1.00 |
|  | rs56213902 | 185786030 | 0.91 | 1.00 | 0.85 | 1.00 | 0.76 | 1.00 | 0.77 | 1.00 |
|  | rs142562157 | 185786852 | 0.79 | 1.00 | 0.80 | 1.00 | 0.45 | 1.00 | 0.29 | 1.00 |
|  | rs56321552 | 185787483 | 0.05 | 0.99 | 0.51 | 1.00 | 0.34 | 1.00 | 0.13 | 1.00 |
|  | rs1594166 | 185787586 | 0.75 | 1.00 | 0.44 | 1.00 | 0.93 | 1.00 | 0.78 | 1.00 |
|  | rs78706988 | 185787591 | 0.75 | 1.00 | 0.96 | 1.00 | 0.38 | 1.00 | 0.71 | 1.00 |
|  | rs7580993 | 185789236 | 0.97 | 1.00 | 0.96 | 1.00 | 0.40 | 1.00 | 0.74 | 1.00 |
|  | rs1583050 | 185789332 | 0.86 | 1.00 | 0.96 | 1.00 | 0.48 | 1.00 | 0.23 | 1.00 |
|  | rs113405514 | 185790673 | 0.07 | 1.00 | 0.09 | 1.00 | 0.67 | 1.00 | 0.15 | 1.00 |
|  | rs113696648 | 185791234 | 0.87 | 1.00 | 0.96 | 1.00 | 0.30 | 1.00 | 0.43 | 1.00 |
|  | rs1583049 | 185792818 | 0.39 | 1.00 | 0.05 | 1.00 | 1.00 | 1.00 | 0.98 | 1.00 |
|  | rs146520449 | 185793366 | 0.40 | 1.00 | 0.87 | 1.00 | 0.70 | 1.00 | 0.39 | 1.00 |
|  | rs112770868 | 185794123 | 0.30 | 1.00 | 0.96 | 1.00 | 0.86 | 1.00 | 0.40 | 1.00 |
|  | rs4666999 | 185794216 | 0.34 | 1.00 | 0.42 | 1.00 | 0.99 | 1.00 | 0.89 | 1.00 |
|  | rs17431776 | 185794772 | 0.90 | 1.00 | 0.35 | 1.00 | 0.37 | 1.00 | 0.51 | 1.00 |
|  | rs13012303 | 185795563 | 0.58 | 1.00 | 0.98 | 1.00 | 0.39 | 1.00 | 0.17 | 1.00 |
|  | rs6722992 | 185796345 | 0.39 | 1.00 | 0.42 | 1.00 | 0.60 | 1.00 | 0.92 | 1.00 |
|  | rs78866252 | 185796365 | 0.99 | 1.00 | 0.08 | 1.00 | 0.47 | 1.00 | 0.19 | 1.00 |
|  | rs6723392 | 185796501 | 0.48 | 1.00 | 0.08 | 1.00 | 0.74 | 1.00 | 0.85 | 1.00 |
|  | rs6751736 | 185796571 | 0.37 | 1.00 | 0.03 | 1.00 | 0.96 | 1.00 | 0.92 | 1.00 |
|  | rs6726421 | 185796842 | 0.21 | 1.00 | 0.03 | 1.00 | 0.57 | 1.00 | 0.66 | 1.00 |
|  | rs72893092 | 185796995 | 0.97 | 1.00 | 0.35 | 1.00 | 0.40 | 1.00 | 0.74 | 1.00 |
|  | rs13025111 | 185797159 | 0.21 | 1.00 | 0.16 | 1.00 | 0.57 | 1.00 | 0.66 | 1.00 |
|  | rs6755404 | 185797228 | 0.54 | 1.00 | 0.42 | 1.00 | 0.49 | 1.00 | 0.36 | 1.00 |
|  | rs17509873 | 185797408 | 0.90 | 1.00 | 0.08 | 1.00 | 0.37 | 1.00 | 0.51 | 1.00 |
|  | rs17431804 | 185797580 | 0.99 | 1.00 | 0.51 | 1.00 | 0.27 | 1.00 | 0.39 | 1.00 |
|  | rs10931156 | 185797687 | 0.39 | 1.00 | 0.39 | 1.00 | 0.60 | 1.00 | 0.92 | 1.00 |
|  | rs74620122 | 185798066 | 0.85 | 1.00 | 0.07 | 1.00 | 0.94 | 1.00 | 0.89 | 1.00 |
|  | rs4667000 | 185798504 | 0.21 | 1.00 | 0.77 | 1.00 | 0.57 | 1.00 | 0.66 | 1.00 |
|  | rs72893100 | 185798766 | 0.96 | 1.00 | 0.44 | 1.00 | 0.43 | 1.00 | 0.65 | 1.00 |
|  | 2:185800455:I | 185800455 | 0.31 | 1.00 | 0.59 | 1.00 | 0.92 | 1.00 | 0.97 | 1.00 |
|  | rs12476147 | 185800905 | 0.38 | 1.00 | 0.83 | 1.00 | 0.65 | 1.00 | 0.96 | 1.00 |
|  | rs61739291 | 185801103 | 0.09 | 1.00 | 0.88 | 1.00 | 0.46 | 1.00 | 0.78 | 1.00 |
|  | rs112386296 | 185801470 | 0.56 | 1.00 | 0.09 | 1.00 | 0.88 | 1.00 | 0.31 | 1.00 |
|  | rs35676856 | 185801559 | 0.98 | 1.00 | 0.52 | 1.00 | 0.45 | 1.00 | 0.73 | 1.00 |
|  | rs61739290 | 185801597 | 0.98 | 1.00 | 0.36 | 1.00 | 0.45 | 1.00 | 0.73 | 1.00 |
|  | rs4667001 | 185801747 | 0.21 | 1.00 | 0.49 | 1.00 | 0.60 | 1.00 | 0.72 | 1.00 |
|  | rs4667002 | 185801755 | 0.52 | 1.00 | 0.42 | 1.00 | 0.51 | 1.00 | 0.40 | 1.00 |
|  | rs728534 | 185801917 | 0.08 | 1.00 | 0.42 | 1.00 | 0.28 | 1.00 | 0.97 | 1.00 |
|  | rs61739287 | 185802184 | 0.18 | 1.00 | 0.23 | 1.00 | 0.68 | 1.00 | 0.82 | 1.00 |
|  | 2:185802209:I | 185802209 | 0.09 | 1.00 | 0.03 | 1.00 | 0.70 | 1.00 | 0.87 | 1.00 |
|  | rs1366842 | 185802243 | 0.21 | 1.00 | 0.07 | 1.00 | 0.60 | 1.00 | 0.72 | 1.00 |
|  | rs12477430 | 185802363 | 0.35 | 1.00 | 0.42 | 1.00 | 0.98 | 1.00 | 0.79 | 1.00 |
|  | rs3731834 | 185803364 | 0.91 | 1.00 | 0.08 | 1.00 | 0.88 | 1.00 | 0.63 | 1.00 |
|  | rs112183442 | 185803445 | 0.80 | 1.00 | 0.42 | 1.00 | 0.45 | 1.00 | 0.35 | 1.00 |
|  | rs62198467 | 185804581 | 0.21 | 1.00 | 0.71 | 1.00 | 0.38 | 1.00 | 0.41 | 1.00 |
|  | 2:185805069:D | 185805069 | 0.37 | 1.00 | 0.58 | 1.00 | 0.50 | 1.00 | 0.45 | 1.00 |
|  | rs144576268 | 185805087 | 0.48 | 1.00 | 0.51 | 1.00 | 0.62 | 1.00 | 0.61 | 1.00 |
|  | rs10931157 | 185805453 | 0.41 | 1.00 | 0.06 | 1.00 | 0.72 | 1.00 | 0.97 | 1.00 |
|  | rs142192346 | 185805593 | 0.03 | 0.95 | 0.23 | 0.97 | 0.32 | 1.00 | 0.16 | 1.00 |
|  | rs34221271 | 185805900 | 0.58 | 1.00 | 0.03 | 1.00 | 0.44 | 1.00 | 0.27 | 1.00 |
|  | rs62198468 | 185806340 | 0.58 | 1.00 | 0.42 | 1.00 | 0.44 | 1.00 | 0.27 | 1.00 |
|  | rs6745533 | 185807024 | 0.38 | 1.00 | 0.28 | 1.00 | 0.52 | 1.00 | 0.50 | 1.00 |
|  | rs116992984 | 185807628 | 0.25 | 1.00 | 0.08 | 1.00 | 0.76 | 1.00 | 0.33 | 1.00 |
|  | rs12693402 | 185808079 | 0.84 | 1.00 | 0.23 | 1.00 | 0.62 | 1.00 | 0.44 | 1.00 |
|  | rs16826341 | 185808151 | 0.25 | 1.00 | 0.82 | 1.00 | 0.76 | 1.00 | 0.33 | 1.00 |
|  | rs72894907 | 185809292 | 0.81 | 1.00 | 0.06 | 1.00 | 0.56 | 1.00 | 0.29 | 1.00 |
|  | rs10931158 | 185809565 | 0.40 | 1.00 | 0.41 | 1.00 | 0.51 | 1.00 | 0.45 | 1.00 |
|  | 2:185809644:D | 185809644 | 0.48 | 1.00 | 0.54 | 1.00 | 0.62 | 1.00 | 0.61 | 1.00 |
|  | rs72894910 | 185809746 | 0.87 | 1.00 | 0.92 | 1.00 | 0.54 | 1.00 | 0.24 | 1.00 |
|  | rs115117982 | 185809820 | 0.48 | 1.00 | 0.06 | 1.00 | 0.62 | 1.00 | 0.61 | 1.00 |
|  | 2:185809822:D | 185809822 | 0.74 | 1.00 | 0.50 | 1.00 | 0.49 | 1.00 | 0.31 | 1.00 |
|  | rs75009893 | 185811600 | 0.25 | 1.00 | 0.90 | 1.00 | 0.76 | 1.00 | 0.33 | 1.00 |
|  | rs72894912 | 185811831 | 0.87 | 1.00 | 0.60 | 1.00 | 0.54 | 1.00 | 0.24 | 1.00 |
|  | rs4380187 | 185811940 | 0.50 | 1.00 | 0.04 | 1.00 | 0.37 | 1.00 | 0.26 | 1.00 |
|  | rs147837013 | 185814409 | 0.25 | 1.00 | 0.08 | 1.00 | 0.76 | 1.00 | 0.33 | 1.00 |
|  | rs16826345 | 185818286 | 0.25 | 1.00 | 0.20 | 1.00 | 0.76 | 1.00 | 0.33 | 1.00 |
|  | rs182938800 | 185818768 | 0.00 | 0.02 | 0.20 | 0.04 | 0.45 | 1.00 | 0.38 | 1.00 |
|  | 2:185819161:D | 185819161 | 0.55 | 1.00 | 0.63 | 1.00 | 0.10 | 1.00 | 0.41 | 1.00 |
|  | rs185657398 | 185819269 | 0.74 | 1.00 | 0.17 | 1.00 | 0.24 | 1.00 | 0.44 | 1.00 |
|  | rs77982697 | 185819762 | 0.50 | 1.00 | 0.20 | 1.00 | 0.37 | 1.00 | 0.38 | 1.00 |
|  | rs16826346 | 185820093 | 0.24 | 1.00 | 0.08 | 1.00 | 0.62 | 1.00 | 0.23 | 1.00 |
|  | rs10210173 | 185821339 | 0.85 | 1.00 | 0.96 | 1.00 | 0.45 | 1.00 | 0.19 | 1.00 |
|  | rs16826348 | 185822088 | 0.24 | 1.00 | 0.14 | 1.00 | 0.62 | 1.00 | 0.23 | 1.00 |
|  | rs1429428 | 185822703 | 0.68 | 1.00 | 0.06 | 1.00 | 0.27 | 1.00 | 0.09 | 1.00 |
|  | rs79958518 | 185822995 | 0.57 | 1.00 | 0.20 | 1.00 | 0.64 | 1.00 | 0.77 | 1.00 |
|  | rs138688278 | 185823098 | 0.24 | 1.00 | 0.20 | 1.00 | 0.62 | 1.00 | 0.23 | 1.00 |
|  | rs6434107 | 185823466 | 0.85 | 1.00 | 0.24 | 1.00 | 0.45 | 1.00 | 0.19 | 1.00 |
|  | rs7591741 | 185823879 | 0.61 | 1.00 | 0.20 | 1.00 | 0.34 | 1.00 | 0.38 | 1.00 |
|  | rs115786539 | 185824086 | 0.24 | 1.00 | 0.20 | 1.00 | 0.62 | 1.00 | 0.23 | 1.00 |
|  | rs72894918 | 185824814 | 0.48 | 1.00 | 0.63 | 1.00 | 0.62 | 1.00 | 0.61 | 1.00 |
|  | rs7569641 | 185825253 | 0.73 | 1.00 | 0.69 | 1.00 | 0.38 | 1.00 | 0.26 | 1.00 |
|  | rs72894920 | 185825290 | 0.79 | 1.00 | 0.63 | 1.00 | 0.48 | 1.00 | 0.23 | 1.00 |
|  | rs116624576 | 185825767 | 0.77 | 1.00 | 0.23 | 1.00 | 0.15 | 1.00 | 0.36 | 1.00 |
|  | rs72894921 | 185826405 | 0.85 | 1.00 | 0.20 | 1.00 | 0.45 | 1.00 | 0.19 | 1.00 |
|  | rs72894925 | 185826524 | 0.86 | 1.00 | 0.35 | 1.00 | 0.45 | 1.00 | 0.23 | 1.00 |
|  | rs145898111 | 185826706 | 0.25 | 1.00 | 0.20 | 1.00 | 0.61 | 1.00 | 0.30 | 1.00 |
|  | rs72894927 | 185826749 | 0.80 | 1.00 | 0.20 | 1.00 | 0.47 | 1.00 | 0.27 | 1.00 |
|  | rs7591202 | 185827400 | 0.63 | 1.00 | 0.19 | 1.00 | 0.44 | 1.00 | 0.27 | 1.00 |
|  | 2:185827834:D | 185827834 | 0.48 | 1.00 | 0.74 | 1.00 | 0.62 | 1.00 | 0.61 | 1.00 |
|  | rs1346652 | 185827952 | 0.86 | 1.00 | 0.45 | 1.00 | 0.45 | 1.00 | 0.23 | 1.00 |
|  | rs189773796 | 185827953 | 0.00 | 0.02 | 0.20 | 0.04 | 0.45 | 1.00 | 0.38 | 1.00 |
|  | rs76319116 | 185828785 | 0.25 | 1.00 | 0.20 | 1.00 | 0.61 | 1.00 | 0.30 | 1.00 |
|  | rs72894930 | 185828840 | 0.48 | 1.00 | 0.20 | 1.00 | 0.62 | 1.00 | 0.61 | 1.00 |
